# Ability of pulse oximetry-derived indices to predict hypotension after spinal anesthesia for cesarean delivery: A systematic review and meta-analysis

**DOI:** 10.1371/journal.pone.0316715

**Published:** 2025-01-31

**Authors:** Yuriko Kondo, Eishin Nakamura, Hisashi Noma, Sayuri Shimizu, Takahisa Goto, Takahiro Mihara

**Affiliations:** 1 Department of Health Data Science, Graduate School of Data Science, Yokohama City University, Yokohama, Kanagawa, Japan; 2 The Institute of Statistical Mathematics, Tokyo, Japan; 3 Anaesthesiology and Critical Care Medicine, Yokohama City University School of Medicine Graduate School of Medicine, Yokohama, Kanagawa, Japan; 4 Yokohama Shiritsu Daigaku Igakubu Daigakuin Igaku Kenkyuka, Yokohama, Kanagawa, Japan; The Ohio State University College of Medicine, UNITED STATES OF AMERICA

## Abstract

Cesarean deliveries are often performed under spinal anesthesia because of the reduced risk of complications compared with that of general anesthesia. However, hypotension frequently occurs and adversely affects both the mother and fetus. Indices, such as the perfusion index (PI) and pleth variability index (PVI), which are derived from pulse oximetry have been used in numerous studies to predict hypotension after spinal anesthesia. However, their predictive abilities remain controversial. This study aimed to investigate the ability of PI and PVI, measured before the initiation of spinal anesthesia, to predict hypotension after spinal anesthesia in patients undergoing cesarean deliveries. To this end, we conducted a systematic review and meta-analysis. We searched MEDLINE, Embase, Web of Science, Cochrane Central Register of Controlled Trials, Cochrane Database of Systematic Reviews, ClinicalTrials.gov, European Union Clinical Trials Register, World Health Organization International Clinical Trials Registry Platform, and University Hospital Medical Information Network Clinical Trials Registry databases from inception until June 15, 2023. We included retrospective and prospective observational studies and randomized controlled trials that assessed the ability of PI and PVI, measured before the initiation of spinal anesthesia, to predict hypotension after spinal anesthesia during cesarean delivery. We did not restrict our search to specific languages. Of the 19 studies, involving 1437 patients, 17 assessed the PI in 1,311 patients, and 5 assessed the PVI in 344 patients. The summary sensitivity and specificity of the PI were 0.75 (95% confidence interval [CI]: 0.69–0.80) and 0.64 (95%CI: 0.48–0.77), respectively, while those of the PVI were 0.63 (95%CI: 0.47–0.76) and 0.76 (95%CI: 0.64–0.84), respectively. The area under the summary receiver operating characteristic curve was approximately 0.75 for both indexes. Baseline PI and PVI have a moderate predictive ability for hypotension after spinal anesthesia in patients undergoing cesarean delivery.

## Introduction

Cesarean deliveries are performed under spinal anesthesia because it has a lower risk of complications than general anesthesia [1]. One prevalent side effect of spinal anesthesia is hypotension, which results from sympathetic block, with an estimated incidence of up to 70% [2]. Hypotension causes nausea and vomiting in the mother while it decreases the Apgar score and increases acidosis in the fetus. Moreover, an extended duration of hypotension may affect the neurological prognosis in the fetus [3]. Therefore, it is imperative to prevent and manage hypotension following spinal anesthesia for cesarean delivery.

Owing to the high incidence of hypotension in this context, a consensus statement recommends the prophylactic administration of vasopressors to prevent this condition. However, this recommendation does not apply to patients who are less susceptible to hypotension and those with cardiovascular conditions [3]. Although prophylactic vasopressor infusion reduces the risk of hypotension before and after cesarean delivery, [4] several studies have raised concerns regarding its potential adverse effects, including reactive hypertension and arrhythmia, [5, 6] even in healthy pregnant women. Thus, it is important to carefully assess the risk-benefit balance and avoid unnecessary administration of prophylactic vasopressors, or consider administering them at lower doses in patients with a low risk of hypotension. In this context, the precise prediction of hypotension is vital for optimizing patient care during cesarean delivery.

The perfusion index (PI) and pleth variability index (PVI) can be obtained using pulse oximetry. The PI is an index of peripheral vascular tone [7–9]. A higher baseline PI may reflect lower peripheral vascular tone and probably a decrease in preload due to blood pooling in the lower body [8]. Hence, patients with a high PI may be more prone to hypotension. PVI is an index of circulating blood volume based on respiratory variations in the PI [7]. The PVI reflects fluid responsiveness in patients with spontaneous breathing [10]. Hypovolemic conditions lead to an increased PVI. It is plausible that a higher PVI represents a greater hypotensive effect of spinal anesthesia in patients undergoing cesarean delivery [11].

Although several studies have used pulse oximetry-derived indices to predict hypotension after spinal anesthesia, the predictive ability of these indices remains controversial, with some studies suggesting their usefulness, while others do not [7, 8, 11, 12].

Identifying the predictive ability of PI and PVI for hypotension could provide a basis for how clinical anesthetists interpret the risk of hypotension using PI and PVI prior to anesthesia. This systematic review and meta-analysis aimed to evaluate the existing literature on the ability of the PI and PVI, measured before the initiation of spinal anesthesia, to predict hypotension following spinal anesthesia during cesarean deliveries. These background details are also provided in our previously published protocol paper [13].

## Materials and methods

This manuscript was prepared as per the recommendations of the Preferred Reporting Items for a Systematic Review and Meta-analysis of Diagnostic Test Accuracy Studies (PRISMA-DTA) [14]. The protocol for this systematic review was registered in PROSPERO, registration number CRD42022362596, on October 5, 2022, and in the University Hospital Medical Information Network (UMIN), registration number UMIN000049090, on October 1, 2022. The protocol has been published previously [13]. Ethical approval was not required because this systematic review used previously published data.

### Eligibility criteria

#### Study design

We included retrospective and prospective observational studies, as well as randomized controlled trials, without restricting our search to specific languages. Case reports, case series, and animal studies were excluded.

#### Patients

We included studies examining patients who underwent cesarean deliveries under spinal anesthesia.

#### Index tests

The index tests were the PI and PVI, which were derived from pulse oximetry. The PI is an index of peripheral vascular tone that is calculated as the ratio of pulsatile to non-pulsatile components of the arterial blood based on differential absorption of infrared light and is expressed as a percentage, and the value ranges between 0.02% and 20% [7, 15]. The PVI is an index of the circulating blood volume, based on respiratory variations in the PI. The PVI is a measure of the dynamic change in the PI throughout the respiratory cycle and is calculated using the following formula:

$\text{PVI}=\left(\left[\text{maximum},\;,\text{perfusion},\;,\text{index},-,\text{minimum},\;,\text{perfusion},\;,\text{index}\right],/,\text{maximum},\;,\text{perfusion},\;,\text{index}\right)\times 100$ [7]. As noted earlier, patients with a higher baseline PI may be more prone to hypotension, and higher PVI represents a greater hypotensive effect of spinal anesthesia.

#### Target condition

The target condition was hypotension after the initiation of spinal anesthesia for cesarean delivery. The definition of hypotension was based on that used in each primary study.

### Information sources and search strategy

We searched MEDLINE, Embase, Web of Science, Cochrane Central Register of Controlled Trials, Cochrane Database of Systematic Reviews, ClinicalTrials.gov, European Union Clinical Trials Register, WHO International Clinical Trials Registry Platform, and UMIN databases for appropriate studies. Moreover, we manually searched the reference lists of the relevant articles. The final search was conducted on June 15, 2023. During a pre-publication check of the references on September 20, 2024, we identified one retracted article, which was excluded. The search strategies used for each database were based on a previously published protocol [13].

### Study records

#### Data management and selection process

Two authors (YK and EN) independently screened titles and abstracts. We used Endnote to remove duplicates and exported the remaining titles to Rayyan [16]. When study eligibility could not be determined based on the title or abstract, the full texts were retrieved.

#### Data collection

Two authors (YK and EN) extracted data independently and in duplicate from each eligible study. Any disagreements regarding the eligibility or extracted data were resolved through discussion. When necessary, we contacted the study authors to obtain detailed data when the studies reported only an association between PI and PVI and hypotension. After obtaining the details, we determined the counts of true-positives, true-negatives, false-positives, and false-negatives based on a predetermined threshold value and subsequently incorporated these into our meta-analysis. We used predefined threshold values of 3.5 and 20 for PI and PVI, respectively. If the authors had used a predefined threshold value, we adopted that specific threshold value as reported in their study. For studies that conducted index test measurements at various positions, we consistently selected the results obtained from the mostly in supine position.

#### Data items

A data collection sheet was created to record the following information: author information; year of publication; study design; eligibility criteria; exclusion criteria; type of index test (PI or PVI); cut-off value of index test; definition of hypotension; type of reference standard; the total number of patients; the total number of true-positives, false-positives, false-negatives, and true-negatives in each test; type and dose of the anesthetic drug used; type of anesthesia (spinal anesthesia or combined spinal epidural anesthesia); prophylactic administration of vasopressors; and prophylactic fluid loading.

### Outcomes and prioritization

#### Primary outcome

The primary outcome was the ability of baseline PI and PVI to predict hypotension after spinal anesthesia for cesarean deliveries. We report summary predictive measures, such as sensitivity, specificity, and summary receiver operating characteristic (sROC) curves.

#### Risk-of-bias in individual studies

The risk-of-bias was assessed using the Quality Assessment of Diagnostic Accuracy Studies (QUADAS-2) tool [17]. The QUADAS-2 has four domains: patient selection, index test, reference standard, and flow and timing. Two reviewers independently assessed the risk-of-bias of the studies included. Disagreements between them were resolved through discussion.

### Data synthesis

First, we summarized the outcome measures in individual studies based on the number of true-positives, false-positives, false-negatives, and true-negatives. We calculated the sensitivities, specificities, and corresponding 95% confidence intervals (CIs). We presented coupled forest plots to depict sensitivities and specificities for PI and PVI and generated forest plots for the pairs of sensitivities and specificities to undertake initial exploratory evaluations and assess heterogeneities.

We then performed synthesis analyses using Reitsma’s bivariate random effects model for study-specific sensitivities and specificities [18] to address possible heterogeneity across studies. Based on the results of the synthesis analyses, we estimated the summary sensitivities and specificities and created sROC curves [19]. We assessed heterogeneity using the bivariate version of I^2^, considering the correlation between sensitivity and specificity [20]. We presented the areas under the curves (AUCs) of the sROC curves as summary measures of predictive accuracy. For statistical inferences, we used the standard restricted maximum likelihood estimation for Reitsma’s model and the bootstrap method to calculate the 95% CIs of the AUCs of the sROC curves [21].

We considered significant heterogeneity when the I^2^ statistics exceeded 30%. [22] If heterogeneity existed, we planned to perform a subgroup analysis to explore the underlying causes. Subgroup analyses were designed according to the following variables: cut-off values, definition of hypotension, type of reference standard, type and dose of anesthetic drug used, type of anesthesia, presence or absence of prophylactic vasopressor administration, and fluid loading.

To assess potential publication biases, we performed the generalized Egger test for multivariate meta- analysis [23].

We performed sensitivity analyses after excluding studies with a high risk-of-bias. Additionally, we performed post hoc sensitivity analysis after excluding studies that used anesthetics other than hyperbaric bupivacaine.

Statistical analyses were performed using the R software (R Development Core Team, Vienna, Austria) and RStudio (RStudio, Boston, Massachusetts, USA).

## Results

### Search selection and study characteristics

**Fig 1:**
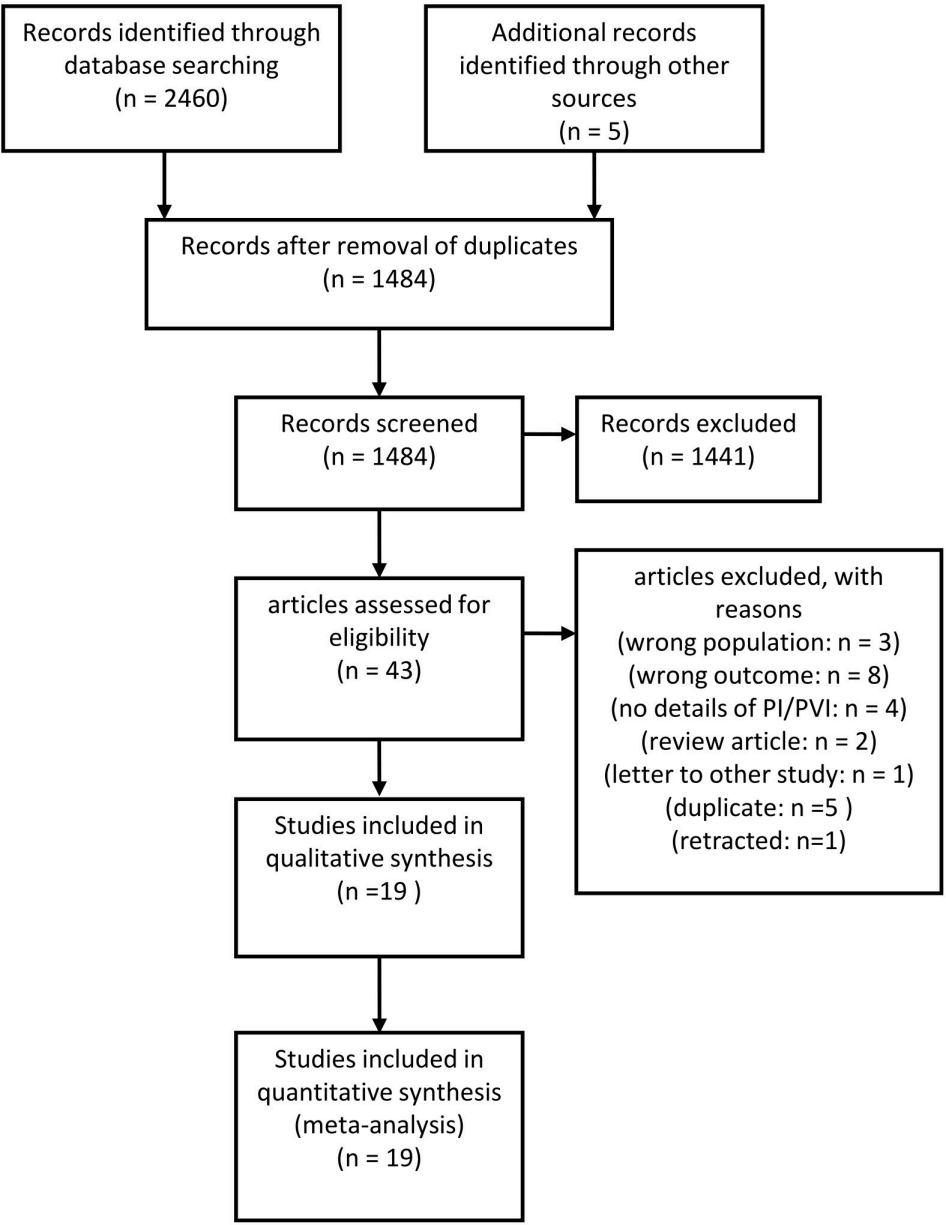
Preferred Reporting Items for Systematic Review and Meta-analysis (PRISMA) flow diagram.

In the initial search, 2,465 studies were identified. We examined the full texts of 43 studies in detail. Of these, 19 studies were included in this systematic review, [7, 8, 11, 12, 15, 24–37] all of which were included in the quantitative synthesis (S1 Table). A PRISMA flow diagram of the retrieved publications is shown in Fig 1. All research data were extracted independently by YK and EN. The initial extraction was conducted between December 24, 2022, and February 28, 2023. A second extraction was performed following a rerun of the search, which took place between June 22, 2023, and September 6, 2023.

The characteristics of the studies included in this systematic review are presented in S2 Table. All the studies were prospective observational studies, and included patients who underwent elective cesarean deliveries. All studies, except those in which it was not mentioned, excluded patients with preeclampsia or pregnancy-induced hypertension. Seventeen studies excluded patients with significant obesity [7, 8, 11, 12, 15, 24–26, 28–31, 33–37]. Seventeen studies excluded patients with cardiovascular disease [7, 8, 11, 12, 15, 25, 26, 28–37]. Fourteen studies used spinal anesthesia, [11, 12, 15, 24–28, 30, 33–37] and 4 used combined spinal–epidural anesthesia (CSEA) [7, 8, 29, 31]. One study used spinal anesthesia, CSEA, or general anesthesia [32]. The reference standard was non-invasive blood pressure monitoring in all the studies, except for three that did not mention the reference standard [27, 32, 35]. The drugs used for spinal anesthesia were hyperbaric bupivacaine in 17 studies, [7, 8, 11, 12, 15, 24–28, 30, 32–37] isobaric bupivacaine in 1, [31] and ropivacaine in 1 [29]. Seven studies involved administration of opioids into the subarachnoid space [7, 8, 11, 12, 15, 32, 35]. The definition of hypotension varied in each primary study; a decrease in systolic blood pressure > 25% of baseline [8, 12, 24] and mean blood pressure < 65 mmHg [15, 30, 36] were commonly used. Patients with painful uterine contractions were excluded in one study, [31] whereas other studies did not mention about it. In most primary studies, index test measurements were performed in the supine position, except for two studies that did not specify the position of the baseline measurement [27, 32] and one study that used the left lateral position [24]. In two studies [26, 28], baseline measurements were conducted in both the sitting and supine positions; we included only the data from the supine position in the analysis.

We contacted the authors of the included studies that did not provide true-positive, false-positive, false-negative, and true-negative information and received two responses: one provided the predetermined threshold values and numbers of true-positives, false-positives, false-negatives, and true-negatives [7]. Another study did not have a predetermined threshold and provided information on PI and PVI values and the presence of hypotension [28]. We counted the number of true-positives, false-positives, false-negatives, and true-negatives based on our *a priori*-determined thresholds and included them in the analyses. We obtained information directly from Dr. Arslan, the author of reference [28], on March 7, 2023, and from Dr. Yokose, the author of reference [7], on March 1, 2023.

### Ability of PI to predict hypotension

**Fig 2:**
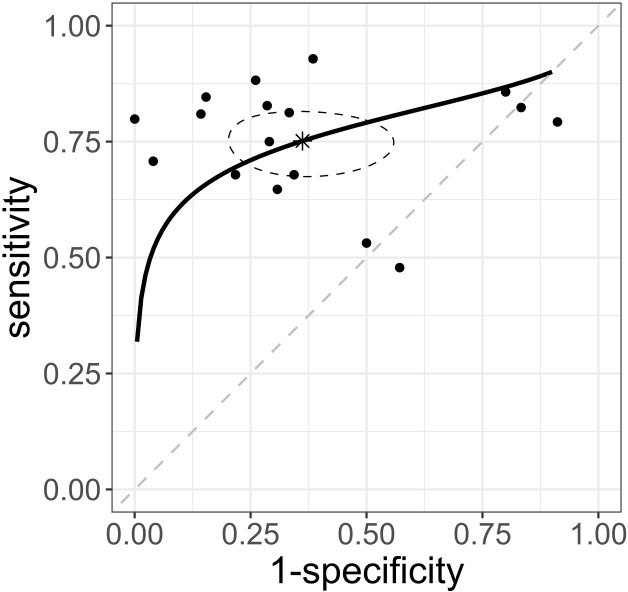
sROC curve of the perfusion index. The solid curves represent sROC curves. The dots represent point estimates of sensitivity and 1-specificity for each study included, and the ellipses represent the 95% confidence region. sROC, summary receiver operating characteristic.

Seventeen studies, [7, 8, 11, 12, 15, 24, 25, 27–30, 32–37] involving 1,311 patients, assessed the predictive ability of PI for hypotension. The sensitivities and specificities of the individual studies, their 95% CIs, and coupled forest plots are shown in S1 Fig. The summary sensitivity was 0.75 (95% CI: 0.69 to 0.80), and summary specificity 0.64 (95% CI: 0.48 to 0.77). The AUC of the sROC curve was 0.76 (95% CI: 0.68 to 0.80; I^2^ statistic 22.7%, Fig 2). Summary estimates of the sensitivity, specificity, AUC of the sROC curve, positive-likelihood ratio (positive LR), and negative-likelihood ratio (negative LR) are shown in Table 1. A funnel plot of the sensitivity and specificity of the PI is presented in S2 Fig. The generalized Egger test result suggested publication bias, with p = 0.0005.

**Table 1:** Summary estimates of sensitivity, specificity, AUC of the sROC curve, positive likelihood ratio, and negative likelihood ratio of perfusion index and pleth variability index.

	Number of patients (study)	Sensitivity (95%CI)	Specificity (95%CI)	AUC of sROC (95%CI)	Positive LR (95%CI)	Negative LR (95%CI)	I^2^
**Perfusion index**	1311 (17)	0.75 (0.69 to 0.80)	0.64 (0.48 to 0.77)	0.76 (0.68 to 0.80)	2.15 (1.43 to 3.30)	0.40 (0.29 to 0.56)	22.7%
**Pleth variability index**	344 (5)	0.63 (0.47 to 0.76)	0.76 (0.64 to 0.84)	0.76 (0.66 to 0.82)	2.60 (1.86 to 3.62)	0.50 (0.34 to 0.67)	0.0%

CI, confidence interval; AUC, area under the curve; sROC, summary receiver operating characteristic; LR, likelihood ratio

### Ability of PVI to predict hypotension

**Fig 3:**
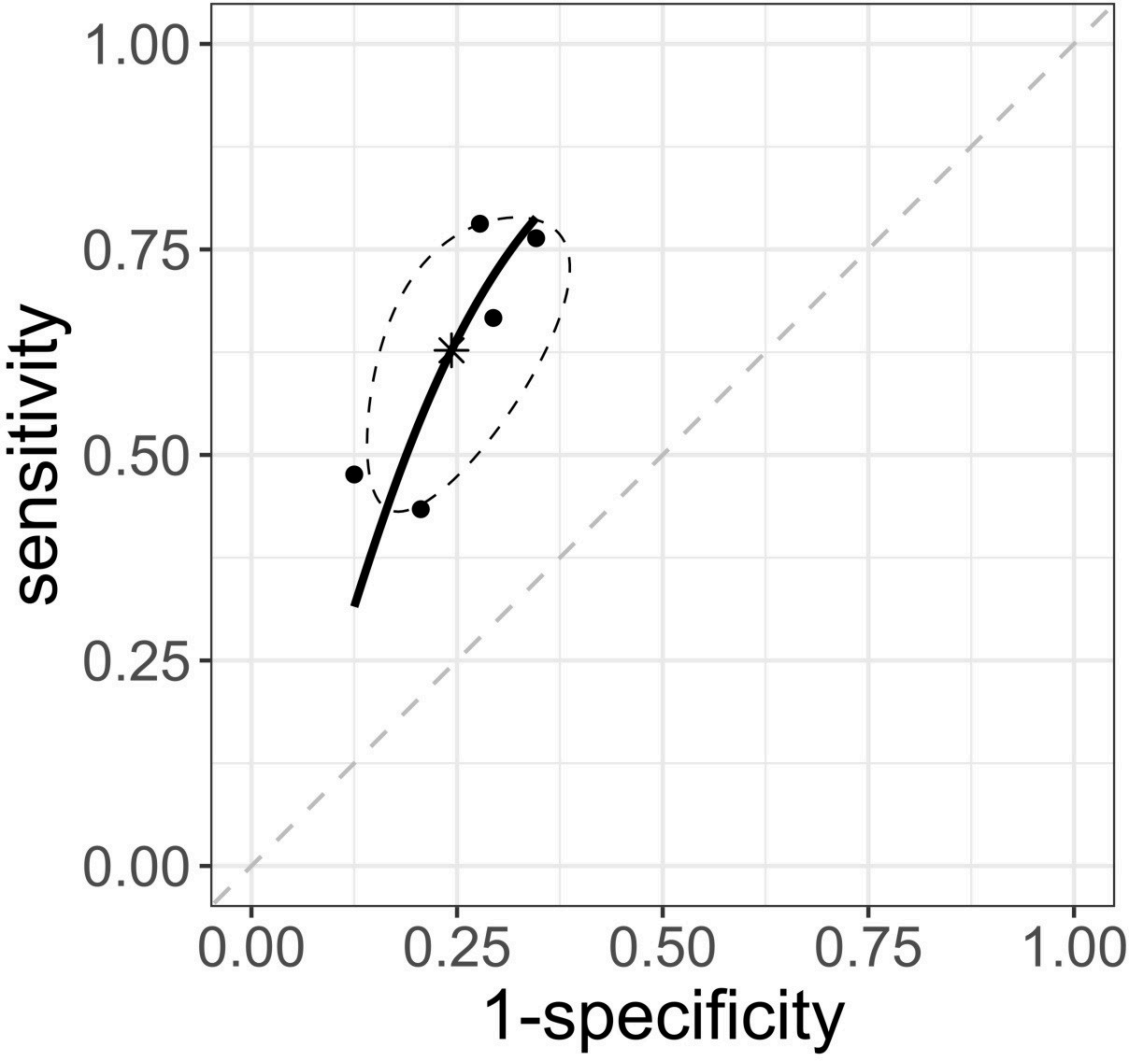
sROC curve of the pleth variability index. The solid curves represent sROC curves. The dots represent point estimates of sensitivity and 1-specificity for each study included, and ellipses represent the 95% confidence region. sROC, summary receiver operating characteristic.

Five studies, [7, 11, 26, 28, 31] involving 344 patients, assessed the predictive ability of PVI for hypotension. The sensitivity and specificity of the individual studies, their 95% CIs, and coupled forest plots are shown in S3 Fig. The summary sensitivity was 0.63 (95% CI: 0.47 to 0.76), and summary specificity 0.76 (95% CI: 0.64 to 0.84). The AUC of the sROC curve was 0.76 (95% CI: 0.66 to 0.82; I^2^ statistic 0%, Fig 3). These results are summarized in Table 1. A funnel plot of the sensitivity and specificity of the PI is presented in S4 Fig. The generalized Egger test result indicated the absence of publication bias, with p = 0.48.

### Quality assessment of individual studies

**Fig 4:**
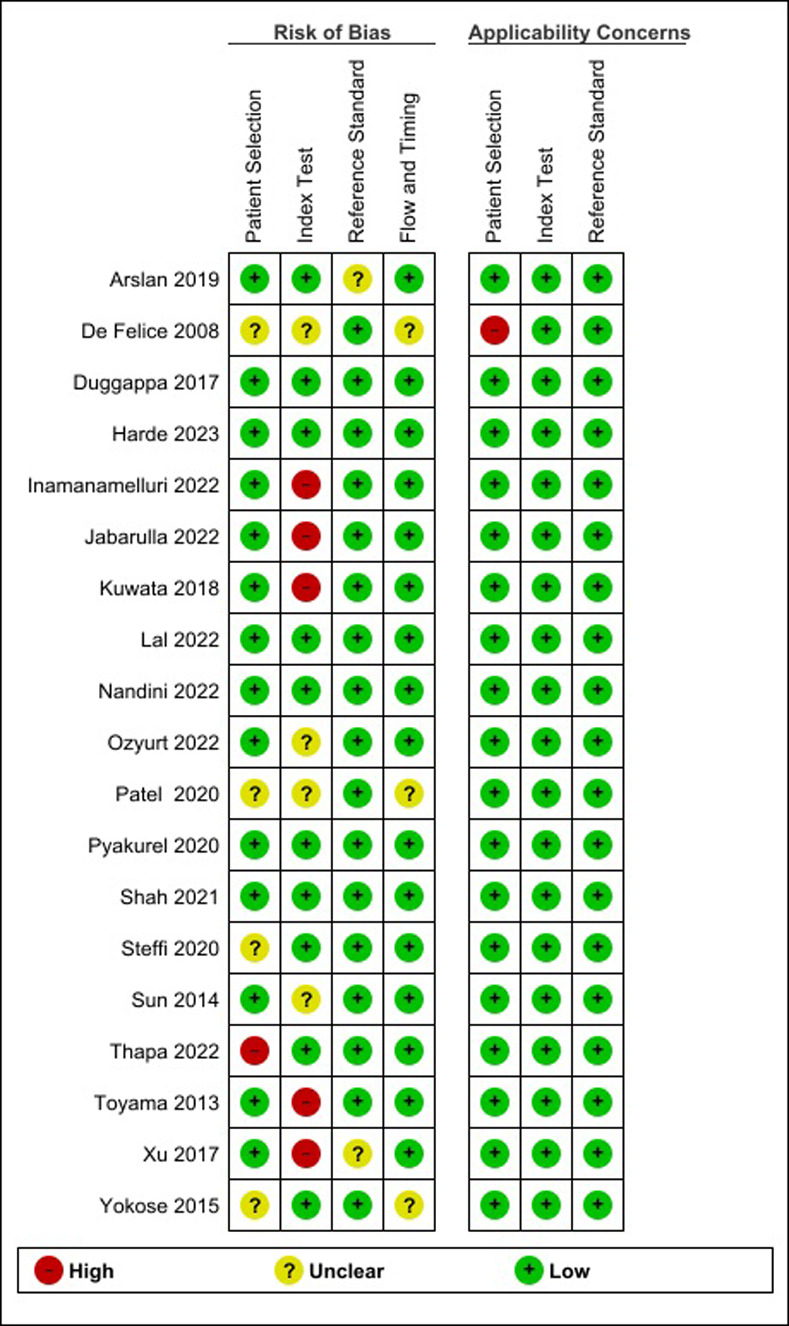
Quality assessment of individual studies.

The QUADAS-2 tool was used to assess the quality of the primary studies. The results are shown in Fig 4. Six studies had a low risk-of-bias in all domains [15, 24, 25, 30, 35, 36]. Five studies were determined to have a high risk-of-bias in the domain of the index test because the cut-off values were not determined *a priori* [8, 11, 12, 29, 33]. One study was considered to have a high risk-of-bias in the patient domain of applicability concerns because they included patients who received anesthesia methods other than spinal anesthesia or CSEA [32].

### Subgroup analysis

Subgroup analysis was not performed owing to low heterogeneity.

### Sensitivity analysis

We performed sensitivity analysis after excluding studies with a high risk-of-bias and conference abstracts. Nine studies, [7, 15, 24, 25, 28, 30, 34–36] involving 677 patients, were included in the sensitivity analysis of PI, and 4, [7, 26, 28, 31] involving 294 patients, were included in the sensitivity analysis of PVI. The results of the sensitivity analysis were similar to those of the main analysis (S5, S6 Figs and S3 Table).

We performed post-hoc sensitivity analysis after excluding studies that used anesthetics other than hyperbaric bupivacaine [29, 31]. Sixteen studies involving 1217 patients were included in the sensitivity analysis of PI, and 4 involving 262 patients were included in the sensitivity analysis of PVI. The results of the post-hoc sensitivity analysis were similar to those of the main analysis (S7, S8 Figs and S4 Table).

## Discussion

This systematic review included 19 studies involving 1,437 patients who underwent cesarean deliveries under spinal anesthesia and evaluated the ability of PI and PVI to predict hypotension after spinal anesthesia. Our meta-analysis showed that the AUCs of the sROC curves were approximately 0.75, which indicated a moderate predictive ability for hypotension after spinal anesthesia in patients undergoing cesarean delivery. These values, derived from pulse oximetry, have the advantage of being obtained using a non-invasive approach in a relatively short time. This advantage facilitates clinicians’ access to real-time objective data and enhances the utility of these indices in clinical scenarios.

With an AUC of approximately 0.75, the predictive accuracy of PI and PVI might be suboptimal, if used independently. The positive LR for PI was 2.15, whereas the negative LR was 0.40. The clinical interpretation is that, assuming a 50% pretest probability of post spinal anesthesia hypotension in patients undergoing cesarean delivery, the subsequent post-test probability was approximately 68% for a positive PI and 29% for a negative PI, suggesting that PI may be insufficient to provide conclusive evidence for predicting hypotension. Conventionally, a positive LR above 10 and an negative LR below 0.1 are recognized as benchmarks in clinical practice [38, 39]. Given the cut-off values we employed, both PI and PVI demonstrated likelihood ratios that did not meet the thresholds for optimal predictive relevance. Hence, additional research is warranted for the effective clinical application of PI and PVI in this context.

We propose two primary directions for subsequent research. First, we assessed the potential of integrating PI and PVI with other variables to refine predictive accuracy. Additionally, the combination of PI and PVI itself may also warrant evaluation. Previous studies have reported that several factors, including maternal weight gain during pregnancy and baseline heart rate, [7, 40] may be useful to predict hypotension. These factors may be considered as candidate variables in combination with PI and PVI. Second, PI and PVI should be considered tools for identifying low-risk patients, with an emphasis on negative LR, and a cut-off value for PI and PVI that yields an adequate negative LR (potentially 0.1) should be determined. If a cut-off that results in an negative LR ratio < 0.1 could be established, patients exhibiting negative results from PI or PVI tests could be designated as low-risk. These patients may not necessarily require prophylactic vasopressor administration, particularly if there are concerns or reasons to hesitate in administering vasopressors. In contrast, other patients may be advised to use vasopressors, in line with established consensus statements [3].

Previous study results have varied. Dynamic indicators, such as PI and PVI, may be affected by body position, room temperature, and mental state at the time of measurement, which may partly explain the variability in the results obtained to date. [11, 28] Therefore, efforts should be made to limit the variability of external factors. Future studies should establish a feasible and uniform baseline measurement environment that is acceptable in many situations. The baseline measurement environment should be stated in the study.

The strength of this study is that it quantitatively integrated the predictive ability of pulse oximetry-derived indices for hypotension after spinal anesthesia in patients undergoing cesarean deliveries. Although a systematic review evaluating PI and PVI as a predictive index for hypotension after spinal anesthesia has been published, [41] no quantitatively synthesized results regarding its predictive ability were presented. We have presented such results in this study.

This study has some limitations. First, although we did not intentionally restrict the patient population, most studies included in this systematic review excluded patients with preeclampsia or pregnancy-induced hypertension, obesity, or cardiovascular diseases. As a result, the findings of this systematic review cannot be extrapolated to these patients, limiting the generalizability of our results to broader populations. Our systematic review also revealed a lack of research on the prediction of hypotension using PI and PVI in these patients. Consequently, further studies are warranted in this regard. Second, it is unclear whether interventions based on PI and PVI predictions, such as risk-based prophylactic administration of vasoconstrictors, improve fetal Apgar scores or alleviate maternal hypotension-associated symptoms. Future studies are needed to develop protocols for administering vasopressors based on these predictions and to investigate whether their implementation improves maternal and neonatal outcomes. Third, the cut off points varied in each of the primary studies included in this systematic review. For PI, the cutoff values ranged from 1.75 to 4.0, with 76% (13/17) of the studies having cutoff values between 3.0 and 4.0, which is a relatively narrow range. For PVI, the cutoff values ranged from 18 to 23.5, also within a narrow range. From a clinical perspective, the cutoff values are relatively consistent; however, our study results cannot be extrapolated to cutoff values outside of these ranges. Fourth, the definition of hypotension varied in each of the primary studies included in this systematic review. This limits data interpretation. However, the integrated results showed low heterogeneity. This may indicate that although the definitions of hypotension in each study were slightly different, they did not represent a source of heterogeneity, i.e. they indicated similar situations. Therefore, as long as we consider a similar definition to those used in each of the primary studies, it should be possible to extrapolate the results of this study, even if there are some differences in the definition of hypotension. In contrast, if a definition not used in the included studies—such as one that employs a lower cut-off blood pressure to focus exclusively on patients with more severe hypotension—is applied, extrapolation of the findings may not be feasible. Using a lower cut-off value could reduce the number of patients diagnosed with hypotension, potentially diminishing the specificity of the results. Fifth, the small number of studies for assessing PVI (N = 5) may influence the stability of the estimations in the Reitsma’s random-effects model and the sROC curve. Sixth, publication bias existed among the included studies. The results of this meta-analysis may overestimate the predictive accuracy.

## Conclusion

We demonstrated that baseline PI and PVI had moderate predictive ability for hypotension after spinal anesthesia in patients undergoing cesarean delivery. The area under the summary receiver operating characteristic curve was approximately 0.75 for both indices. Future studies are needed to integrate PI and PVI, or their combination, with other variables to improve predictive accuracy. Research is also needed to determine cut-off values for PI and PVI that would result in an appropriate negative LR. In addition, to clarify the clinical value of predicting hypotension, it is essential to develop protocols for administering vasopressors based on these predictions and to investigate whether their implementation improves maternal and neonatal outcomes.

## Supporting information

S1 Table Studies identified in the literature search. (PDF)

S2 Table The characteristics of the studies included in this systematic review. (DOCX)

S3 Table Summary estimates of sensitivity, specificity, AUC of the sROC curve, positive likelihood ratio, and negative likelihood ratio of sensitivity analysis for perfusion index and pleth variability index. CI; confidence interval, AUC; area under curve, sROC; summary receiver operating characteristic curve.
(DOCX)

S4 Table Summary estimates of sensitivity, specificity, AUC of the sROC curve, positive likelihood ratio, and negative likelihood ratio of posthoc sensitivity analysis for perfusion index and pleth variability index. CI; confidence interval, AUC; area under curve, sROC; summary receiver operating characteristic curve.
(DOCX)

S1 Fig Summarized outcome measures in individual studies for the perfusion index. (TIF)

S2 Fig (a) Funnel plot of the sensitivity of perfusion index. Se; sensitivity. (b) Funnel plot of the specificity of perfusion index.
(TIF)

S3 Fig Summarized outcome measures in individual studies of the pleth variability index. (PDF)

S4 Fig (a) Funnel plot of the sensitivity of pleth variability index. Se; sensitivity. (b) Funnel plot of the specificity of pleth variability index.
(TIF)

S5 Fig Summary receiver operating characteristic (sROC) curve of sensitivity analysis for perfusion index. The solid curves represent sROC curves. The dots represent point estimates of sensitivity and 1-specificity for each study included, and the ellipses represent the 95% confidence region.
(TIF)

S6 Fig Summary receiver operating characteristic (sROC) curve of sensitivity analysis for pleth variability index. The solid curves represent sROC curves. The dots represent point estimates of sensitivity and 1-specificity for each study included, and the ellipses represent the 95% confidence region.
(TIF)

S7 Fig Summary receiver operating characteristic (sROC) curve of posthoc sensitivity analysis for perfusion index. The solid curves represent sROC curves. The dots represent point estimates of sensitivity and 1-specificity for each study included, and the ellipses represent the 95% confidence region.
(TIF)

S8 Fig Summary receiver operating characteristic (sROC) curve of posthoc sensitivity analysis for pleth variability index. The solid curves represent sROC curves. The dots represent point estimates of sensitivity and 1-specificity for each study included, and the ellipses represent the 95% confidence region.
(TIF)

S1 Checklist PRISMA-DTA checklist. (DOC)
