# Supplementary material for: Ability of pulse oximetry-derived indices to predict hypotension after spinal anesthesia for cesarean delivery: A systematic review and meta-analysis
Source: PLoS One. 2025 Jan 31;20(1):e0316715. doi: 10.1371/journal.pone.0316715 (PMC11785266; doi:10.1371/journal.pone.0316715)
Supplement: S1 Table — (PDF) [file pone.0316715.s001.pdf]

No	Judgement	Reason for Exclusion	Title	year	URLs for Included Studies
1	Exclude	wrong population	A Potential Resuscitation Route on Battlefield: Immediate Intraperitoneal Fluid Administration Post-burn Shows Satisfactory Fluid Absorption and Anti-shock Effects	2023	
2	Exclude	wrong outcome	Abdominal Near Infrared Spectroscopy can be reliably used to measure splanchnic oxygenation changes in preterm infants	2023	
3	Exclude	wrong study design	An atypical case of edema of the umbilical cord and histological chorioamnionitis in a fetus with normal growth and abnormal umbilical artery Doppler: a case report	2023	
4	Exclude	wrong outcome	Association of birth by cesarean section with academic performance and intelligence in youth: A cohort study	2023	
5	Exclude	wrong outcome	Biomarkers of Uteroplacental Insufficiency in COVID-19 Affected Pregnancies.	2023	
6	Exclude	wrong population	Bubble devices versus other pressure sources for nasal continuous positive airway pressure in preterm infants	2023	
7	Exclude	no details of PI or PVI	Cardiovascular effects of oxytocin and carbetocin at cesarean section. A prospective double-blind randomized study using noninvasive pulse wave analysis	2023	
8	Exclude	wrong index test	Colour Doppler and Adverse Perinatal Outcomes in Pregnancies with Foetal Growth Restriction: A Prospective Longitudinal Study	2023	
9	Exclude	wrong outcome	Comparing prenatal and postpartum stress among women with previous adverse pregnancy outcomes and normal obstetric histories: A longitudinal cohort study	2023	
10	Exclude	wrong index test	Comparison of ductus venosus Doppler and cerebroplacental ratio for the prediction of adverse perinatal outcome in high-risk pregnancies before and after 34 weeks	2023	
11	Exclude	wrong outcome	Comparisons of the diagnostic accuracy of the ultrasonic sign-score method and MRI for PA, PI and PP in high-risk gravid women: a retrospective study	2023	
12	Exclude	wrong population	Correlation of Serum Lactate Levels, Perfusion Index and Plethysmography Variability Index With Invasive Blood Pressure in Late Preterm and Term Infants With Shock	2023	
13	Exclude	wrong outcome	CPR is Better Predictor of Intrauterine Growth Restriction as Compare to Individual Artery Examination	2023	
14	Exclude	review article	Current Commonly Used Dynamic Parameters and Monitoring Systems for Perioperative Goal-Directed Fluid Therapy: A Review	2023	

15	Exclude	wrong outcome	Cutaneous Perfusion Dynamics of the Lower Abdomen in Healthy Normal Weight, Overweight and Obese Women: Methods Development Using Infrared Thermography with Applications for Future Wound Management after Caesarean Section	2023	
16	Exclude	wrong outcome	Does umbilical venous diameter or time-averaged maximum velocity correlate better with fetal outcome in growth-restriction?	2023	
17	Exclude	wrong index test	Effect of grade 3 placenta <36 weeks of pregnancy on perinatal	2023	
18	Exclude	wrong outcome	Effectiveness, safety, and acceptability of postplacental insertion of GyneFix postpartum intrauterine device among women undergoing cesarean section: A multicenter prospective cohort study in China	2023	
19	Exclude	wrong outcome	Elevated GABRP expression is correlated to the excessive autophagy in intrahepatic cholestasis of pregnancy	2023	
20	Exclude	wrong outcome	Fetal abdominal adiposity score and the prediction of large for gestational age infants	2023	
21	Exclude	wrong outcome	Fetal Biometric Assessment and Infant Developmental Prognosis of the Tadalafil Treatment for Fetal Growth Restriction	2023	
22	Exclude	wrong population	Fetal doppler role in predicting non-reassuring fetal heart tracing in patients undergoing induction of labor	2023	
23	Exclude	wrong outcome	First trimester screening: the increasing value of the test beyond aneuploidies analysis	2023	
24	Exclude	wrong outcome	Impact of fetal growth restriction on pregnancy outcome in women undergoing expectant management for preterm pre-eclampsia	2023	
25	Exclude	wrong population	Influence of Anesthetics on Clinical Outcome During Cardiac Surgery in Adults	2023	
26	Exclude	wrong outcome	Interventions from pregnancy to two years after birth for parents experiencing complex post-traumatic stress disorder and/or with childhood experience of maltreatment	2023	
27	Exclude	wrong population	Lactoferrin/sialic acid prevents adverse effects of intrauterine growth restriction on neurite length: investigations in an in vitro rabbit neurosphere model	2023	
28	Exclude	wrong outcome	Mesenchymal Stem/Stromal Cells: ON DEMAND MANUFACTURING STRATEGY TO DELIVER MULTIPLE DOSES OF FRESHLY CULTURED UMBILICAL CORD DERIVED MSCS IN THE CELLULAR IMMUNO-THERAPY FOR COVID-19 RELATED ARDS	2023	
29	Exclude	wrong outcome	Methylphenidate for children and adolescents with attention deficit hyperactivity disorder (ADHD)	2023	
30	Exclude	wrong outcome	Mobile health (m-health) technological support for women during pregnancy or the first six weeks postpartum, or both	2023	

31	Exclude	wrong outcome	Outcomes among Neonates after a Diagnosis of Persistent or Transient Fetal Growth Restriction Delivered at Term	2023	
32	Exclude	wrong outcome	Oxytocin changes in women with emergency cesarean section: Association with maternal blues by delivery mode	2023	
33	Include		Perfusion index to predict post spinal hypotension in lower segment caesarean section	2023	https://doi.org/10.4103/joacp.joacp_178_22
34	Exclude	wrong outcome	Phenylephrine and Ephedrine for Prevention of Hypotension in Women during Lower Segment Caesarean Section under Spinal Anaesthesia: A Randomised Clinical Study	2023	
35	Exclude	wrong population	Physical exercise for people with Parkinson's disease: a systematic review and network meta-analysis	2023	
36	Exclude	wrong outcome	Preeclampsia: Recent Advances in Predicting, Preventing, and Managing the Maternal and Fetal Life-Threatening Condition	2023	
37	Exclude	wrong population	Prenatal predictor of composite adverse outcome in pregnancies undergoing induction of labour for fetal growth restriction	2023	
38	Exclude	wrong outcome	Real-time prediction of massive transfusion during cesarean section using intraoperative hemodynamic monitoring data	2023	
39	Exclude	wrong population	Relationship between perfusion index and central temperature before and after induction of anesthesia in laparoscopic gastrointestinal surgery: A prospective cohort study	2023	
40	Exclude	no details of PI or PVI	Role of perfusion index in early detection of hypotension following spinal anesthesia for cesarean section	2023	
41	Exclude	wrong outcome	Skin to origin of mesenteric artery study (STOMA): prospective study of distances between stoma location and proximal superior	2023	
42	Exclude	wrong outcome	Systemic opioid regimens for postoperative pain in neonates	2023	
43	Exclude	wrong outcome	The 74th Annual Congress of the Japan Society of Obstetrics and Gynecology	2023	
44	Exclude	wrong outcome	The cardiac-fetal-placental unit: fetal umbilical vein flow rate is linked to the maternal cardiac profile in fetal growth restriction	2023	
45	Exclude	wrong outcome	The End Is in Sight: Current Strategies for the Elimination of HIV Vertical Transmission	2023	
46	Exclude	wrong population	The role of the PLGF in the management of pregnancies complicated with fetal microsomia	2023	
47	Exclude	wrong outcome	The use of processed electroencephalography (pEEG) in obstetric anaesthesia: a narrative review	2023	
48	Exclude	wrong population	Totally percutaneous versus surgical cut-down femoral artery access for elective bifurcated abdominal endovascular aneurysm repair	2023	
49	Exclude	wrong outcome	Trends in Attempted and Successful Trial of Labor After Cesarean Delivery in the United States From 2010 to 2020	2023	

50	Exclude	wrong outcome	Uterine artery Doppler in early labor and perinatal outcome of low-risk term pregnancies: prospective multicenter study	2023	
51	Exclude	wrong outcome	a Comparison Between the Effects of Crystalloids and Colloids on Lung Ultrasound	2022	
52	Exclude	registration	A drug (ONDANSETRON), used in treatment of vomiting, has been shown to prevent fall in blood pressure after spinal anesthesia. This study identifies efficacy of ONDANSETRON in preventing fall in blood pressure after spinal anesthesia in pregnant women coming for	2022	
53	Exclude	wrong study design	A hepatitis B virus flare that led to hepatic decompensation and liver transplantation in a pregnant woman with chronic hepatitis B: a rare case report and literature review	2022	
54	Exclude	wrong study design	A High Risk Pregnancy with Thrombotic Thrombocytopenic Purpura and Hemolytic Uremic Syndrome Complicated by Acute Myocardial	2022	
55	Exclude	wrong outcome	A maternal higher-complex carbohydrate diet increases bifidobacteria and alters early life acquisition of the infant microbiome in women with gestational diabetes mellitus	2022	
56	Include		A study to evaluate perfusion index as a predictor of hypotension following spinal anesthesia for caesarean section	2022	https://doi.org/10.4103/joacp.JOACP_385_20
57	Exclude	registration	A study to predict hypotention during spinal anaesthesia in lower segment caesarean section by perfusion index	2022	
58	Exclude	wrong population	Accuracy and trending ability of finger plethysmographic cardiac output monitoring in late pregnancy	2022	
59	Exclude	wrong outcome	Added prognostic value of Doppler ultrasound for adverse perinatal outcomes: A pooled analysis of three cohort studies	2022	
60	Exclude	wrong outcome	Administration of antenatal corticosteroid is associated with reduced fetal growth velocity: a longitudinal study	2022	
61	Exclude	wrong study design	AhR/IL-22 pathway as new target for the treatment of post-infectious irritable bowel syndrome symptoms	2022	
62	Exclude	retracted	Analysis of Risk Factors for Intraoperative Hypotension in Cesarean Section and Poor Prognosis of Neonates	2022	
63	Exclude	wrong outcome	Analysis of the course and outcome of labor in patients who are at high risk according to prenatal screening results	2022	
64	Exclude	wrong outcome	Are children born by cesarean delivery at higher risk for respiratory sequelae?	2022	
65	Exclude	wrong outcome	Association between Low Birth Weight of the Infants and Maternal Periodontitis	2022	
66	Exclude	wrong outcome	Association between Pelvic Parameters and Vaginal Delivery	2022	
67	Exclude	wrong study design	Balancing Benefits and Risks of Indomethacin in the Management of Antenatal Bartter Syndrome: A Case Report	2022	

68	Exclude	wrong outcome	Birth Satisfaction During the Early Months of the COVID-19 Pandemic in the United States	2022	
69	Exclude	wrong outcome	Birth-Related Outcomes for Second Children Following Home Visiting Program Enrollment for New Parents of First Children	2022	
70	Exclude	wrong outcome	Body Mass Index and Mammographic Density in a Multiracial and Multiethnic Population-Based Study	2022	
71	Exclude	wrong study design	Case Report: Laparoscopic Isthmocele Repair on an 8 Weeks Pregnant Uterus	2022	
72	Exclude	wrong outcome	Cerebral perfusion changes of the basal ganglia and thalami in full-term neonates with hypoxic-ischaemic encephalopathy: a three-dimensional pseudo continuous arterial spin labelling perfusion magnetic resonance imaging study	2022	
73	Exclude	wrong outcome	Changes in preterm birth and caesarean deliveries in the United States during the SARS-CoV-2 pandemic	2022	
74	Exclude	wrong outcome	Color Doppler Sonography in fetal growth retardation: Need of the	2022	
75	Exclude	wrong population	Composite Score of Healthy Lifestyle Factors and the Risk of Pancreatic Cancer in a Prospective Cohort Study	2022	
76	Exclude	wrong outcome	Correlation between cerebroplacental doppler ratio and neonatal respiratory disorders: A reference marker of fetal lung maturation	2022	
77	Exclude	wrong outcome	Correlation between pleth variability index and ultrasonic inferior vena cava-collapsibility index in parturients with twin pregnancies undergoing cesarean section under spinal anesthesia	2022	
78	Exclude	wrong study design	Delivering siRNA Compounds During HOPE to Modulate Organ Function: A Proof-of-concept Study in a Rat Liver Transplant Model	2022	
79	Exclude	wrong outcome	Does ethnicity influence duration and rate of labour Australian population groups?	2022	
80	Exclude	wrong outcome	Does Insurance Status Effect the Nulliparous, Term, Singleton, Vertex Cesarean Delivery Rate?	2022	
81	Exclude	wrong outcome	Doppler Velocimetry of Umbilical Artery in Normal and Growth Restricted Pregnancy	2022	
82	Exclude	wrong outcome	Effect of Color Doppler Indices on the Diagnosis of Intrauterine Growth Restriction in High-Risk Pregnancies: A Cross-Sectional	2022	
83	Exclude	wrong outcome	Effect of Maternal and Fetal Characteristics in Feto-Placental Doppler and Impact of Using Adjusted Standards in the Definition of Fetal Growth Restriction at Term	2022	
84	Exclude	registration	Effects of different phenylephrine administration regimen on hemodynamics after combined spinal and epidural anesthesia in high-risk caesarean section patients with hypotension	2022	

85	Exclude	wrong outcome	Effects of Hypoplastic Fetal Umbilical Artery on Doppler Findings For Common Iliac Arteries	2022	
86	Exclude	wrong population	Energy expenditure and body composition in infants with bronchopulmonary dysplasia at term age	2022	
87	Exclude	wrong outcome	Enteral L Citrulline Supplementation in Preterm Infants - Safety, Efficacy and Dosing	2022	
88	Exclude	wrong population	Estimation of neonatal body fat percentage predicts neonatal hypothermia better than birthweight centile	2022	
89	Exclude	wrong study design	Estimation of the Exposure-Response Relation between Benzene and Acute Myeloid Leukemia by Combining Epidemiologic, Human Biomarker, and Animal Data	2022	
90	Exclude	wrong outcome	Evaluation of midtrimester ductus venosus diameter and peak systolic velocity to predict late onset small for gestational age fetuses	2022	
91	Exclude	wrong outcome	Fetal Biometry and Doppler Assessment of Pregnant Women with COVID-19	2022	
92	Exclude	wrong outcome	FGR: Risk Factors and Outcomes	2022	
93	Exclude	wrong outcome	Hazard and cumulative incidence of umbilical cord metabolic acidemia at birth in fetuses experiencing the second stage of labor and pathologic intrapartum fetal heart rate requiring expedited	2022	
94	Exclude	wrong outcome	Hemodynamic Changes in Umbilical Artery and Middle Cerebral Artery With Oligohydramnios in Third Trimester of Pregnancy	2022	
95	Exclude	wrong outcome	Identifying the Target Population for Primary Respiratory Syncytial Virus Two-Step Prevention in Infants: Normative Outcome of Hospitalisation Assessment for Newborns (NOHAN)	2022	
96	Exclude	wrong outcome	In utero exposure to antiretroviral drugs and pregnancy outcomes: Analysis of the French ANRS pharmacovigilance database	2022	
97	Exclude	wrong outcome	Incidence and determinants of maternal sepsis in Ghana in the midst of a pandemic	2022	
98	Exclude	wrong outcome	Intercristal line determined by palpation in parturients in the sitting and the lateral decubitus positions: a prospective observational study	2022	
99	Exclude	wrong population	Interventions for treating urinary incontinence in older women: a network meta-analysis	2022	
100	Exclude	wrong outcome	Intrapartum Sildenafil Citrate to Prevent Non Reassuring Foetal Status Among Parturients Delivering at Term	2022	
101	Exclude	wrong index test	Intrathecal dexamethasone for arterial hypotension management during spinal anaesthesia for elective caesarean section	2022	
102	Exclude	wrong outcome	Levonorgestrel intrauterine system for the treatment of intermenstrual spotting in patients with previous cesarean delivery scar defect	2022	

103	Exclude	wrong outcome	Longitudinal changes in fetal head biometry and fetoplacental circulation in fetuses with congenital heart defects	2022	
104	Exclude	wrong outcome	MAGnesium Effect With ANtiosteoporotic Drugs	2022	
105	Exclude	wrong population	Masks versus prongs as interfaces for nasal continuous positive airway pressure in preterm infants	2022	
106	Exclude	wrong outcome	Maternal and foetal physiological response of sacral surface electrical stimulation during pregnancy: A preliminary study	2022	
107	Exclude	wrong outcome	Maternal Morbidity After Double Balloon Catheter Management of Cesarean Scar and Cervical Pregnancies	2022	
108	Exclude	wrong outcome	Maternal postures for fetal malposition in labour for improving the health of mothers and their infants	2022	
109	Exclude	wrong outcome	Maternal Race/Ethnicity and Mode of Delivery in Twin Gestations	2022	
110	Exclude	wrong population	Menopausal status, ultrasound and biomarker tests in combination for the diagnosis of ovarian cancer in symptomatic women	2022	
111	Exclude	wrong outcome	Naldebain for Control of Post-Cesarean Section Pain	2022	
112	Exclude	wrong outcome	Negative pressure wound therapy for surgical wounds healing by primary closure	2022	
113	Exclude	wrong study design	Obstetric anesthesia: pearls and pitfalls in anesthesia for cesarean	2022	
114	Exclude	wrong outcome	Patterns of Brain Sparing in a Fetal Growth Restriction Cohort	2022	
115	Include		Perfusion Index and Its Correlation With Intraoperative Hypotension in Lower-Segment Cesarean Section Under Spinal Anesthesia: A Prospective Observational Study in a Tertiary Care Hospital in	2022	https://doi.org/10.7759/cureus.30431
116	Exclude	registration	perfusion index as screening tool in caesarean section	2022	
117	Exclude	registration	Perfusion Index vs Positional Hemodynamic Changes to Predict Hypotension After Spinal Anaesthesia in Caesarean Section	2022	
118	Exclude	wrong population	Perinatal arterial ischemic stroke and periventricular venous infarction in infants with unilateral cerebral palsy	2022	
119	Exclude	wrong outcome	Perinatal Outcome in Cases of Isolated Oligohydroamnios at Term at Tertiary Care Center of Wah Cantt & HIT Taxilla	2022	
120	Exclude	wrong study design	Peri-operative care of a rare case of Ogilvie's syndrome after lower segment caesarean section	2022	
121	Include		Peripheral Perfusion Index: A Predictor of Post-Spinal Hypotension in Caesarean Section	2022	https://doi.org/10.7759/cureus.25699
122	Exclude	wrong outcome	Persistence of abnormal uterine artery flow postpartum: Case-control	2022	
123	Exclude	wrong outcome	Pharmacological chaperones for the oxytocin receptor increase oxytocin responsiveness in myometrial cells	2022	
124	Exclude	wrong outcome	Physical activity and sedentary behaviours during pregnancy are associated with neonatal size at birth	2022	

125	Exclude	wrong population	Physiologic dysregulation in newborns with prenatal opioid exposure: Cardiac, respiratory and movement activity	2022	
126	Exclude	wrong population	Place and Pain: Association Between Neighborhood SES and Quantitative Sensory Testing Responses in Youth With Functional	2022	
127	Exclude	wrong outcome	Placental dysfunction influences fetal monocyte subpopulation gene expression in preterm birth	2022	
128	Exclude	wrong population	Placental vascular alterations are associated with early neurodevelopmental and pulmonary impairment in the rabbit fetal	2022	
129	Include		Pleth variability index measured in the sitting position before anesthesia can predict spinal anesthesia-induced hypotension in cesarean section: An observational study	2022	https://doi.org/10.4103/Joacc.Joacc_97_21
130	Exclude	wrong outcome	Postoperative comparison of SterizoneÂ® dressing versus standard gauze dressing in women having cesarean section	2022	
131	Exclude	wrong outcome	Predictive value of doppler cerebroplacental ratio for adverse perinatal outcomes in postdate pregnancies in Northwestern Nigeria	2022	
132	Exclude	wrong outcome	Preeclampsia prevention by timed birth at term	2022	
133	Exclude	wrong outcome	Preliminary Results on the Preinduction Cervix Status by Shear Wave Elastography	2022	
134	Exclude	wrong outcome	Preoperative perfusion index as a predictor of post-anaesthetic shivering in caesarean section with spinal anaesthesia	2022	
135	Exclude	registration	Prevention of spinal hypotension using peripheral perfusion index during caesarean section	2022	
136	Exclude	wrong outcome	Prophylactic cyclo-oxygenase inhibitor drugs for the prevention of morbidity and mortality in preterm infants: a network meta-analysis	2022	
137	Exclude	wrong population	Pulse perfusion index for predicting intrapartum fever during epidural analgesia	2022	
138	Exclude	wrong outcome	Relationship between fetal middle cerebral artery pulsatility index and cerebroplacental ratio with adverse neonatal outcomes in low-risk pregnancy candidates for elective cesarean section: A cross-sectional study	2022	
139	Exclude	wrong index test	Role of sFLT-1/PlGF ratio in predicting severe adverse materno-fetal outcome in high risk women	2022	
140	Exclude	wrong outcome	Role of umbilicocerebral and cerebroplacental ratios in prediction of perinatal outcome in FGR pregnancies	2022	
141	Exclude	wrong outcome	ROLE OF UTERINE ARTERY DOPPLER AT 11 TO 16 WEEKS OF GESTATION IN PREDICTION OF PREECLAMPSIA: AN OBSERVATIONAL STUDY	2022	

142	Exclude	wrong outcome	Second trimester uterine arteries pulsatility index is a function of placental pathology and provides insights on stillbirth aetiology: A multicenter matched case-control study	2022	
143	Exclude	wrong outcome	Serial Measurements of Fetal Head Circumference and Abdominal Circumference to Predict Fetal Growth Restriction in a Sri Lankan Study Population	2022	
144	Exclude	wrong indwx test	Strategic comparison of fluid management in pre-eclampsia before cesarean section between 1 ml/kg BW/hour of colloid plus 5 mg/hour of furosemide and 80 ml/hour of crystalloid: Hemodynamic optimalization and plasma albumin level improvements	2022	
145	Exclude	wrong outcome	The Application Value of Three-Dimensional Power Doppler Ultrasound in Fetal Growth Restriction	2022	
146	Exclude	wrong outcome	The association between fetal renal artery indices in late pregnancy and birth weight in gestational diabetes mellitus: A cohort study	2022	
147	Exclude	wrong outcome	The Delphi definition for selective fetal growth restriction may not improve detection of pathologic growth discordance in monochorionic	2022	
148	Exclude	wrong outcome	The effect of maternal intravenous hydration on amniotic fluid index in oligohydramnios	2022	
149	Exclude	wrong outcome	The effect of perioperative hypotension on perfusion index in infants in cesarean delivery with spinal anesthesia	2022	
150	Exclude	wrong outcome	The effects of a lifestyle intervention (the HealthyMoms app) during pregnancy on infant body composition: Secondary outcome analysis from a randomized controlled trial	2022	
151	Exclude	wrong outcome	The Kynurenine Pathway Metabolites in Cord Blood Positively Correlate With Early Childhood Adiposity	2022	
152	Exclude	wrong study design	The multidisciplinary management of a mechanical mitral valve thrombosis in pregnancy: a case report and review of the literature	2022	
153	Exclude	wrong outcome	The Prevalence, Incidence, Indications and Outcomes of Peripartum Hysterectomy in Kazakhstan: Data from Unified Nationwide Electronic Healthcare System 2014-2018	2022	
154	Exclude	wrong outcome	The Role of Transvaginal Two-Dimensional Ultrasound Combined With Color Doppler in the Evaluation of Ovarian Function and Fertility After Uterine Artery Embolization	2022	
155	Include		The Usefulness of Perfusion Index Derived from a Pulse Oximeter in Predicting Hypotension following Spinal Anesthesia for Cesarean	2022	https://doi.org/10.3126/nmcj.v24i4.50579
156	Exclude	wrong outcome	The Volume of T2 Low-Signal Band and the Width of the Widest Blood Vessel in Placenta Measured by MRI in Pregnant Women with Different Types of Placental Implantation and Its Differential Value	2022	

157	Exclude	wrong population	To find out the utility of novel formula for length of insertion of Umbilical catheter as compared to older Dunn nomogram	2022	
158	Include		To Study the Role of Perfusion Index as a Predictor of Hypotension during Spinal Anesthesia in Lower Segment Cesarean Section - A Prospective Observational Study	2022	https://doi.org/10.4103/aer.aer_50_21
159	Exclude	wrong outcome	Trans-cranial Doppler in prediction of adverse outcome in asphyxiated neonates	2022	
160	Exclude	wrong outcome	Treatment of periodontitis for glycaemic control in people with diabetes mellitus	2022	
161	Exclude	wrong outcome	Ultrasonic observation on hemodynamic parameters of uterine artery of patients with late-onset preeclampsia	2022	
162	Exclude	wrong outcome	Ultrasound Multiparametric Assessment of the Impact of Hypertensive Disorders of Pregnancy on Fetal Cardiac Function and Growth and Development	2022	
163	Exclude	wrong outcome	Ultrasound placental image texture analysis using artificial intelligence to predict hypertension in pregnancy	2022	
164	Exclude	wrong outcome	Ultrasound prediction of adverse perinatal outcome at diagnosis of late-onset fetal growth restriction	2022	
165	Exclude	wrong outcome	Uterine artery Doppler indices throughout gestation in women with and without previous Cesarean deliveries: a prospective longitudinal case-control study	2022	
166	Exclude	wrong outcome	Uterine vascularity in women with previous caesarean section and its potential role in implantation failure: a retrospective cohort study	2022	
167	Exclude	wrong outcome	Value of Cerebroplacental Ratio and Uterine Artery Doppler as Predictors of Adverse Perinatal Outcome in Very Small for Gestational Age at Term Fetuses	2022	
168	Exclude	wrong outcome	A Guided Mobile-Based Perinatal Mindfulness Intervention	2021	
169	Exclude	wrong population	A Study of Fetal Umbilical Artery Flows and Neonatal Outcome in Small for Gestational Age Fetus	2021	
170	Exclude	wrong outcome	Accurate neonatal heart rate monitoring using a new wireless, cap mounted device	2021	
171	Exclude	wrong outcome	Anaesthetic approach in pregnants infected with covid-19 in a tertiary maternity	2021	
172	Exclude	wrong outcome	Anaesthetic delivery in major abdominal surgery with an allergy to all neuromuscular bloking agent	2021	
173	Exclude	wrong population	Antibiotic regimens for early-onset neonatal sepsis	2021	
174	Exclude	wrong outcome	Aortic isthmus Doppler in normal and small-for-gestational-age fetuses and its association with prediction of adverse perinatal	2021	

175	Exclude	wrong outcome	Ascending Growth is Associated with Offspring Adiposity in Pregnancies Complicated with Obesity or Gestational Diabetes	2021	
176	Exclude	wrong outcome	Association between preoperative toe perfusion index and maternal core temperature decrease during cesarean delivery under spinal anesthesia: a prospective cohort study	2021	
177	Exclude	wrong outcome	Association between ultrasound markers of disproportionate fetal abdominal growth and shoulder dystocia	2021	
178	Exclude	wrong outcome	Association of birth mode of delivery with infant faecal microbiota, potential pathobionts, and short chain fatty acids: a longitudinal study over the first year of life	2021	
179	Exclude	wrong outcome	Association of parental incarceration and juvenile justice involvement with Pregnancy and neonatal outcomes	2021	
180	Exclude	wrong outcome	Associations between maternal circulating 25-hydroxyvitamin D concentration and birth outcomes-Mode of delivery and episiotomy rate: A prospective cohort study	2021	
181	Exclude	wrong outcome	Australian Society of Anaesthetists 78th National Scientific Congress, 20寰・4 September 2019, International Convention Centre, Sydney,	2021	
182	Exclude	wrong outcome	Birth by cesarean section and mood disorders among adolescents of a birth cohort study in northern Brazil	2021	
183	Include		Can a perfusion index, measured by pulse oximeter predict hypotension following spinal anesthesia in healthy parturients undergoing elective lower segment cesarean section?	2021	https://doi.org/10.33545/26643766.2021.v4.i1c.219
184	Exclude	wrong outcome	Cerebroplacental Ratio as a Predictor of Fetal Growth Restriction and Perinatal Outcome in Women with Hypertensive Disorder in Pregnancy: A Prospective Cohort Study	2021	
185	Exclude	wrong outcome	Cerebroplacental ratio in predicting adverse perinatal outcome: a meta-analysis of individual participant data	2021	
186	Exclude	wrong outcome	Cerebroplacental ratio in term pregnancy: A risk stratification tool in developing nations	2021	
187	Exclude	wrong outcome	Change in uterine artery blood flow with intrauterine balloon	2021	
188	Exclude	duplicate	Change in uterine artery blood flow with intrauterine balloon	2021	
189	Exclude	wrong outcome	Choosing the optimal skin incision for cesarean delivery in patients with morbid obesity	2021	
190	Exclude	review article	Clinical and Obstetric Aspects of Pregnant Women with COVID-19: A Systematic Review	2021	
191	Exclude	wrong outcome	Clinical monitoring of late fetal growth restriction	2021	
192	Exclude	wrong population	Clustering of Streptococcus gallolyticus subspecies pasteurianus bacteremia and meningitis in neonates	2021	

193	Exclude	wrong population	Continuous versus bolus intermittent intragastric tube feeding for preterm and low birth weight infants with gastro-oesophageal reflux	2021	
194	Exclude	wrong study design	Contrast-enhanced ultrasound evaluation of placental perfusion in brachicephalic bitches	2021	
195	Exclude	wrong population	Correlation of Fractional Limb Volume Measurements with Neonatal Morphometric Indices	2021	
196	Exclude	wrong outcome	Correlation of maternal body mass index with umbilical artery Doppler in pregnancies complicated by fetal growth restriction and associated outcomes	2021	
197	Exclude	wrong population	Delivery room ultrasound study to assess heart rate in newborns: DELIROUS study	2021	
198	Exclude	wrong outcome	Detection of Breathing Movements of Preterm Neonates by Recording Their Abdominal Movements with a Time-of-Flight	2021	
199	Exclude	wrong outcome	Development of a risk calculator for shoulder dystocia in patients with diabetes	2021	
200	Exclude	wrong study design	Diagnostic Complexities in a Case of Ovarian Hyperstimulation Syndrome Presented Solely as a Unilateral Massive Hydrothorax	2021	
201	Exclude	wrong population	Does Quantitative Sensory Testing Improve Prediction of Chronic Pain Trajectories? A Longitudinal Study of Youth With Functional Abdominal Pain Participating in a Randomized Controlled Trial of Cognitive Behavioral Treatment	2021	
202	Exclude	wrong outcome	Doppler ultrasound assessment of fetal mca and umbilical arteries in patients with gestational diabetes versus normal pregnancies	2021	
203	Exclude	wrong outcome	Duration Of Labour – Is it Influenced by Ethnicity?	2021	
204	Exclude	registration	Effect Of 3 different Doses Of Phenylephrine On Perfusion Index In Patients Undergoing Caesarean Section Under spinal anaesthesia	2021	
205	Exclude	wrong index test	EFFECT OF DIFFERENT NOREPINPHRINE APPLICATION METHODS IN CAESARIANES ON HYPOTENSION AFTER SPINAL	2021	
206	Exclude	registration	Effect of Phenyepherine and Nordrenaline infusion to predict low blood pressure in pregnant patients undergoing Caesarean section	2021	
207	Exclude	wrong outcome	Effectiveness of infant diet and physical activity modifications to prevent rapid weight gain in infants followed up at Teaching Hospital Peradeniya; A Randomized Control Trial	2021	
208	Exclude	wrong population	Enzyme replacement therapy with galsulfase for mucopolysaccharidosis type VI	2021	
209	Exclude	wrong outcome	Epidemiology of surgery in a protracted humanitarian setting: a 20-year retrospective study of Nyarugusu Refugee Camp, Kigoma, Western Tanzania	2021	

210	Exclude	wrong outcome	Evaluating the value of uterine artery doppler ultrasound indices in predicting fetal and neonatal complications in women with preeclampsia in Ahvaz, Iran	2021	
211	Exclude	wrong outcome	Experiences of Women Who Gave Birth in US Hospitals During the COVID-19 Pandemic	2021	
212	Exclude	wrong outcome	Feasibility of non-invasive cardiac output monitoring at birth using electrical bioreactance in term infants	2021	
213	Exclude	wrong population	Fetal brain hemodynamics in pregnancies at term: correlation with gestational age, birthweight and clinical outcome	2021	
214	Exclude	wrong outcome	Fetal left ventricular modified myocardial performance index and renal artery pulsatility index in pregnancies with isolated oligohydramnios before 37 weeks of gestation	2021	
215	Exclude	wrong outcome	First-trimester screening for fetal growth restriction using Doppler color flow analysis of the uterine artery and serum PAPP-A levels in unselected pregnancies	2021	
216	Exclude	wrong study design	Foetal ductus arteriosus constriction unrelated to non-steroidal anti-Inflammatory drugs: a case report and literature review	2021	
217	Exclude	wrong outcome	FOLFOX Therapy Induces Feedback Upregulation of CD44v6 through YB-1 to Maintain Stemness in Colon Initiating Cells	2021	
218	Exclude	wrong study design	Food protein-induced enterocolitis syndrome presenting after necrotizing enterocolitis in a preterm neonate: a case report	2021	
219	Exclude	wrong population	Formation of initial changes in hemodynamics and fluid compartments in high surgical risk patients under the influence of acute abdominal pathology	2021	
220	Exclude	wrong outcome	Growth Velocity and Doppler Evaluation to Predict Nonreassuring Fetal Heart Rate at Birth in Low-Risk Women: A Prospective,	2021	
221	Exclude	wrong outcome	Humanitarian surgical service utilization by a host country population: comparing surgery patterns between refugees and Tanzanians using an interrupted time-series analysis	2021	
222	Exclude	wrong outcome	Impact of Transversus Abdominis Plane Block With Bupivacaine or Ropivacaine Versus Intrathecal Morphine on Opioid-related Side Effects After Cesarean Delivery: A Meta-analysis of Randomized	2021	
223	Exclude	wrong population	Improved Survival of Periviable Infants after Alteration of the Threshold of Viability by the Neonatal Resuscitation Program 2015	2021	
224	Exclude	wrong outcome	Improving outcomes with revised preoperative universal decolonization protocol	2021	
225	Exclude	wrong study design	Increased end-expiratory pressures improve lung function in near-term newborn rabbits with elevated airway liquid volume at birth	2021	

226	Exclude	wrong population	Internet-delivered cognitive behavioral therapy for youth with functional abdominal pain: a randomized clinical trial testing differential efficacy by patient subgroup	2021	
227	Exclude	wrong outcome	Intrauterine resting tone: does obesity make a difference?	2021	
228	Exclude	wrong population	Intravenous NAC Use in ACLF Patients	2021	
229	Exclude	wrong population	Ketamine and other glutamate receptor modulators for depression in adults with bipolar disorder	2021	
230	Exclude	wrong population	Ketamine and other glutamate receptor modulators for depression in adults with unipolar major depressive disorder	2021	
231	Exclude	wrong outcome	Late (>/= 7 days) systemic postnatal corticosteroids for prevention of bronchopulmonary dysplasia in preterm infants	2021	
232	Exclude	wrong outcome	Leveraging the Massachusetts perinatal quality collaborative to address the COVID-19 pandemic among diverse populations	2021	
233	Exclude	wrong outcome	Low-dose oral misoprostol for induction of labour	2021	
234	Exclude	wrong study design	Maternal and fetal effects of COVID-19 virus on a complicated triplet pregnancy: a case report	2021	
235	Exclude	wrong outcome	Maternal and Neonatal Morbidity and Mortality Among Pregnant Women With and Without COVID-19 Infection: The INTERCOVID Multinational Cohort Study	2021	
236	Exclude	wrong population	Maternal and perinatal factors are associated with risk of pediatric central nervous system tumors and poorer survival after diagnosis	2021	
237	Exclude	wrong outcome	Maternal haemodynamics follow-up after pregnancies complicated by HDP and/or IUGR	2021	
238	Exclude	wrong outcome	Maternal toe perfusion index change after spinal anesthesia for cesarean delivery correlates with a decreased oxygen partial pressure of the umbilical vein	2021	
239	Exclude	wrong outcome	MCA/UtA ratio at first observation is associated with birthweight in late onset IUGR	2021	
240	Exclude	wrong outcome	Monitoring of gastric residual volume during enteral nutrition	2021	
241	Exclude	wrong outcome	Morphometric Analysis of Brain in Newborn with Congenital Diaphragmatic Hernia	2021	
242	Exclude	wrong outcome	Neoadjuvant chemotherapy before surgery versus surgery followed by chemotherapy for initial treatment in advanced ovarian epithelial	2021	
243	Exclude	wrong population	O82: Scorpion: Scoping Outcomes for Acute Pancreatitis: A Nationwide Study	2021	
244	Exclude	wrong outcome	Obesity, Second Stage Duration, and Labor Outcomes in Nulliparous Women	2021	

245	Exclude	wrong outcome	Optimum dose of spinal ropivacaine with or without single intravenous bolus of S-ketamine during elective cesarean delivery: a randomized, double-blind, sequential dose-finding study	2021	
246	Exclude	wrong outcome	Oral non-steroidal anti-inflammatory drugs (single dose) for perineal pain in the early postpartum period	2021	
247	Exclude	wrong outcome	Oxygen saturation and perfusion index screening in neonates at high altitudes: can PDA be predicted?	2021	
248	Exclude	wrong outcome	Paracetamol/acetaminophen (single administration) for perineal pain in the early postpartum period	2021	
249	Exclude	wrong outcome	Perinatal Outcome and its Prediction Using Longitudinal Feto-Maternal Doppler Follow-Up in Late Onset Small for Gestational Age Fetuses - A Prospective Cohort Study	2021	
250	Exclude	wrong outcome	Perinatal outcomes after intrauterine growth restriction and umbilical artery Doppler pulsatility index of less than the fifth percentile	2021	
251	Exclude	wrong outcome	Placental functional assessment and its relationship to adverse pregnancy outcome: comparison of intravoxel incoherent motion (IVIM) MRI, T2-relaxation time, and umbilical artery Doppler	2021	
252	Exclude	wrong outcome	Post-operative Patient Positioning Device for Improvement of Post-Operative Pain	2021	
253	Exclude	wrong outcome	Postpartum Ultrasound	2021	
254	Exclude	wrong outcome	Prediction of Perinatal Outcomes in Full-Term Fetuses Appropriates for Gestational Age by Evaluation of Cerebroplacental Ratio (Cpr) Before the Active Phase of Labor	2021	
255	Exclude	review article	Prediction of spinal anesthesia-induced hypotension during elective cesarean section: a systematic review of prospective observational	2021	
256	Exclude	wrong outcome	Pregnancy outcomes associated with an abnormal 50-g glucose screen during pregnancy: a systematic review and Meta-analysis	2021	
257	Exclude	wrong outcome	Prenatal prediction of fetal lung maturity by measuring fetal pulmonary artery doppler indices	2021	
258	Exclude	wrong outcome	Prophylactic postoperative antibiotics after emergent cesarean delivery and risk of postpartum infection or wound complication	2021	
259	Exclude	wrong outcome	Prophylactic postoperative antibiotics in emergent cesarean deliveries and risk of postpartum infection	2021	
260	Exclude	wrong population	Pulmonary Outcomes in Children Born Extremely and Very Preterm at 11 Years of Age	2021	
261	Exclude	wrong outcome	Push versus gravity for intermittent bolus gavage tube feeding of preterm and low birth weight infants	2021	
262	Exclude	wrong outcome	Racial/Ethnic Variations in Trial of Labor After Cesarean in an Understudied Population [37Q]	2021	

263	Exclude	wrong population	Randomized, Open-Label, Non-Inferiority, Phase 3 Study of Subcutaneous (SC) Versus Intravenous (IV) Daratumumab (DARA) Administration in Patients (Pts) with Relapsed or Refractory Multiple Myeloma (RRMM): Body Weight Subgroup Analysis of Columba	2021	
264	Exclude	wrong population	Randomized, Open-Label, Non-Inferiority, Phase 3 Study of Subcutaneous (SC) Versus Intravenous (IV) Daratumumab (DARA) Administration in Patients with Relapsed or Refractory Multiple	2021	
265	Exclude	wrong outcome	Reducing operative birth for fetal distress in women with small or suboptimally grown infants - The RidStress 2 Randomised Controlled	2021	
266	Exclude	wrong outcome	Relation Between Ovarian Doppler Study at the Day of Ova Pickup and Icsi Cycle Success	2021	
267	Exclude	wrong population	Relationship between Pleth Variability Index and the Occurrence of Hypotension during Epidural Anesthesia for Cesarean Section	2021	
268	Exclude	wrong study design	Resuscitation on haemorrhagic shock through unrecognised inadvertent brachial artery cannulation: A case report	2021	
269	Exclude	wrong outcome	Risk assessment of shoulder dystocia via the difference between transverse abdominal and biparietal diameters: A retrospective observational cohort study	2021	
270	Exclude	wrong outcome	Risk factors for massive hemorrhage during the treatment of cesarean scar pregnancy: a systematic review and meta-analysis	2021	
271	Exclude	duplicate	Scorpion: Scoping Outcomes for Acute Pancreatitis: A Nationwide	2021	
272	Exclude	wrong outcome	Spontaneous vaginal birth varies significantly across US hospitals	2021	
273	Exclude	wrong study design	Strategies for intra-amniotic administration of fetal therapy in a rabbit model of intrauterine growth restriction	2021	
274	Exclude	wrong outcome	Conference Abstract (The 73rd Annual Congress of the Japan Society of Obstetrics and Gynecology)	2021	
275	Exclude	wrong outcome	Conference Abstract (The 73rd Annual Congress of the Japan Society of Obstetrics and Gynecology)	2021	
276	Exclude	wrong outcome	The diagnostic value of ultrasound detection of the fetal middle cerebral artery, umbilical artery blood flow and fetal movement reduction in fetal distress	2021	
277	Exclude	wrong outcome	The effect of nuchal umbilical cord on fetal cardiac and cerebral circulation-cross-sectional study	2021	
278	Exclude	wrong index test	The Effectiveness Of Non Invasive Hemodynamic Parameters In Detection Of Spinal Anesthesia Induced Hypotension During	2021	
279	Exclude	wrong outcome	The Role of Surgical Attire in the Prevention of Postoperative Infections: A Before and After Study [24T]	2021	
280	Exclude	wrong population	Tracheal suction at birth in non-vigorous neonates born through meconium-stained amniotic fluid	2021	

281	Exclude	wrong outcome	Transdermal delivery of flurbiprofen from polyoxypropylene-polyoxyethylene block copolymer stabilized reduced graphene oxide to manage pain in spondylitis: In vitro and in vivo studies	2021	
282	Exclude	wrong outcome	Ultrasound for diagnosis of birth weight discordance in twin	2021	
283	Exclude	wrong population	Umbilical cord blood hematological parameters reference interval for newborns from Addis Ababa, Ethiopia	2021	
284	Exclude	wrong outcome	Umbilical vein injection for management of retained placenta	2021	
285	Exclude	wrong population	Updated Efficacy and Safety from a Phase 1/2 Study of Melflufen and Dexamethasone Plus Bortezomib or Daratumumab in Patients with Relapsed/Refractory Multiple Myeloma (RRMM) Refractory to an IMiD or a Proteasome Inhibitor (PI)	2021	
286	Exclude	wrong outcome	Utero-placental and cerebrovascular indices in pregnant women with systemic lupus erythematosus: Relation to disease activity and pregnancy outcome	2021	
287	Exclude	wrong outcome	Which is the best ultrasound parameter for the prediction of adverse perinatal outcome within 1 day of delivery?	2021	
288	Include		A BASELINE PERFUSION INDEX AS A PREDICTOR OF HYPOTENSION FOLLOWING SUBARACHNOID BLOCK IN LOWER SEGMENT CAESAREAN SECTION-A PROSPECTIVE OBSERVATIONAL STUDY – A STUDY OF 60 CASES	2020	http://repository-tnmgrmu.ac.in/13731/1/201008720steffi.pdf (Accessed June 23, 2023).
289	Exclude	registration	A clinical study on the effect of giving a left sided tilt to the pregnant uterus on the blood flow in lower limbs of pregnant women undergoing caesarean section under spinal anaesthesia	2020	
290	Exclude	wrong outcome	A comparative study between dexmedetomidine and dexamethasone as an intrathecal adjuvant for prevention of perioperative shivering in cesarean section	2020	
291	Exclude	wrong population	A digital health psychological intervention (WebMAP Mobile) for children and adolescents with chronic pain: results of a hybrid effectiveness-implementation stepped-wedge cluster randomized trial	2020	
292	Exclude	wrong outcome	A randomized trial of the bactericidal effects of chlorhexidine vs. povidone iodine vaginal preparation	2020	
293	Include		A study of baseline perfusion index using pulse oximeter as a non invasive predictor of hypotension following spinal anaesthesia in lower segment caesarean section	2020	https://journals.lww.com/ijaweb/fulltext/2020/64001/tn_jha_and_kp_chansoria_travel_grant_award.131.aspx
294	Exclude	duplicate	A study to evaluate perfusion index as a predictor of hypotension following spinal anaesthesia for caesarean section	2020	

295	Exclude	registration	Accuracy and trending abilities of finger plethysmographic blood pressure and cardiac output compared to invasive measurements during caesarean delivery in healthy women: an observational study	2020	
296	Exclude	wrong outcome	Assessment of Thyroid Function by Term and Gestational Age: A Retrospective Analysis Using the Ponderal Index	2020	
297	Exclude	wrong outcome	Association of objectively measured physical fitness during pregnancy with maternal and neonatal outcomes. The GESTAFIT	2020	
298	Exclude	wrong outcome	Associations Among Intrapartum Interventions and Cesarean Birth in Low-Risk Nulliparous Women with Spontaneous Onset of Labor	2020	
299	Exclude	wrong outcome	Blood Glucose and Lactate Levels and Cerebral Oxygenation in Preterm and Term Neonates-A Systematic Qualitative Review of the	2020	
300	Exclude	wrong study design	Breast reconstruction with superior epigastric artery perforator (SEAP) free flap: Report of two cases	2020	
301	Exclude	wrong outcome	Can sonographic assessment of pulmonary vascular reactivity following maternal hyperoxygenation predict neonatal pulmonary hypertension? (HOTPOT study protocol)	2020	
302	Exclude	wrong outcome	Changes in choroidal circulation and pulse waveform in a case of pregnancy-induced hypertension with serous retinal detachment	2020	
303	Exclude	wrong outcome	Changes in Umbilical Artery Doppler Velocimetry After Betamethasone Administration in Pregnancies With Fetal Growth	2020	
304	Exclude	wrong outcome	Clinical efficacy of lower abdominal transcutaneous electrical stimulation combined with mifepristone plus methyl carboprost suppository in the treatment of missed abortion and its effects on endometrial thickness, hemodynamics, serum hypoxia inducible factor 1ﾎｱ and vascular endothelial growth fa...	2020	
305	Exclude	wrong population	Comparing the complications between open and keyhole	2020	
306	Exclude	wrong population	Comparison of a respiratory effort signal derived from photoplethysmography against polysomnography thoracic and abdominal belts in participants with sleep-disordered breathing	2020	
307	Exclude	wrong outcome	Comparison of effect of oral Utrogestan and intramascular Proluton on Maternal and fetal Doppler circulatuion in women with preterm	2020	
308	Exclude	wrong outcome	Conceptual model for pluralistic healthcare behaviour: results from a qualitative study in southwestern Uganda	2020	
309	Exclude	wrong outcome	Contrast-Enhanced Ultrasound Evaluation of Mifepristone for Treatment of Low-Risk Cesarean Scar Pregnancy	2020	
310	Exclude	wrong outcome	Cost of Elective Labor Induction Compared With Expectant Management in Nulliparous Women	2020	
311	Exclude	wrong outcome	Coverage, associated factors, and impact of companionship during labor: A large-scale observational study in six hospitals in Nepal	2020	

312	Exclude	wrong population	Delivering Clinical impacts of the MRI diagnostic pathway in prostate cancer diagnosis	2020	
313	Exclude	wrong outcome	Development and Optimization of Rizatriptan Benzoate Ethosomes	2020	
314	Exclude	wrong outcome	Development and Validation of a Risk Prediction Model for Cesarean Delivery After Labor Induction	2020	
315	Exclude	wrong outcome	Diagnostic Category Prevalence in 3 Classification Systems Across the Transition to the International Classification of Diseases, Tenth Revision, Clinical Modification	2020	
316	Exclude	wrong outcome	Does birth mode modify associations of maternal pre-pregnancy BMI and gestational weight gain with the infant gut microbiome?	2020	
317	Exclude	wrong outcome	Does Uterine Doppler Add Information to the Cerebroplacental Ratio for the Prediction of Adverse Perinatal Outcome at the End of	2020	
318	Exclude	review article	Dyadic Psychosocial eHealth Interventions: Systematic Scoping	2020	
319	Exclude	wrong population	Early full enteral feeding for preterm or low birth weight infants	2020	
320	Exclude	wrong outcome	Early gestational diabetes screening in obese women: a randomized controlled trial	2020	
321	Exclude	wrong outcome	Effect of previous cesarean sections on second-trimester uterine artery Doppler	2020	
322	Exclude	wrong population	EFFECT OF RESPIRATORY SUPPORT ON LUNGFUNCTION IN NEWBORNS WITH ELEVATED AIRWAYLIQUID VOLUME AT BIRTH; TOWARDS IMPROVEDUNDERSTANDING AND MANAGEMENT OF NEAR-TERM RESPIRATORY DISTRESS	2020	
323	Exclude	wrong outcome	Effect of sildenafil on maternal hemodynamics in pregnancies complicated by severe early-onset fetal growth restriction: planned subgroup analysis from a multicenter randomized placebo-controlled	2020	
324	Exclude	wrong outcome	Factors associated with neonatal near miss in Brazil	2020	
325	Exclude	wrong outcome	Fetal Distribution of Feto-placental Blood Flow Related to Placental Nutrient Transport and Maternal Food Intake	2020	
326	Exclude	wrong outcome	First trimester biomarkers for prediction of gestational diabetes	2020	
327	Exclude	wrong outcome	First-trimester placental function in levothyroxine-using pregnant women: a case-control study	2020	
328	Exclude	wrong outcome	Gestational Age of Delivery in Pregnancies Complicated by Diabetes	2020	
329	Exclude	wrong population	Goal-directed therapy with bolus albumin 5% is not superior to bolus ringer acetate in maintaining systemic and mesenteric oxygen delivery in major upper abdominal surgery: A randomised controlled	2020	
330	Exclude	wrong outcome	Immunity Modification of Full Term Infants According to the Type of Feeding and Mode of Delivery	2020	
331	Exclude	wrong study design	Improving lung aeration in ventilated newborn preterm rabbits with a partially aerated lung	2020	

332	Exclude	wrong outcome	Increased Visceral Adipose Tissue Without Weight Retention at 59 Weeks Postpartum	2020	
333	Exclude	wrong outcome	Individualized PEEP to optimise respiratory mechanics during abdominal surgery: a pilot randomised controlled trial	2020	
334	Exclude	wrong outcome	Inhibition of antigen-specific immune responses by co-application of an indoleamine 2,3-dioxygenase (IDO)-encoding vector requires antigen transgene expression focused on dendritic cells	2020	
335	Exclude	wrong outcome	Interaction between Maternal Immune Activation and Antibiotic Use during Pregnancy and Child Risk of Autism Spectrum Disorder	2020	
336	Exclude	wrong population	Interventions for American cutaneous and mucocutaneous	2020	
337	Exclude	wrong outcome	Interventions to prevent women from developing gestational diabetes mellitus: an overview of Cochrane Reviews	2020	
338	Exclude	wrong outcome	Intrauterine growth restriction: more than just low birthweight; introducing a new method of quantitating growth asymmetry	2020	
339	Exclude	wrong outcome	Intrauterine Hypoxia Induces Compensatory Changes in Fetal Heart Function in Growth Restricted Fetuses in a Sex Dependent Manner	2020	
340	Exclude	wrong population	Is Low Level Laser Therapy (LLLT) Effective in Reducing Pain Experienced by Women With Provoked Vestibulodynia?	2020	
341	Exclude	wrong study design	Lack of Evidence for Microbiota in the Placental and Fetal Tissues of Rhesus Macaques	2020	
342	Exclude	wrong outcome	Melatonin Levels Decrease in the Umbilical Cord in Case of Intrauterine Growth Restriction	2020	
343	Exclude	wrong outcome	Membrane sweeping for induction of labour	2020	
344	Exclude	wrong outcome	Middle cerebral artery Doppler pulsatility index as a predictor of intrapartum meconium release in prolonged pregnancies	2020	
345	Exclude	wrong index test	Norepinephrine as an Alternative in Hypotension Prophylaxis During Cesarean Section under Spinal Anesthesia	2020	
346	Exclude	wrong outcome	Normative references and clinical correlates of fetal umbilical artery Doppler indices in southwestern Nigeria	2020	
347	Exclude	wrong index test	Observation of hemodynamic parameters using a non-invasive cardiac output monitor system to identify predictive indicators for post-spinal anesthesia hypotension in parturients undergoing	2020	
348	Exclude	wrong outcome	Outcomes associated with an abnormal 50-gram glucose screen during pregnancy: A systematic review and meta-analysis	2020	
349	Exclude	wrong population	Palbociclib and Trastuzumab in HER2-Positive Advanced Breast Cancer: Results from the Phase II SOLTI-1303 PATRICIA Trial	2020	
350	Exclude	wrong population	Pancreatic enzyme replacement therapy for people with cystic	2020	
351	Exclude	wrong population	Parental adiposity differentially associates with newborn body	2020	

352	Exclude	wrong outcome	Pathologically diagnosed superficial form of placenta accreta: a comparative analysis with invasive form and asymptomatic muscular adhesion	2020	
353	Exclude	wrong outcome	Perfusion index as an early predictor of hypotension following spinal anesthesia for cesarean section	2020	
354	Exclude	registration	PERFUSION INDEX IN PREDICTING INTRAOPERATIVE HYPOTENSION IN PATEINTS UNDERGOING CAESAREAN SECTION UNDER SPINAL ANAESTHESIA	2020	
355	Exclude	wrong outcome	Perinatal outcomes from preterm and early term births in a multicenter cohort of low risk nulliparous women	2020	
356	Exclude	wrong population	Pictorial calendar for adherence to complementary feeding (PiC-	2020	
357	Exclude	wrong outcome	PIH4 Preoperative Skin Preparation for Caesarean Deliveries in Australian Public Hospitals: Surgical Site Infecton and Cost Outcomes Associated with Using Alcoholic Chlorhexidine Gluconate Applicator Versus Bulk Aqueous Povidone Iodine Solution	2020	
358	Exclude	wrong outcome	Pilot Testing a Mobile App to Designed to Increase Physical Activity Among Pregnant and Postpartum Women	2020	
359	Exclude	wrong outcome	Pioglitazone for prevention or delay of type 2 diabetes mellitus and its associated complications in people at risk for the development of type 2 diabetes mellitus	2020	
360	Exclude	wrong outcome	Prediction Model for Vaginal Birth After Induction of Labor in Women With Hypertensive Disorders of Pregnancy	2020	
361	Include		Prediction of Post spinal anesthesia hypotension in patients undergoing cesarean section using Perfusion index	2020	https://doi.org/10.3126/bjhs.v5i3.33708
362	Exclude	wrong outcome	Pregnancy and neonatal outcomes in women with HIV-1 exposed to integrase inhibitors, protease inhibitors and non-nucleoside reverse transcriptase inhibitors: an observational study	2020	
363	Exclude	wrong outcome	Prenatal test predicting respiratory morbidity at birth among growth restricted infants: a prospective observational study	2020	
364	Exclude	wrong outcome	Preoperative Antisepsis with Chlorhexidine Versus Povidone-Iodine for the Prevention of Surgical Site Infection: a Systematic Review and Meta-analysis	2020	
365	Exclude	wrong outcome	Prevalence of calcium and vitamin D deficiency and their association with feto-maternal outcomes in a sample of Iranian pregnant women	2020	
366	Exclude	wrong outcome	Printed educational materials: effects on professional practice and healthcare outcomes	2020	
367	Exclude	wrong outcome	Prolonged postpartum length of hospital stay among women with	2020	
368	Exclude	wrong outcome	Reduced growth velocity from the mid-trimester is associated with placental insufficiency in fetuses born at a normal birthweight	2020	

369	Exclude	wrong outcome	Reduction in racial disparities in severe maternal morbidity from hemorrhage in a large-scale quality improvement collaborative	2020	
370	Exclude	wrong outcome	Renal resistance indices and neutrophil gelatinase-associated lipocalin for early prediction of acute kidney injury in patients with	2020	
371	Exclude	wrong outcome	Retrospective Analysis of Uterine Artery Embolization in Cases of Placenta Increta or Percreta Undergoing Hysterectomy.	2020	
372	Exclude	wrong outcome	Role of Doppler ultrasound at time of diagnosis of late-onset fetal growth restriction in predicting adverse perinatal outcome: prospective cohort study	2020	
373	Exclude	wrong outcome	Safety and efficacy of sildenafil citrate to reduce operative birth for intrapartum fetal compromise at term: a phase 2 randomized	2020	
374	Exclude	wrong outcome	Second generation of the 1993 birth cohort, Pelotas (Brazil): Aims, design, preliminary results	2020	
375	Exclude	wrong outcome	Self-reported periconception weight loss attempts do not alter infant body composition	2020	
376	Exclude	wrong outcome	Seroprevalence analysis of SARS-CoV-2 in pregnant women along the first pandemic outbreak and perinatal outcome	2020	
377	Exclude	wrong outcome	Stem cell-based interventions for the prevention of morbidity and mortality following hypoxic-ischaemic encephalopathy in newborn	2020	
378	Exclude	wrong outcome	Conference Abstract (The 72nd Annual Congress of the Japan Society of Obstetrics and Gynecology)	2020	
379	Exclude	wrong outcome	The Association Between Pelvic Floor Muscle Force and General Strength and Fitness in Postpartum Women	2020	
380	Exclude	wrong outcome	TuMMI Trial: temperature treatment of Milk impacts on MRI digestion rates and nutrient delivery	2020	
381	Exclude	wrong outcome	Ultrasound markers for prediction of complex gastroschisis and adverse outcome: longitudinal prospective nationwide cohort study	2020	
382	Exclude	wrong outcome	Uterotonic agents for first-line treatment of postpartum haemorrhage: a network meta-analysis	2020	
383	Exclude	wrong outcome	Utility of ultrasound assessment in management of pregnancies with preterm prelabor rupture of membranes	2020	
384	Exclude	wrong outcome	Variation of intrapartum care and cesarean rates among practitioners attending births of low-risk, nulliparous women	2020	
385	Exclude	wrong outcome	Volume blood flow-based indices of fetal brain sparing in the second half of pregnancy: A longitudinal study	2020	
386	Exclude	wrong outcome	Limited Added Value of Cerebroplacental Ratio in Predicting Adverse Perinatal Outcomes: A Meta-Analysis on Individual	2019	
387	Exclude	wrong study design	Longitudinal Effects of Preterm Birth on patterns of Respiration in Infant Pigs	2019	

388	Exclude	wrong study design	Longitudinal Effects of RLN Lesion of Rates of Swallowing and Respiration in Infant Pigs	2019	
389	Exclude	wrong outcome	[Effect of auricular acupuncture on postpartum rehabilitation of primipara with cesarean]	2019	
390	Exclude	wrong outcome	[Risk factors and sonographic findings associated with the type of placenta accreta spectrum disorders]	2019	
391	Exclude	wrong outcome	Added value of fetal grouth velocity and Dopplers to biometry in identifying small-for-gestational-age fetuses with adverse perinatal outcome at term: prediction model	2019	
392	Exclude	wrong study design	Altered fetal hemodynamic status and neuroinflammation in a nonhuman primate model of intrauterine Ureaplasma parvum	2019	
393	Exclude	wrong population	An anticancer effect of umbilical cord-derived mesenchymal stem cell secretome on the breast cancer cell line	2019	
394	Exclude	wrong study design	Anesthetic management in a spinal cord-injured parturient woman with a left hip resection and secondary scoliosis: A case report	2019	
395	Exclude	wrong outcome	Anthropometric reference curves for term neonates born at 3400 meters above sea level	2019	
396	Exclude	wrong outcome	Are overweight and obesity associated with increased risk of cesarean delivery in Mexico? A cross-sectional study from the National Survey of Health and Nutrition	2019	
397	Exclude	wrong outcome	Arterial Pulse Waves between Bladder Wall and Uterine Serosa Are Absent in Normal Placenta, and When Present, Predictive of Ppercreta: A Case Control Study.	2019	
398	Exclude	wrong outcome	Association between hysteroscopic findings of previous cesarean delivery scar defects and abnormal uterine bleeding	2019	
399	Exclude	wrong outcome	Association of sedentary time and physical activity during pregnancy with maternal and neonatal birth outcomes. The GESTAFIT Project	2019	
400	Exclude	wrong outcome	Associations among cervical dilatation at admission, intrapartum care, and birth mode in low-risk, nulliparous women	2019	
401	Exclude	wrong outcome	Biomarkers of impaired placentation at 35-37 weeks' gestation in the prediction of adverse perinatal outcome	2019	
402	Include		Can Perfusion Index or Pleth Variability Index Predict Spinal Anesthesia Induced Hypotension During Caesarean Section?	2019	https://doi.org/10.5222/jarss.2019.69775
403	Exclude	wrong outcome	Can Placental Inositol (PI) Suppress the Pro-Adipogenic Effects of Maternal Glycaemia in the Fetus?	2019	
404	Exclude	wrong outcome	Changes in Placental Lipidomics with Obesity and Gestational Diabetes: Sexual Dimorphism	2019	

405	Exclude	wrong population	Combined proximal descending aortic endografting plus distal bare metal stenting (PETTICOAT technique) versus conventional proximal descending aortic stent graft repair for complicated type B aortic	2019	
406	Exclude	wrong population	Continuous local anaesthetic wound infusion for postoperative pain after midline laparotomy for colorectal resection in adults	2019	
407	Exclude	wrong population	Contrast-enhanced bowel ultrasound in patients with ulcerative colitis	2019	
408	Exclude	wrong study design	Coordination between respirarion and swallowing in infant pigs with and without a recuurent lalyngeal nerve lesion	2019	
409	Exclude	wrong outcome	Correlation of Fetal Doppler Assessments with Neonatal Ponderal Index in Fetal Growth Restriction.	2019	
410	Exclude	wrong outcome	Correlation of short-term variation and Doppler parameters with adverse perinatal outcome in low-risk fetuses at term	2019	
411	Exclude	wrong outcome	Correlation of short-term variation and Doppler parameters with adverse perinatal outcome in small-for-gestational age fetuses at	2019	
412	Exclude	wrong outcome	Does bilateral uterine artery ligation have negative effects on ovarian reserve markers and ovarian artery blood flow in women with postpartum hemorrhage?	2019	
413	Exclude	wrong outcome	Does exercise during pregnancy impact on maternal weight gain and fetal cardiac function? A randomized controlled trial	2019	
414	Exclude	wrong population	Effect of High-Flow Nasal Cannula on Thoraco-Abdominal Synchrony in Pediatric Subjects After Cardiac Surgery	2019	
415	Exclude	wrong outcome	Effect of maternal hypertension on preterm infant body composition at discharge	2019	
416	Exclude	wrong outcome	Efficacy and safety of the randomized, open-label, non-inferiority, phase 3 study of subcutaneous (SC) versus intravenous (IV) daratumumab (DARA) administration in patients (pts) with relapsed or refractory multiple myeloma (RRMM): COLUMBA	2019	
417	Exclude	wrong population	Enzyme replacement therapy with laronidase (Aldurazyme((R))) for treating mucopolysaccharidosis type I	2019	
418	Exclude	wrong outcome	Evaluation of Vascularity and Colour Doppler Blood Flow in Uterine Myometrium after Delivery	2019	
419	Exclude	wrong outcome	Failure of Decidualization and Maternal Immune Tolerance Underlies Uterovascular Resistance in Intra Uterine Growth Restriction	2019	
420	Exclude	wrong outcome	Failure of decidualization and maternal immune tolerance underlies uterovascular resistance in severe intra uterine growth restriction	2019	
421	Exclude	wrong outcome	Fetal movement monitoring in normal and high-risk pregnancy	2019	
422	Exclude	wrong population	Hand Forceps vs. Conventional One-hand Technique for Fetal Head Extraction During Cesarean Section	2019	

423	Exclude	wrong outcome	Hemodynamic factors associated with fetal cardiac remodeling in late fetal growth restriction: a prospective study	2019	
424	Exclude	wrong outcome	How Should Social Media Be Used in Transplantation? A Survey of the American Society of Transplant Surgeons	2019	
425	Exclude	wrong population	Human albumin infusion for treating oedema in people with nephrotic syndrome	2019	
426	Exclude	wrong population	Iatrogenic endometriosis harbors somatic cancer-driver mutations	2019	
427	Exclude	wrong population	Impact on treatment safety and efficacy of seromuscular-layer injury after HIFU ablation for uterine fibroids	2019	
428	Exclude	wrong population	Improving nutrition for critically ill hemodynamically unstable postcardiac surgery patients	2019	
429	Exclude	wrong outcome	Incidence of maternal peripartum infection: A systematic review and meta-analysis	2019	
430	Exclude	wrong outcome	Influence of maternal obesity and metabolic and vascular mediators in twin-twin transfusion syndrome	2019	
431	Exclude	wrong outcome	Isolated Placental Inflammation and Vasculopathy: Clinical Implications in the Extremely Low Birth Weight Infants	2019	
432	Exclude	wrong outcome	Labor Curve Analysis of Medically Indicated Early Preterm Induction of Labor	2019	
433	Exclude	review article	Labour pain control by aromatherapy: A meta-analysis of randomised controlled trials	2019	
434	Exclude	wrong outcome	Livedoid vasculopaty and anesthetic management in cesarean	2019	
435	Exclude	wrong outcome	Liver Enzyme Elevation in Pregnant Women Receiving Antiretroviral Therapy in the ANRS-French Perinatal Cohort	2019	
436	Exclude	wrong outcome	Magnesium for treating sickle cell disease	2019	
437	Exclude	wrong outcome	Maternal Fontan procedure is a predictor of a small-for-gestational-age neonate: a 10-year retrospective study	2019	
438	Exclude	wrong population	Modifications of Own Mothers' Milk Fortification Protocol Affect Early Plasma IGF-I and Ghrelin Levels in Preterm Infants. A Randomized Clinical Trial	2019	
439	Exclude	wrong population	Monitoring of plethysmography variability index and total hemoglobin levels during cesarean sections with antepartum hemorrhage for early detection of bleeding	2019	
440	Exclude	wrong population	Non-invasive positive pressure ventilation (CPAP or bilevel NPPV) for cardiogenic pulmonary oedema	2019	
441	Exclude	wrong study design	Perinatal Breathing Patterns and Survival in Mice Born Prematurely and at Term	2019	
442	Exclude	wrong outcome	Perinatal Risk Factors and Outcome Coding in Clinical and Administrative Databases	2019	

443	Exclude	wrong index test	Perioperative noninvasive cardiac output monitoring in parturients undergoing cesarean delivery with spinal anesthesia and prophylactic phenylephrine drip: a prospective observational cohort study	2019	
444	Exclude	wrong outcome	Perioperative restrictive versus goal-directed fluid therapy for adults undergoing major non-cardiac surgery	2019	
445	Exclude	wrong outcome	Personalized viral genomic investigation of herpes simplex virus 1 perinatal viremic transmission with dual fatality	2019	
446	Exclude	wrong outcome	Prediction of adverse perinatal outcome by cerebroplacental ratio in women undergoing induction of labor	2019	
447	Exclude	wrong outcome	Prediction of adverse perinatal outcome by serum placental growth factor and soluble fms-like tyrosine kinase-1 in women undergoing induction of labor	2019	
448	Exclude	registration	Prediction of hypotension after spinal anaesthesia in caesarean	2019	
449	Exclude	wrong outcome	Prediction of small for gestational age neonates: screening by maternal factors, fetal biometry, and biomarkers at 35-37 weeks'	2019	
450	Exclude	wrong outcome	Preliminary report on the use of a levonorgestrel intrauterine system for the treatment of intermenstrual bleeding due to previous cesarean delivery scar defect	2019	
451	Exclude	wrong outcome	Prenatal detection and obstetric management of true umbilical cord knots using color Doppler and 4D ultrasonography: a report of two cases and literature review	2019	
452	Exclude	wrong outcome	Prognostic value of the aortic isthmus Doppler assessment on late onset fetal growth restriction	2019	
453	Exclude	wrong population	Prostacyclin for pulmonary arterial hypertension	2019	
454	Exclude	wrong outcome	Prostanoids and their analogues for the treatment of pulmonary hypertension in neonates	2019	
455	Exclude	wrong population	Psychological interventions for parents of children and adolescents with chronic illness	2019	
456	Exclude	wrong population	Psychological therapies (remotely delivered) for the management of chronic and recurrent pain in children and adolescents	2019	
457	Exclude	wrong study design	Quantifying subclinical trauma associated with calving difficulty, vigour, and passive immunity in newborn beef calves	2019	
458	Exclude	wrong population	Rec-LH PD and Safety Profile in Hypogonadotropic Hypogonadism	2019	
459	Exclude	wrong study design	Restriction of early fetal growth and placental chorioangiomatosis	2019	
460	Exclude	wrong outcome	Singing and COPD: a pilot randomised controlled trial	2019	
461	Exclude	wrong outcome	Skin-to-skin contact during caesarean delivery: An intriguing interaction between the mother and her child	2019	
462	Exclude	wrong study design	Small for gestational age newborns in Cantonal hospital Zenica in the 6 months period a retrospective case series study	2019	

463	Exclude	wrong population	Stopping enteral feeds for prevention of transfusion-associated necrotising enterocolitis in preterm infants	2019	
464	Exclude	wrong study design	Structural plasticity and isolation of umbilical cord progenitor cells of agouti (Dasyprocta prymnolopha) raised in captivity	2019	
465	Exclude	wrong outcome	Su141decreased Cerebroplacental Ratio Is Associated with Biological Age Deceleration: An Epigenetic Approach Based on a Clinical Population Exposed to Obstetric Complications	2019	
466	Exclude	wrong outcome	The association of patient preferences and attitudes with trial of labor after cesarean	2019	
467	Exclude	wrong outcome	The effects of ultrasound-targeted microbubble destruction (UTMD) carrying IL-8 monoclonal antibody on the inflammatory responses and stability of atherosclerotic plaques	2019	
468	Exclude	wrong population	The Study of Predictive Value of Uterine Artery Doppler in Incidence of Preeclampsia and Intrauterine Growth Restrictions in Pregnant	2019	
469	Exclude	wrong outcome	The transition from latent to active labor and adverse obstetrical	2019	
470	Exclude	wrong outcome	Tidal volume via circumferences of the upper body: a pilot study	2019	
471	Exclude	registration	To study the occurance of low blood pressure (hypotension) after giving spinal anaesthesia in pregnant patients and its relation with values of perfusion index (which is measured by an instrument based on the blood flow in the finger)	2019	
472	Exclude	wrong outcome	Transcranial Doppler role in prediction of post-dural puncture headache in parturients undergoing elective cesarean section: prospective observational study	2019	
473	Exclude	wrong outcome	Transient neurological symptoms (TNS) following spinal anaesthesia with lidocaine versus other local anaesthetics in adult surgical patients: a network meta-analysis	2019	
474	Exclude	wrong outcome	Ultrasonic Pocket Doppler, novel technology for fetal and neonatal heart rate assessment	2019	
475	Exclude	wrong population	Ultrasound, CT, MRI, or PET-CT for staging and re-staging of adults with cutaneous melanoma	2019	
476	Exclude	wrong outcome	Unusual presentation of systemic lupus erythematosus during	2019	
477	Exclude	wrong outcome	Uterine and umbilical artery doppler in women with pre-eclampsia and their pregnancy outcomes	2019	
478	Exclude	wrong outcome	Uterine artery Doppler ultrasound in second pregnancy with previous elective cesarean section()	2019	
479	Exclude	wrong outcome	Women receiving massive transfusion due to postpartum hemorrhage: A comparison over time between two nationwide cohort	2019	
480	Exclude	wrong outcome	Women's Preferences Regarding the Processes and Outcomes of Trial of Labor After Cesarean and Elective Repeat Cesarean Delivery	2019	

481	Exclude	wrong study design	Young female patients after intrauterine fetal demise myocardial	2019	
482	Exclude	wrong outcome	Adherence to a healthy eating index for pregnant women is associated with lower neonatal adiposity in a multiethnic Asian cohort: the Growing Up in Singapore Towards healthy Outcomes	2018	
483	Exclude	wrong outcome	An analysis of the effects of intrapartum factors, neonatal characteristics, and skin-to-skin contact on early breastfeeding	2018	
484	Exclude	wrong population	An observational study: The utility of perfusion index as a discharge criterion for pain assessment in the postanesthesia care unit	2018	
485	Exclude	wrong outcome	Are intermittently elevated UA Doppler Pulsatility Indices associated with increased risk of neonatal morbidity in pregnancies with intrauterine growth restriction?	2018	
486	Exclude	wrong outcome	Association of maternal ectopic lipid stores and organ-specific insulin sensitivity with neonatal anthropometric measures	2018	
487	Exclude	wrong outcome	Body Fatness and Cardiovascular Health in Newborn Infants	2018	
488	Exclude	wrong outcome	Can cerebroplacental ratios predict perinatal and delivery outcomes for viable fetuses?	2018	
489	Exclude	wrong outcome	Care prior to and during subsequent pregnancies following stillbirth for improving outcomes	2018	
490	Exclude	wrong outcome	Casting doubt on the value of assessing the cardiac index in	2018	
491	Exclude	wrong outcome	Conference Abstract	2018	
492	Exclude	wrong population	Cerebral and Renal Oxymetry and Anesthetic Techniques in	2018	
493	Exclude	wrong outcome	Clinical characteristics and pregnancy outcomes in parturients with pulmonary hypertension: experience with 39 consecutive cases from	2018	
494	Exclude	wrong population	Clinical characteristics of acute pancreatitis in pregnancy: experience based on 121 cases	2018	
495	Exclude	wrong outcome	Colour Doppler Study of Foetomaternal Circulation in Pih, Oligohydramnios and Its Perinatal Outcome	2018	
496	Exclude	wrong outcome	Combination of serum histidine-rich glycoprotein and uterine artery Doppler to predict preeclampsia	2018	
497	Exclude	wrong outcome	Comparison of fetal middle cerebral artery versus umbilical artery color Doppler ultrasound for predicting neonatal outcome in complicated pregnancies with fetal growth restriction	2018	
498	Exclude	wrong outcome	Comparison of fetal thigh fractional limb volume with neonatal ponderal index in fetal growth restriction	2018	
499	Exclude	wrong outcome	Comparison of phenylephrine versus norepinephrine for maintenance of hemodynamic during cesarean section under spinal anesthesia	2018	
500	Exclude	wrong outcome	Comparison of TAP, Anterior QL, or ESP Block for Elective Cesarean Section	2018	

501	Exclude	wrong outcome	Continuous intravenous perioperative lidocaine infusion for postoperative pain and recovery in adults	2018	
502	Exclude	wrong outcome	Correlation of impression depth of umbilical cord around neck with blood flow indexes of umbilical artery and fetus middle cerebral artery	2018	
503	Exclude	wrong population	Diabetes and Osteopathic Manipulative Medicine (OMM)	2018	
504	Exclude	registration	Different Preloads for Prevention of Hypotension in Patients Undergoing Elective CS Under Intrathecal Anesthesia	2018	
505	Exclude	wrong outcome	Discontinuation of intravenous oxytocin in the active phase of induced labour	2018	
506	Exclude	wrong outcome	Discrepant Doppler values in paired umbilical arteries in the growth restricted fetus and the relationship to fetal biometry	2018	
507	Exclude	wrong population	Disparities of Clinical Features and Associated Maternal Factors among Symmetrical and a Symmetrical Intra-Uterine Growth Restriction (IUGR) in NICU at Al-Yarmouk Teaching Hospital in	2018	
508	Exclude	wrong outcome	Doppler measurements of both umbilical arteries do not improve predictive value for adverse perinatal outcomes in small-for-	2018	
509	Exclude	wrong outcome	Doppler Study of the Fetal Renal Artery in Oligohydramnios with Post-term Pregnancy	2018	
510	Exclude	wrong outcome	Effect on Health Care Costs for Adolescents Receiving Adjunctive Internet-Delivered Cognitive-Behavioral Therapy: Results of a Randomized Controlled Trial	2018	
511	Exclude	wrong outcome	Effects of gestational hypertension in the pulsatility index of the middle cerebral and umbilical artery, cerebro-placental ratio, and associated adverse perinatal outcomes	2018	
512	Exclude	wrong outcome	Electronic Confirmed Versus Conventional Consenting Process: a Randomized Controlled Trial	2018	
513	Exclude	wrong outcome	Epidural analgesia versus patient-controlled intravenous analgesia for pain following intra-abdominal surgery in adults	2018	
514	Exclude	wrong outcome	Evaluation of psychosocial and biological parameters in women seeking for a caesarean section and women who are aiming for vaginal delivery: a cross-sectional study	2018	
515	Exclude	registration	Evaluation of volume and hypotension after spinal anesthesia with ultrasonic measurement of different veins during cesarean section	2018	
516	Exclude	wrong population	Exercise and Weight Control	2018	
517	Exclude	wrong population	Feasibility of Oxygen Saturation Monitoring During Neonatal Resuscitation in Indian Scenario	2018	
518	Exclude	wrong outcome	Fetal Biometry and Doppler Study for the Assessment of Perinatal Outcome in Stage I Late-Onset Fetal Growth Restriction	2018	

519	Exclude	wrong outcome	Fetal Doppler to predict cesarean delivery for non-reassuring fetal status in the severe small-for-gestational-age fetuses of late preterm	2018	
520	Exclude	wrong outcome	Feto-maternal outcome in women with corrected tetralogy of fallot: A-5-tear experience	2018	
521	Exclude	wrong outcome	Group Intervention for Tobacco Smoking Cessation (GRITS) Phase II: a Pragmatic Randomized Control Trial Comparing Group and Individual Therapy (Standard Care) to Assist Patients to Quit	2018	
522	Exclude	wrong population	Immunity Modification of Full Term Infants According to the Type of Feeding and Mode of Delivery	2018	
523	Exclude	wrong outcome	Impact of Intended Mode of Delivery on Outcomes in Preterm Growth-Restricted Fetuses	2018	
524	Exclude	wrong index test	Impedance cardiography as tool for continuous hemodynamic monitoring during cesarean section: randomized, prospective double	2018	
525	Exclude	wrong outcome	Improving maternal-infant bonding after prenatal diagnosis of CHD	2018	
526	Exclude	wrong outcome	Infection-induced thrombin production: a potential novel mechanism for preterm premature rupture of membranes (PPROM)	2018	
527	Exclude	wrong population	Injectable local anaesthetic agents for dental anaesthesia	2018	
528	Exclude	wrong outcome	Interventions for investigating and identifying the causes of stillbirth	2018	
529	Exclude	wrong population	Interventions for preventing intensive care unit delirium in adults	2018	
530	Exclude	review article	Intraoperative interventions for preventing surgical site infection: an overview of Cochrane Reviews	2018	
531	Exclude	wrong population	Intraoperative use of low volume ventilation to decrease postoperative mortality, mechanical ventilation, lengths of stay and lung injury in adults without acute lung injury	2018	
532	Exclude	wrong outcome	Leadless cardiac dual-chamber pacing	2018	
533	Exclude	wrong outcome	Lifestyle Interventions Limit Gestational Weight Gain in Women with Overweight or Obesity: LIFE-Moms Prospective Meta-Analysis	2018	
534	Exclude	wrong outcome	Local anaesthetics and regional anaesthesia versus conventional analgesia for preventing persistent postoperative pain in adults and	2018	
535	Exclude	wrong outcome	Maternal cardiovascular hemodynamics in normotensive versus preeclamptic pregnancies: a prospective longitudinal study using a noninvasive cardiac system (NICaS)	2018	
536	Exclude	wrong outcome	Maternal nutrition, obesity, and diabetes: Effects on infant growth and body composition	2018	
537	Exclude	wrong outcome	Maternal position in the second stage of labour for women with epidural anaesthesia	2018	
538	Exclude	wrong population	Mechanical versus manual chest compressions for cardiac arrest	2018	
539	Exclude	wrong outcome	Metformin treatment of type 2 diabetes mellitus in pregnancy: update on safety and efficacy	2018	

540	Exclude	wrong population	Methylphenidate for attention deficit hyperactivity disorder (ADHD) in children and adolescents - assessment of adverse events in non-randomised studies	2018	
541	Exclude	wrong outcome	Microbiome and Allergic Diseases	2018	
542	Exclude	wrong outcome	My patient has received fluid. How to assess its efficacy and side	2018	
543	Exclude	wrong population	New Insights on Early Patterns of Respiratory Disease among Extremely Low Gestational Age Newborns	2018	
544	Exclude	wrong population	Non-contact measurement of tidal breathing using structured light plethysmography (SLP) to support diagnosis in undiagnosed COPD	2018	
545	Exclude	wrong study design	Nutritional intra-amniotic therapy increases survival in a rabbit model of fetal growth restriction	2018	
546	Exclude	wrong population	Observations of Pediatric Disease Prevalence from Pacific Partnership 2015	2018	
547	Exclude	wrong outcome	Optimal glycaemic control during caesarean section provided by sensor-augmented pump therapy with predictive low-glucose	2018	
548	Exclude	wrong outcome	Optimal skin antiseptic agents for prevention of surgical site infection in cesarean section: a meta-analysis with trial sequential analysis	2018	
549	Exclude	wrong study design	Oral/Free Communication Session Abstracts	2018	
550	Exclude	wrong population	Perfusion index and pleth variability index in the first hour of life according to mode of delivery	2018	
551	Exclude	wrong outcome	Perinatal outcomes in uncomplicated late preterm pregnancies with borderline oligohydramnios	2018	
552	Exclude	wrong outcome	Perioperative mortality rates in low-income and middle-income countries: a systematic review and meta-analysis	2018	
553	Exclude	wrong outcome	Placental edge to internal os distance and mode of delivery in low-lying placentas	2018	
554	Include		Pleth variability index can predict spinal anaesthesia-induced hypotension in patients undergoing caesarean delivery	2018	https://doi.org/10.1111/aas.13012
555	Exclude	wrong outcome	Postpartum emergency department usage among women with psychiatric illness	2018	
556	Exclude	wrong outcome	Predictive modeling of emergency cesarean delivery	2018	
557	Exclude	wrong outcome	Progression of events in multi-vessel doppler studies in a fetus with growth restriction	2018	
558	Exclude	wrong population	Provision of respiratory support compared to no respiratory support before cord clamping for preterm infants	2018	
559	Exclude	wrong population	Psychological therapies for anxiety and depression in children and adolescents with long-term physical conditions	2018	
560	Exclude	wrong population	Psychological therapies for the management of chronic and recurrent pain in children and adolescents	2018	

561	Exclude	wrong outcome	Randomized Controlled Trial to Prevent Infant Overweight in a High-Risk Population	2018	
562	Exclude	wrong outcome	Reference centile charts of first-trimester aneuploidy screening & Doppler parameters for Indian population	2018	
563	Exclude	wrong population	Respiratory inductive plethysmography (RIP) andoutcomes in children with cerebral palsy	2018	
564	Exclude	wrong outcome	Screening for adverse perinatal outcomes: uterine artery Doppler, cerebroplacental ratio and estimated fetal weight in low-risk women	2018	
565	Exclude	wrong study design	SIRENOMELIA AND ITSCONGENITAL MALFORMATIONS INPREGNANCY WITHOUT PRENATALDIAGNOSIS: A CASE	2018	
566	Exclude	wrong outcome	The Assessment of Association between Uterine Artery Pulsatility Index at 30-34 Week's Gestation and Adverse Perinatal Outcome	2018	
567	Exclude	wrong outcome	The value of 3-dimensional color Doppler in predicting intraoperative hemorrhage for cesarean scar pregnancy	2018	
568	Exclude	wrong outcome	The Zika Virus Infection in Pregnancy: Review and Implications for Research and Care of Women and Infants in Affected Areas	2018	
569	Exclude	wrong outcome	Third Trimester Modified Biophysical Profile Scan for Predicting Fetal Outcome	2018	
570	Exclude	wrong population	To describe the body composition of neonates born to mothers with gestational diabetes melitus	2018	
571	Exclude	wrong outcome	Trends in Hispanic and non-Hispanic white cesarean delivery rates on the US-Mexico border, 2000-2015	2018	
572	Exclude	wrong outcome	Use of placental growth factor and uterine artery doppler pulsatility index in pregnancies involving intrauterine fetal growth restriction or preeclampsia to predict perinatal outcomes	2018	
573	Exclude	wrong population	Use of pre-emptive ephedrine guided byperfusion index in the management of maternal hypotension secondary to spinal anaesthesia: a randomised controlled trial	2018	
574	Exclude	wrong outcome	Using appropriate pre-pregnancy body mass index cut points for obesity in the Chinese population: a retrospective cohort study	2018	
575	Exclude	wrong outcome	Validation of the Delphi Procedure Consensus criteria for defining Fetal Growth Restriction (FGR)	2018	
576	Exclude	registration	Vasopressor of choice for spinal hypotension in elective Cesarean	2018	
577	Exclude	wrong outcome	Ritonavir Pre-Clinical Animal StudiesOutcome of Cryptorchidism Not Supported byReal-World Evidence in Humans	2017	
578	Exclude	wrong outcome	[Assessment of relationship between Doppler flows in pregnancies after 41 week and the incidence of cesarean sections with induced	2017	

579	Exclude	wrong outcome	[Examination of placental three-dimensional power Doppler indices and perinatal outcome in pregnancies complicated by intrauterine growth restriction]	2017	
580	Exclude	wrong publication type	37th Annual Meeting of the Society for Maternal-Fetal Medicine: The Pregnancy Meeting	2017	
581	Exclude	wrong outcome	A randomized controlled trial of chlorhexidine-alcohol versus povidone-iodine for cesarean antisepsis	2017	
582	Exclude	duplicate	A randomized controlled trial of chlorhexidine-alcohol versus povidone-iodine for cesarean antisepsis	2017	
583	Exclude	wrong outcome	A survey of surgical site infection prevention strategies among U. S. Hospitals	2017	
584	Exclude	wrong outcome	Abnormal MCA Dopplers in Diabetic Patients and the Association with Stillbirth.	2017	
585	Exclude	wrong outcome	Blood flow changes in pelvic vessels associated with the application of an abdominal compression belt in healthy postpartum women	2017	
586	Exclude	wrong population	Body positioning for spontaneously breathing preterm infants with	2017	
587	Exclude	wrong population	Cerebroplacental Doppler ratio use in predicting and preventing adverse perinatal outcomes in intrauterine growth restriction	2017	
588	Exclude	wrong outcome	Cesarean sections in Piaui State: trend and associated factors in the period 2000-2011, Brazil	2017	
589	Exclude	wrong outcome	Clinical and ultrasound parameters in prediction of excessive hemorrhage during management of cesarean scar pregnancy	2017	
590	Exclude	wrong outcome	Combined diet and exercise interventions for preventing gestational diabetes mellitus	2017	
591	Exclude	wrong outcome	Comparison of Outcome of Normal and High-Risk Pregnancies Based Upon Cerebroplacental Ratio Assessed by Doppler Studies	2017	
592	Exclude	wrong outcome	Comparison of Two Screening Strategies for Gestational Diabetes (GDM(2)) Trial: Design and rationale	2017	
593	Exclude	wrong outcome	Day-to-day experience in resolution of pain after surgery	2017	
594	Exclude	wrong population	Design of a Closed-loop Controller Based on the Bispectral Index (BIS) Effectiveness of the Smith Predictor	2017	
595	Exclude	wrong population	Determinants of cerebral oxygenation in preterm infants <30 weeks	2017	
596	Exclude	wrong population	Diet, physical activity and behavioural interventions for the treatment of overweight or obese children from the age of 6 to 11 years	2017	
597	Exclude	wrong outcome	Differential hypertensive protease expression in the thoracic versus abdominal aorta	2017	
598	Include		Differential Roles of the Right and Left Toe Perfusion Index in Predicting the Incidence of Postspinal Hypotension During Cesarean	2017	https://doi.org/10.1213/ANE.0000000000002393

599	Exclude	wrong outcome	Does ligation of internal iliac artery for postpartum hemorrhage affect clitoral artery blood flow and postpartum sexual functions?	2017	
600	Exclude	wrong outcome	Effect of Transdermal Nitroglycerine on Doppler Velocity Waveforms of the Uterine, Umbilical and Fetal Middle Cerebral Arteries in Patients with Chronic Placental Insufficiency: A Prospective RCT	2017	
601	Exclude	wrong study design	Effects of systemic pyruvate on cerebral and systemic hemodynamics in newborn lambs following asphyxial cardiac arrest	2017	
602	Exclude	wrong outcome	Efficacy and safety of misoprostol, dinoprostone and Cook's balloon for labour induction in women with foetal growth restriction at term	2017	
603	Exclude	wrong outcome	Elective Caesarean section on maternal request in Germany: factors affecting decision making concerning mode of delivery	2017	
604	Exclude	wrong outcome	Estimation of Toxicities Following Stereotactic Ablative Radiation Therapy Treatment of Locally Recurrent and Previously Irradiated Head and Neck Squamous Cell Carcinoma Based on a Normal Tissue Complication Probability Model	2017	
605	Exclude	wrong outcome	Evaluation of Institute of Medicine Guidelines for Gestational Weight Gain in Women with Chronic Hypertension	2017	
606	Exclude	wrong outcome	Exploring sexual dimorphism in placental circulation at 22-24 weeks of gestation: A cross-sectional observational study	2017	
607	Exclude	wrong outcome	Fetal and umbilical Doppler ultrasound in high-risk pregnancies	2017	
608	Exclude	registration	Fluid therapy in pregnancy in cesarian section	2017	
609	Exclude	wrong population	General Endoscopy	2017	
610	Exclude	wrong study design	Glycogen storage disease type IIIa in pregnancy: A case presentation	2017	
611	Exclude	wrong population	Haemodynamic Transition after Birth: A New Tool for Non-Invasive Cardiac Output Monitoring	2017	
612	Exclude	wrong outcome	HPV testing in first-void urine provides sensitivity for CIN2+ detection comparable with a smear taken by a clinician or a brush-based self-sample: cross-sectional data from a triage population	2017	
613	Exclude	wrong outcome	Identification of proteomic changes associated with placenta accreta	2017	
614	Exclude	wrong outcome	Impact of Interval between Screening and Diagnosis of Gestational Diabetes on Pregnancy Outcomes	2017	
615	Exclude	wrong population	Impact of mode of delivery on skin microcirculation in term healthy newborns within the first day of life	2017	
616	Exclude	wrong outcome	Improving the wellbeing of people with opioid treated chronic pain – the i-wotch study protocol	2017	
617	Exclude	duplicate	Improving the wellbeing of people with opioid treated chronic pain – the i-wotch study protocol	2017	
618	Exclude	wrong index test	Intraoperative Hypotonia: Significance and Monitoring in the clinical Practice	2017	

619	Exclude	wrong outcome	Isolated Umbilical Artery Doppler Velocimetry in Intrauterine Growth Restricted Foetuses with Correlation of Foetal Outcome	2017	
620	Exclude	wrong population	Low values of central venous oxygen saturation (ScvO2) during surgery and anastomotic leak of abdominal trauma patients	2017	
621	Exclude	wrong population	Methylphenidate for children and adolescents with autism spectrum disorder	2017	
622	Exclude	wrong outcome	Mother to child transmission (MTCT) of HIV - Almost a thing of the past? A cohort study of HIV positive women starting antiretroviral drugs in pregnancy	2017	
623	Exclude	wrong outcome	Necrotizing gastritis and perforation in an extremely low birthweight	2017	
624	Exclude	wrong outcome	New approach in treatment of selective IUGR in dichorionic twins with pravastatin	2017	
625	Exclude	wrong population	Non-invasive ventilation for cystic fibrosis	2017	
626	Exclude	wrong outcome	Obstetric and perinatal outcome in anti-Ro/SSA-positive pregnant women: a prospective cohort study	2017	
627	Exclude	wrong outcome	Open-label, multicenter, dose escalation phase 1b study to assess the subcutaneous delivery of daratumumab in patients (pts) with relapsed or refractory multiple myeloma (PAVO)	2017	
628	Exclude	wrong outcome	Pain, Anxiety, and Fatigue During Labor: A Prospective, Repeated Measures Study	2017	
629	Include		Perfusion index as a predictor of hypotension following spinal anaesthesia in lower segment caesarean section	2017	https://doi.org/10.4103/ija.IJA_429_16. e30431
630	Exclude	wrong outcome	Periodontitis and Adverse Pregnancy Outcomes in Metabolic Syndrome Patients- Interventional Study	2017	
631	Exclude	wrong study design	Perioperative use of transthoracic echocardiography in a patient with congenitally corrected transposition of great arteries, atrial septal defect and severe pulmonary stenosis for lower segment cesarean	2017	
632	Exclude	wrong population	Physiological effect of high-flow nasal cannula on respiratory pattern and work of breathing in severe COPD patients	2017	
633	Exclude	wrong outcome	Placental pulsatility index: a new, more sensitive parameter for predicting adverse outcome in pregnancies suspected of fetal growth	2017	
634	Exclude	wrong outcome	Placental telomere length in preterm fetal growth restriction due to placental insufficiency	2017	
635	Exclude	wrong outcome	PlGF in a clinical setting of pregnancies at risk of preeclampsia and/or intrauterine growth restriction	2017	
636	Exclude	wrong outcome	Prediction of delivery of small-for-gestational-age neonates and adverse perinatal outcome by fetoplacental Doppler at 37 weeks'	2017	

637	Exclude	retracted	Preoperative chlorhexidine versus povidone-iodine antisepsis for preventing surgical site infection: A meta-analysis and trial sequential analysis of randomized controlled trials	2017	
638	Exclude	wrong population	Prevalence and pattern of birth defects in a tertiary health facility in the Niger Delta area of Nigeria	2017	
639	Exclude	wrong outcome	Preventing shivering with adjuvant low dose intrathecal meperidine: A meta-analysis of randomized controlled trials with trial sequential	2017	
640	Exclude	wrong outcome	Primary Cesarean Delivery Patterns among Women with Physical, Sensory, or Intellectual Disabilities	2017	
641	Exclude	wrong study design	Pseudoamniotic Band Syndrome after In Utero Intervention for Twin-to-Twin Transfusion Syndrome: Case Reports and Literature Review	2017	
642	Exclude	wrong outcome	Pulse Rate and Transit Time Analysis to Predict Hypotension Events After Spinal Anesthesia During Programmed Cesarean Labor	2017	
643	Exclude	wrong outcome	Relationship between total bile acid concentration and fetal pulmonary surfactant in intrahepatic cholestasis of pregnancy	2017	
644	Exclude	wrong outcome	Renal Measures in Healthy Italian Trotter Foals and Correlation Between Renal and Biometric Measures: Preliminary Study	2017	
645	Exclude	wrong outcome	Serial ultrasound measurements of fetal head circumference and abdominal circumference to predict fetal growth restriction in a Sri Lankan study population	2017	
646	Exclude	wrong population	Singing for adults with chronic obstructive pulmonary disease	2017	
647	Exclude	wrong outcome	Single dose oral ketoprofen or dexketoprofen for acute postoperative pain in adults	2017	
648	Exclude	wrong study design	Sixth cranial nerve palsy as the manifestation of preeclampsia	2017	
649	Exclude	wrong population	Target value of oxygen saturation during the first 10 min after birth	2017	
650	Exclude	wrong publication type	Technology and computing for the sake of the physician : The abstracts of the 26th congress of ESCTAIC, Timisoara, Romania, September 22-24 2016	2017	
651	Exclude	wrong population	TEE as a Guide for Fluid Optimization in Major Abdominal	2017	
652	Exclude	wrong outcome	The association between first trimester uterine artery Doppler velocimetry indices and adverse perinatal outcomes in IVF cycles	2017	
653	Exclude	wrong outcome	The fetal cerebro-placental ratio in diabetic pregnancies is influenced more by the umbilical artery rather than middle cerebral artery	2017	
654	Exclude	wrong outcome	The impact of life-long maternal HIV infection on pregnancy outcomes in women delivering in south carolina	2017	
655	Exclude	wrong outcome	The Labor Analgesia Requirements in Nulliparous Women Randomized to Epidural Catheter Placement in a High or Low	2017	
656	Exclude	wrong study design	The potential of human amniotic membrane proteins in cardiac regeneration; the Role of extraction methods	2017	

657	Exclude	wrong outcome	Time trends in births and cesarean deliveries among women with disabilities	2017	
658	Exclude	wrong population	Total intravenous anaesthesia versus inhalational anaesthesia for adults undergoing transabdominal robotic assisted laparoscopic	2017	
659	Exclude	wrong outcome	Transitioning to normal breathing in preterm and term pups during the first hours of birth	2017	
660	Exclude	wrong study design	Transparent anemone shrimp (Ancylomenes pedersoni) become opaque after exercise and physiological stress in correlation with increased hemolymph perfusion	2017	
661	Exclude	wrong outcome	Treating periodontal disease for preventing adverse birth outcomes in pregnant women	2017	
662	Exclude	wrong study design	TRIPLETS– TRIPLE COMPLICATION (CASE REPORT)	2017	
663	Exclude	wrong outcome	Tubocutaneous Fistula due to Endometriosis - A Differential Diagnosis in Cutaneous Fistulas with Cyclic Secretion	2017	
664	Exclude	wrong outcome	Ultrasound criteria in early versus late IUGR Romanian experience	2017	
665	Exclude	wrong outcome	Ultrasound in Twin Pregnancies	2017	
666	Exclude	wrong outcome	Ultrasound markers predictiong complex gastroschisis and adverse outcome: A longitudinal prospective nationwide cohort study	2017	
667	Exclude	wrong outcome	Umbilical artery doppler assessment at term in healthy nulliparous patients- is it ever necessary? Result from the multicenter	2017	
668	Exclude	duplicate	Umbilical artery doppler assessment at term in healthy nulliparous patients- is it ever necessary? Result from the multicenter	2017	
669	Exclude	wrong outcome	Uterine artery doppler ultrasound predictor of adverse pregnancy outcomes	2017	
670	Exclude	wrong outcome	Vaginal misoprostol and cervical ripening balloon for induction of labor in late-term pregnancies	2017	
671	Exclude	wrong population	Warm and Humidified vs Cold and Dry Carbon Dioxide (CO2) Pneumoperitoneum	2017	
672	Exclude	wrong outcome	What can we do to reduce the associated costs in induction of labour of intrauterine growth restriction foetuses at term? A cost-analysis	2017	
673	Exclude	wrong study design	When twin anemia sequence (TAPS) and twin-to twin transfusion syndrome (TTTS) coexist	2017	
674	Exclude	wrong outcome	Ductus venosus flow velocity waveforms indices and arterial cord pH in IUGR fetuses delivered at 32 weeks	2016	
675	Exclude	wrong outcome	[Prenatal care and risk factors associated with premature birth and low birth weight in the a capital in the Brazilian Northeast]	2016	
676	Exclude	wrong outcome	Conference Abstract (10th World congress of peronatal medicine)	2016	
677	Exclude	wrong study design	Conference Abstract (10th World congress of peronatal medicine)	2016	

678	Exclude	wrong publication type	Conference Abstract (33rd Congress of the Scandinavian Society of Anaesthesiology and Intensive Care Medicine, Reykjavik, Iceland, 10 June 2015)	2016	
679	Exclude	wrong publication type	Conference Abstract (34th Annual Meeting of the Society for Maternal-Fetal Medicine: The Pregnancy Meeting)	2016	
680	Exclude	wrong index test	3d Power Doppler in Obstetrics	2016	
681	Exclude	wrong outcome	3TC+ PI dual therapy during pregnancy for PMTCT of HIV-1-naive or pretreated women	2016	
682	Exclude	wrong outcome	Conference Abstract (11th World congress of peronatal medicine)	2016	
683	Exclude	wrong outcome	Conference Abstract (2013 World congress of peronatal medicine)	2016	
684	Exclude	wrong study design	A case of conjoined twins (thoracopagus)	2016	
685	Exclude	wrong outcome	A comparative study of three indices of umbilical blood flow in relation to prediction of growth retardation	2016	
686	Exclude	wrong outcome	A comparison of baseline characteristics between diagnosed gestational diabetes mellitus (GDM) patients adopting the one versus two step approach to diagnosis	2016	
687	Exclude	wrong outcome	A quadruplet pregnancy complicated by gestational diabetes mellitus: Obstetrical management and neonatal outcomes	2016	
688	Exclude	wrong outcome	A randomised controlled trial of the probiotic Bifidobacterium breve BBG-001 in preterm babies to prevent sepsis, necrotising enterocolitis and death: the Probiotics in Preterm infantS (PiPS) trial	2016	
689	Exclude	wrong outcome	A review if clinical outcomes in an UK HIV antenatal clinic	2016	
690	Exclude	wrong population	Abdominal compartment syndrome relieved by percutaneous drainage in an infant with critical pertussis supported on venovenous extracorporeal membrane oxygenation	2016	
691	Exclude	wrong outcome	Abstracts and Highlight Papers of the 34th Annual European Society of Regional Anaesthesia & Pain Therapy (ESRA) Congress 2015	2016	
692	Exclude	wrong population	ACCURACY OF SYNCHRONISATION DURING SiPAP GENERATED NASAL INTERMITTENT POSITIVE PRESSURE VENTILATION (NIPPV) IN PRETERM INFANTS	2016	
693	Exclude	wrong population	Acquired haemophilia: Why the delay in diagnosis?	2016	
694	Exclude	wrong study design	Acral necrosis and upper brachial plexus palsy after prenatal fetal thrombosis	2016	
695	Exclude	wrong population	Acupuncture and related interventions for symptoms of chronic kidney disease	2016	
696	Exclude	wrong study design	Acute aortic syndrome in pregnancy: Endovascular Management	2016	
697	Exclude	wrong outcome	Acute atherosis in vacuum suction biopsies of decidua basalis: An evidence based research definition	2016	

698	Exclude	wrong outcome	An adult looking at the management of HIV-positive pregnant women at two large UK centres	2016	
699	Exclude	review article	ANAESTHESIA for CAESAREAN SECTION - Anestesianel taglio cesareo. II	2016	
700	Exclude	wrong outcome	Anaesthetic interventions for prevention of awareness during surgery	2016	
701	Exclude	wrong outcome	Antiretroviral therapy and pregnancy: effect on cortical bone status of HIV-infected women	2016	
702	Exclude	wrong study design	Antiretrovirals and pregnancy: A case series	2016	
703	Exclude	wrong outcome	Aortic, pulmonary, and ductal peak velocities in symmetrical and asymmetrical fetal growth retardation: A different adaptation to placental insufficiency	2016	
704	Exclude	wrong outcome	Application of the perfusion index in obstetric bleeding	2016	
705	Exclude	wrong outcome	Assessment in the perinatal period of the newborns from ART conceived pregnancies	2016	
706	Exclude	wrong outcome	Assessment of uterine arterial blood flow using color and pulsed Doppler ultrasound in pre-eclampsia	2016	
707	Exclude	wrong outcome	Association of suboptimal Dopplers with maternal obesity-an	2016	
708	Exclude	wrong outcome	Associations between perinatal hemorrhage and acute renal failure: Race/ ethnicity disparity	2016	
709	Exclude	wrong index test	Bedside analysis of heart rate variability by Analgesia Nociception Index (ANI) predicts hypotension after spinal anesthesia for elective Caesarean delivery	2016	
710	Exclude	wrong outcome	Biophysical and biochemical markers at 30-34 weeks' gestation in the prediction of adverse perinatal outcome	2016	
711	Exclude	wrong outcome	Biophysical and biochemical markers at 35-37 weeks' gestation in the prediction of adverse perinatal outcome	2016	
712	Exclude	wrong outcome	Brain sparing effect in growth-restricted fetuses is associated with decreased cardiac acceleration and deceleration capacities: a case-control study	2016	
713	Exclude	wrong outcome	Can a zero perinatal transmission rate of HIV be achieved in the US?	2016	
714	Exclude	wrong outcome	Can Ultrasound Predict Increased Neonatal Body Fat In Gestational Diabetes?	2016	
715	Exclude	wrong outcome	Case study: presentation, evolution and outcome of fetal haemorrhagic stroke	2016	
716	Exclude	wrong outcome	Cerebral doppler evaluation in prolonged pregnancy and perinatal	2016	
717	Exclude	wrong outcome	Cerebral Hemodynamic Effects of Intravenous Oxytocin Bolus at the Time of Cesarean Delivery	2016	

718	Exclude	wrong study design	Characteristics of porcine circovirus-2 replication in lymphoid organs of pigs inoculated in late gestation or postnatally and possible relation to clinical and pathological outcome of infection	2016	
719	Exclude	wrong outcome	Chronic Thromboembolic Pulmonary Hypertension (CTEPH) Diagnosed During Pregnancy	2016	
720	Exclude	wrong outcome	Clinical Diabetes/Therapeutics	2016	
721	Exclude	duplicate	Comparison of perinatal outcome in growth restricted fetuses retaining normal umbilical artery Doppler flow to those with diminished end-diastolic flow	2016	
722	Exclude	wrong outcome	Continuous subcutaneous insulin infusion versus multiple daily injections of insulin for pregnant women with diabetes	2016	
723	Exclude	wrong outcome	Current treatment strategies,,complications and considerations for the use of HIV antiretroviral therapy during pregnancy	2016	
724	Exclude	wrong outcome	Deep vein thrombosis in pregnancy: Management and results	2016	
725	Exclude	wrong outcome	Detection of late pregnancy large-for-gestational age fetuses (lga) by ultrasound and doppler evaluation at routine third trimester scan in adequate-for-gestational age (aga) pregnancies	2016	
726	Exclude	wrong study design	Dexmedetomidine for Cesarean Section	2016	
727	Exclude	wrong outcome	Diagnosis and follow-up of in patients diagnosed with intrauterine growth restriction in our center	2016	
728	Exclude	wrong outcome	Diagnostic value of pulsed Doppler sonography compared with other parameters for suspected placental insufficiency	2016	
729	Exclude	wrong outcome	Do maternal characteristics influence maternal-fetal medicine physicians' willingness to intervene when managing periviable	2016	
730	Exclude	wrong outcome	Does umbilical artery doppler indices at 35-37 weeks predict intrapartum fetal compromise in term normally grown fetuses?	2016	
731	Exclude	wrong outcome	Doppler flow changes in oxytocin versus prostaglandin E1-induced active labor	2016	
732	Exclude	wrong outcome	Doppler flow studies in middle cerebral and umblical arteries in growth retarded and normal pregnancies	2016	
733	Exclude	wrong outcome	Doppler Prediction of Adverse Perinatal Outcome inIntrauterine Growth Restriction	2016	
734	Exclude	wrong outcome	Doppler velocimetry of the fetal middle cerebral artery	2016	
735	Exclude	wrong population	Drug interventions for the treatment of obesity in children and	2016	
736	Exclude	wrong population	Drug-eluting balloon angioplasty versus uncoated balloon angioplasty for peripheral arterial disease of the lower limbs	2016	
737	Exclude	wrong outcome	EARLY DETERMINANTS OF CHILDHOOD OBESITY: WHAT FACTORS CONTRIBUTE TO ITS DEVELOPMENT SIMULTANEOUSLY IN URUGUAYAN CHILDREN 6-23 MONTHS?	2016	

738	Exclude	wrong outcome	Early skin-to-skin contact for mothers and their healthy newborn	2016	
739	Exclude	wrong study design	Effect of betamethasone, surfactant, and positive end-expiratory pressures on lung aeration at birth in preterm rabbits	2016	
740	Exclude	wrong outcome	Effect of different surgical site antiseptic solutions on cesarean section wound complications	2016	
741	Exclude	wrong study design	EFFECT OF INCREASING AND DECREASING POSITIVE END-EXPIRATORY PRESSURE ON THE DISTRIBUTION OF LUNGAERATION AT BIRTH	2016	
742	Exclude	wrong outcome	Effect of low glycaemic index dietary advice in normal pregnancy: The PREGGIO study	2016	
743	Exclude	wrong population	Effect of synchronised sipap-generated nasal intermittent positive pressure ventilation (NIPPV) on tidal volume, in preterm infants	2016	
744	Exclude	wrong outcome	Effects of docosahexaenoic acidsupplementation on doppler flowparameters and birthweight inpregnancies affected by	2016	
745	Exclude	wrong outcome	Effects of Dual-Energy Technique on Radiation Exposure and Image Quality in Pediatric Body CT	2016	
746	Exclude	wrong outcome	Efficacy and safety of the 13.5 mg (total content) levonorgestrel intrauterine contraceptive system in an Asia-pacific population: Results of a phase III study	2016	
747	Exclude	wrong outcome	Efficacy of chlorhexidine gluconate versus povidone iodine for skin disinfection at cesarean section: a randomized controlled trial	2016	
748	Exclude	wrong study design	Enhanced olacental glucose transport mechamism may contribute to the correction of placental insufficiency by direct olacental adenoviral mediated hIGF-1 transfer in a Murine model	2016	
749	Exclude	wrong outcome	Enhancement of the protective effct of Sj23 DNA vaccine against Schistosoma japonicum infection by a novel vaccine delivery vector-PAMAM-Lys	2016	
750	Exclude	wrong outcome	Estimation of maternal cerebrovascular hemodynamics following routine delivery related blood loss	2016	
751	Exclude	wrong study design	Estrogen-related receptor gamma and cytochrome p450 expression in IUGR placentas	2016	
752	Exclude	wrong population	Evaluation of Volumetric Modulated Arc Therapy for High Risk Pediatric Abdominal Neuroblastoma	2016	
753	Exclude	wrong outcome	Examining the Starting Dose of Glyburide in Gestational Diabetes	2016	
754	Exclude	wrong outcome	Excessive gestational weight gain in women without gestational diabetes mellitus is associated with increased neonatal fat mass and hyperinsulinemia	2016	
755	Exclude	wrong outcome	Factors associated with non-adherence to HAART in HIV-positive pregnant women during pregnancy, peri- and postpartum in Lima,	2016	

756	Exclude	wrong population	Features and outcome of cases with agenesis of the pulmonary valve diagnosed in fetal life	2016	
757	Exclude	wrong outcome	Fetal cardiac injury and ductus venosus doppler velocimetry in severe placental insufficiency before 34th week of gestation	2016	
758	Exclude	wrong population	Fetal cardiovascular remodeling persists at 6 months in infants with intrauterine growth restriction	2016	
759	Exclude	wrong outcome	Fetal inflow hydrostatic pressure measurements in the ex-vivo perfused placenta correlate with umbilical artery Doppler RI and PI	2016	
760	Exclude	wrong outcome	Fetal loss syndrome: Approaches to management of pregnant	2016	
761	Exclude	wrong outcome	Fetal middle cerebral to uterine artery pulsatility index ratios in normal and pre-eclamptic pregnancies	2016	
762	Exclude	wrong study design	Fetal tachycardia: case report	2016	
763	Exclude	wrong outcome	Fetal Thymic Size in Intrauterine Growth Restricted Fetuses	2016	
764	Exclude	wrong outcome	Fibronectin in pregnancy-induced hypertension, preeclampsia and placental insufficiency	2016	
765	Exclude	wrong population	General anaesthesia for operative obstetrics	2016	
766	Exclude	wrong outcome	General anaesthesia for operative obstetrics	2016	
767	Exclude	wrong outcome	Gestational Diabetes, Endothelial Damage and Metabolomic	2016	
768	Exclude	wrong publication type	Guidelines for the use and interpretation of assays for monitoring autophagy (3rd edition)	2016	
769	Exclude	wrong outcome	GUT MICROBIOTA IN EARLY CHILDHOOD IN CYSTICFIBROSIS	2016	
770	Exclude	wrong outcome	Haemorrhagic syndromes in children from mothers with placenta	2016	
771	Exclude	wrong outcome	High preterm delivery rates associated with initiation of HAART during pregnancy	2016	
772	Exclude	wrong outcome	High Prevalence of Hypovitaminosis D in Japanese Pregnant Women with Threatened Premature Delivery.	2016	
773	Exclude	wrong outcome	High risk of liver enzyme elevation in pregnant women receiving protease inhibitors	2016	
774	Exclude	wrong population	Hyperbaric oxygen therapy for people with autism spectrum disorder (ASD)	2016	
775	Exclude	wrong outcome	Impact of Early Screening for Gestational Diabetes on Perinatal Outcomes in High-Risk Women	2016	
776	Exclude	wrong outcome	Impact of Fetal Sex on Cord Blood Metabolic Markers at Birth.	2016	
777	Exclude	wrong outcome	Impaired glucose metabolism in HIV-1-seropositivepregnant women: a prospective analysis	2016	
778	Exclude	wrong outcome	Impaired glucose metabolism in HIV-infected pregnant women: a retrospective analysis	2016	
779	Exclude	wrong population	Implanted (brain) Stimulators to Augment stroke Rehabilitation Therapy (iSTART trial)	2016	

780	Exclude	wrong outcome	Increased Sflt-1/Plgf Ratio Is Associated with Unfavorable Hypertensive Pregnancy Outcome	2016	
781	Exclude	duplicate	Influence of epidural analgesia on the labor induced by premature rupture of membranes	2016	
782	Exclude	wrong population	Inhaled alpha1-proteinase inhibitor therapy in patients with cystic	2016	
783	Exclude	wrong study design	Insulin growth factor 1 gene therapy restores altered umbilical artery (UA) doppler indices in a mouse model of growth restriction (IUGR)	2016	
784	Exclude	wrong study design	Intraplacental injection of adenovirus-mediated human insulin- like growth factor-1 modulates placental HIF2α and IGFBP-1 expression on a mouse model of placental insufficiency	2016	
785	Exclude	wrong outcome	Intrauterine Growth Restriction in Singleton Pregnancy-Surveillance and Delivery: An Overview	2016	
786	Exclude	wrong outcome	Is the association between maternal inflammatory bowel disease and adverse perlnatal outcomes modified by maternal race?	2016	
787	Exclude	wrong population	Is the Perfusion Index Useful in Early Detection of High Spinal Subarachnoid Block during Cesarean Section?	2016	
788	Exclude	duplicate	Is the Perfusion Index Useful in Early Detection of High Spinal Subarachnoid Block during Cesarean Section?	2016	
789	Exclude	wrong outcome	Ischemic Conditioning Protects the Microcirculation, Preserves Organ Function, and Prolongs Survival in Sepsis	2016	
790	Exclude	wrong outcome	JGA Keynote Program. The 3rd International Gastrointestinal Consensus Symposium (IGICS)	2016	
791	Exclude	wrong outcome	Labour and Obstetric Complications (EP8)	2016	
792	Exclude	wrong population	Late-onset group B streptococcus sepsis in twins manifesting as cellulitis-adenitis syndrome	2016	
793	Exclude	wrong population	Malrotation with midgut volvulus presrnting as pediatric sepsis	2016	
794	Exclude	wrong study design	Management of an IUGR with an intraabdominally anastomosis of the umbilical artery and vein	2016	
795	Exclude	wrong outcome	Management of fetal growth restriction at >=36 weeks of gestation - What can be achieved by careful fetal monitoring?	2016	
796	Exclude	wrong study design	Massive pulmonary embolism after the caesarean section	2016	
797	Exclude	wrong outcome	Massive pulmonary embolism treated with thrombolytic drugs during pregnancy	2016	
798	Exclude	wrong outcome	Maternal and fetal Doppler pulsatility indices predict adverse pregnancy outcome in uncomplicated pregnancies with borderline-low amniotic fluid index in the early third trimester	2016	
799	Exclude	wrong study design	Maternal and fetal effects of bay 41-2272, a direct soluble guanylate cyclase activator, in a model of preeclampsia and intra uterine growth restriction	2016	

800	Exclude	wrong outcome	Maternal and Fetal Phenotypes in Hypertensive Disorders of Pregnancy Are Greater Determinants of Perinatal Outcome Than Gestational Age at Onset of Hypertension.	2016	
801	Exclude	wrong outcome	Maternal high fat diet decreases placental blood flow and increases the frequency of stillbirth in a non-human primate model of excess	2016	
802	Exclude	wrong outcome	Maternal-Fetal Medicine physicians' practice patterns for 22-week delivery management	2016	
803	Exclude	wrong outcome	Measurement of fetal vascular resistance using a Duplex scanner - A new fetal stress test	2016	
804	Exclude	wrong population	Microcirculation Within The First Minutes And First 24 Hours Of Life In Healthy Term Newborns	2016	
805	Exclude	wrong population	Micronized progesterone in the treatment of imminent necrosis of myoma during pregnancy. Ultrasound change during treatment	2016	
806	Exclude	wrong outcome	Mother-to-child transmission (MTCT) of HIV – almost athing of the past? A cohort study of HIV-positive womenstarting antiretroviral drugs in pregnancy	2016	
807	Exclude	wrong outcome	MRI findings in placental implantation abnormalities (Placenta Accreta, Increta, Percreta)	2016	
808	Exclude	wrong outcome	MRI reveals increased superior vena caval blood flow in human fetuses with congenital heart disease, abnormal placental pathology and neonatal brain white matter changes	2016	
809	Exclude	wrong population	Neonatal adiposity increases the risk of atopic dermatitis during the first year of life	2016	
810	Exclude	wrong outcome	Neonatal Adiposity Is Predicted by Ultrasound Estimates of the Fetal Thigh Volume at 28 Weeks Gestation.	2016	
811	Exclude	wrong outcome	Nitrates for the prevention of cardiac morbidity and mortality in patients undergoing non-cardiac surgery	2016	
812	Exclude	wrong outcome	Oligohydramnios in preterm preeclampsia is an independent risk factor for perinatal morbidity	2016	
813	Exclude	wrong population	Online adaptive MR guided stereotactic body radiation therapy for the treatment of oligometastatic disease of the abdomen and central thorax: Characterization of potential advantages	2016	
814	Exclude	registration	Open lavel single center randomized parallel group trial concerning circulatory dynamics evaluation using PVI (Pleth Variability Index) between preoperative oral rehydration therapy group and prohibited preoperative eating and drinking group targeted at ASA 1or 2 of scheduled cesarean section for...	2016	

815	Exclude	duplicate	Open-label, multicenter, dose escalation phase 1b study to assess the subcutaneous delivery of daratumumab in patients (pts) with relapsed or refractory multiple myeloma (PAVO)	2016	
816	Exclude	wrong outcome	Optimal Admission Cervical Dilation in Spontaneously Laboring	2016	
817	Exclude	wrong study design	Conference Abstract (2013 World congress of peronatal medicine)	2016	
818	Exclude	wrong outcome	Oral presentations O1–O21	2016	
819	Exclude	wrong outcome	Outcomes across a 10-Year period in placenta accreta- rerated pathologies	2016	
820	Exclude	wrong outcome	Oxyhemoglobin saturation and chest wall stability of premature infants at 32 weeks post-menstrual age supported by high-flow nasal	2016	
821	Exclude	wrong population	Paravertebral block versus thoracic epidural for patients undergoing thoracotomy	2016	
822	Exclude	duplicate	Passive compliance of the total respiratory system in newborns after cesarean section	2016	
823	Exclude	wrong population	PERFUSION INDEX AND PLETH VARIABILITY INDEX IN THE FIRST HOUR OF LIFE ACCORDING TO MODE OF DELIVERY	2016	
824	Exclude	wrong population	Perfusion index assessment during transition period of newborns: an observational study	2016	
825	Exclude	wrong population	Perinatal Outcomes in Obese, Over- and Normal- Weight Pregnant Women with GDM (Gestational Diabetes Mellitus) Treated with Detemir and Aspart	2016	
826	Exclude	wrong population	Perioperative fluid volume optimization following proximal femoral	2016	
827	Exclude	duplicate	Perioperative thrombosis prophylaxis: Results of a randomized, prospective, comparative study with hydroxyethyl starch 6% 0.62 and low-dose heparin	2016	
828	Exclude	wrong outcome	Phosphate-containing poly(ethylene glycol) (PEG) hydrogel nanoparticles for prevention of gut-derived sepsis	2016	
829	Exclude	wrong outcome	Physical activities during pregnancy and type of delivery in nulliparae	2016	
830	Exclude	wrong population	Physical exercise training interventions for children and young adults during and after treatment for childhood cancer	2016	
831	Exclude	wrong outcome	Placental Insufficiency in Fetuses That Slow in Growth but Are Born Appropriate for Gestational Age: A Prospective Longitudinal Study	2016	
832	Exclude	wrong outcome	Placental transfer of maraviroc in the ex vivo human cotyledon perfusionmodel and influence of placental ABC transporters	2016	
833	Exclude	wrong study design	Placental vasculature remodeling by adenovairal-mediated placental gene therapy of insulin growth factor (AD-HIGF-i) in a model of placental insufficiency (PI)	2016	
834	Exclude	wrong population	Plethysmography respiratory-induced variability; a jack in the box?	2016	
835	Exclude	wrong outcome	Polonium-210 poisoning: a first-hand account	2016	

836	Exclude	wrong outcome	Positioning for obestetric anaesthesia and its effects on maternal cardiac output and fetal well-being	2016	
837	Exclude	wrong outcome	Postoperative prevention of thrombosis in cesarean section	2016	
838	Exclude	wrong outcome	Postpartum uterine artery blood flow impedance following cesarean section or vaginal delivery	2016	
839	Exclude	wrong population	Povidone Iodine Pleurodesis for Refractory Congenital Chylothorax: A Review of Literature	2016	
840	Exclude	wrong outcome	Povidone-Iodine wound irrigation prior to skin closure at caesarean section to prevent surgical site infection: A randomised controlled trial	2016	
841	Exclude	wrong outcome	Prediction of adverse outcomes with mid-pregnancy seram placental grouth factor levels and uterine artery Dopplers in high risk	2016	
842	Exclude	wrong outcome	Predictors for perioperative injury at cesarean delivery	2016	
843	Exclude	wrong outcome	Pregnancy Outcomes in Women with 1-Hour Glucose Challenge Test >/= 200 mg/dL	2016	
844	Exclude	wrong outcome	Pregnant women with HIV on ART in Europe: how many achieve the aim of undetectable viral load at term and are able to deliver	2016	
845	Exclude	wrong outcome	Preoperative measurement of maternal abdominal circumference relates the initial sensory block level of spinal anesthesia for cesarean section: An observational study	2016	
846	Exclude	wrong outcome	Prevention of mother-to-child transmission of HIV in Latvia, 2008–	2016	
847	Exclude	wrong outcome	Primary Spoken Language and Neuraxial Labor Analgesia Use Among Hispanic Medicaid Recipients	2016	
848	Exclude	wrong outcome	Prolapse of the umbilical cord: A ten year etudy emphasizing cesarﾂｻ ean section	2016	
849	Exclude	wrong outcome	Provider Communication Regarding Psychosocial Factors Predicts Pain Beliefs in Parent and Child	2016	
850	Exclude	wrong outcome	Psychometric Evaluation of 5- and 4-Item Versions of the LATCH Breastfeeding Assessment Tool during the Initial Postpartum Period among a Multiethnic Population	2016	
851	Exclude	wrong population	Puerperal Sepsis with Furniers Syndrome	2016	
852	Exclude	no details of PI or PVI	Pulse wave analysis by digital photoplethysmography to record maternal hemodynamic effects of spinal anesthesia, delivery of the baby, and intravenous oxytocin during cesarean section	2016	
853	Exclude	wrong outcome	Pulse-Induced Continuous Cardiac Output (Picco) Versus Trans-Esophageal Doppler Monitor (Ted) for Optimization of Fluid Management in Patients Undergoing Major Abdominal Surgery. A	2016	
854	Exclude	wrong outcome	Reducing the risk of fetal distress with sildenafil study (RIDSTRESS): a double-blind randomised control trial	2016	

855	Exclude	wrong outcome	Relationship between general movements in neonates who were growth restricted in utero and prenatal Doppler flow patterns	2016	
856	Exclude	wrong outcome	RENAL AND MIDDLE ADRENAL ARTERY DOPPLER VELOCIMETRY - PREDICTIVE MARKERS TO INTRAUTERINE GROWTH RESTRICTION IN HIGH RISK PREGNANCIES	2016	
857	Exclude	wrong outcome	Renal Parenchyma Perfusion During Laparoscopic Donor	2016	
858	Exclude	wrong outcome	Respiratory phenotype in rapid-onset obesity with hypothalamic dysfunction, hypoventilation, and autonomic dysregulation (ROHHAD): Timing is everything	2016	
859	Exclude	wrong outcome	Respiratory response to exogenous ventilatory challenges in Congenital Central Hypoventilation Syndrome (CCHS): PHOX2B genotype/CCHS phenotype association	2016	
860	Exclude	wrong study design	ROLES OF TRANSPULMONARY HYDROSTATIC PRESSURESAND NA+ REABSORPTION IN AIRWAY LIQUID	2016	
861	Exclude	wrong outcome	Saquinavir-based regimen for the prevention of vertical transmission od HIV-infection on pregnant women in a French cohort	2016	
862	Exclude	wrong study design	Scientific Abstracts	2016	
863	Exclude	wrong outcome	Scientific challenges in early pregnancy achievement	2016	
864	Exclude	wrong outcome	Single dose dipyrone (metamizole) for acute postoperative pain in	2016	
865	Exclude	wrong outcome	Single fixed-dose oral dexketoprofen plus tramadol for acute postoperative pain in adults	2016	
866	Exclude	wrong outcome	Solid lipid nanoparticles (SLN) -based hydrogel formulation for topical delivery of miconazole nitrate	2016	
867	Exclude	wrong outcome	Sonographic factors distinguishing late intrauterine growth restriction from late small for gestational age fetuses	2016	
868	Exclude	wrong population	Speaking valves in mechanically ventilated ICU patients-improved communication and improved lung recruitment	2016	
869	Exclude	wrong outcome	Stability of expression of related factors mRNA in cryopreserved human amniotic membrane	2016	
870	Exclude	wrong outcome	STRATEGIES IMPLEMENTED TO ENSURE KNOWLEDGE	2016	
871	Exclude	wrong outcome	Successful autotransplantation of cryopreserved ovarian tissue with recovery of the ovarian function	2016	
872	Exclude	wrong outcome	The application of serum cystatin C in estimating the renal function in women with severe preeclamptic toxemia	2016	
873	Exclude	wrong outcome	The association between ambient temperature and preterm birth in Shenzhen, China: a distributed lag non-linear time series analysis	2016	
874	Exclude	wrong outcome	The Betadine trial - antiseptic wound irrigation prior to skin closure at caesarean section to prevent surgical site infection: A randomised controlled trial	2016	

875	Exclude	wrong outcome	The Burden of Provider-Initiated Preterm Birth and Associated Factors: Evidence from the Brazilian Multicenter Study on Preterm	2016	
876	Exclude	wrong outcome	The determination of pregnancy results and type of birth using fetal renal artery doppler values in idiopathic oligohydramnios and polyhydramnios pregnancies	2016	
877	Exclude	registration	The Effect of the Timing of Colloid and Crystalloid Infusions on Postspinal Hypotension After Spinal Anesthesia for Caesarian	2016	
878	Exclude	wrong outcome	THE EMPIRICAL MANAGEMENT of the TOXEMIAS of	2016	
879	Exclude	wrong outcome	The evaluation of the fetal central hemodynamics using pulsed Doppler-ultrasound	2016	
880	Exclude	wrong outcome	The impact of intrahepatic cholestasis of pregnancy on fetal cardiac and peripheral circulation	2016	
881	Exclude	wrong outcome	The impact of placental insufficiency on fetal survival in twin-twin transfusion syndrome (TTTS) undergoing fetoscopic laser treatment	2016	
882	Exclude	wrong outcome	The Infant Microbiome: Implications for Infant Health and Neurocognitive Development	2016	
883	Exclude	wrong outcome	THE INFLUENCE OF PLACENTALPERFUSION ON PLACENTAL GLUCOSETRANSFER IN NORMAL AND DIABETICPREGNANCIES	2016	
884	Exclude	wrong population	The perfusion index of healthy term infants during transition at birth	2016	
885	Exclude	wrong outcome	The Probiotics in Pregnancy Study (PiP Study): rationale and design of a double-blind randomised controlled trial to improve maternal health during pregnancy and prevent infant eczema and allergy	2016	
886	Exclude	wrong outcome	The rate of blood-flow in the umbilical cord	2016	
887	Exclude	wrong outcome	The relationship between human placental morphometry and ultrasonic measurements of utero-placental blood flow and fetal	2016	
888	Exclude	wrong study design	THE RELATIONSHIP BETWEEN LUNG AERATION AND LUNGLIQUID REMOVAL IN NEWBORN RABBITS	2016	
889	Exclude	wrong population	The use of pulse wave transit time (PWTT) non-invasive cardiac output monitoring during labour epidural anaesthesia and subsequent fetal distress leading to emergency caesarean section	2016	
890	Exclude	wrong population	Thoracic stent graft versus surgery for thoracic aneurysm	2016	
891	Exclude	wrong population	Topical cystic fibrosis transmembrane conductance regulator gene replacement for cystic fibrosis-related lung disease	2016	
892	Exclude	wrong outcome	Topical NSAIDs for chronic musculoskeletal pain in adults	2016	
893	Exclude	wrong outcome	Transplacental transfer of 2- naphthol across the human placenta	2016	
894	Exclude	wrong study design	Transplantation Basic Science, Allogenic and Xenogenic Tolerance	2016	
895	Exclude	wrong outcome	Ultrasonic assessment of abnormal umbilical cord and its circulation	2016	

896	Exclude	wrong outcome	Umbilical artery, middle cerebral artery and uterine artery Doppler indices in pregnant women with hypertensive disorders and correlation with perinatal outcome	2016	
897	Exclude	wrong outcome	Upshaw-schulman syndrome revealed by a post partum complication; About a Tunisian patient	2016	
898	Exclude	wrong outcome	Use of cerebroplacental ratio to predict outcome in late onset fetal growth restriction (FGR)	2016	
899	Exclude	wrong outcome	Using the angiogenic factors sFlt-1 and PlGF with Doppler ultrasound of the uterine artery for confirming preeclampsia	2016	
900	Exclude	wrong outcome	Using the Peanut Exercise Ball to Reduce Cesarean Section Rates: a Randomized Controlled Trial	2016	
901	Exclude	wrong outcome	Uterine artery pulsatility index at 30-34 weeks' gestation in the prediction of adverse perinatal outcome	2016	
902	Exclude	wrong study design	Uterine myomatosis and intrauterine grouwth retardation. Myomectomy during cesarean: one case report	2016	
903	Exclude	wrong outcome	Utility of a data collection and analysis pilot system for the characterization of factors influencing the cesarean section rate: outcomes in complejo hospitalario de pontevedra (CHOP)	2016	
904	Exclude	wrong outcome	Validation of a predictive riskmodel for adverse perinataloutcome in late-onset small forgestational age fetuses	2016	
905	Exclude	wrong outcome	Value of third trimester uterine artery Doppler for prediction of adverse perinatal outcome in high risk pregnancies	2016	
906	Exclude	wrong outcome	VTE in pregnancy: How to assess, what to do	2016	
907	Exclude	wrong outcome	Work of breathing during CPAP and heated humidified high-flow nasal cannula	2016	
908	Exclude	wrong population	XI SOLANEP International Congress / XV Cystic Fibrosis Latinamerican Congress / XV Brazilian Congress of Pediatric	2016	
909	Exclude	wrong outcome	"You should go so that others can come"; the role of facilities in determining an early departure after childbirth in Morogoro Region, Tanzania	2015	
910	Exclude	wrong study design	[Congenital Intermittent Third-degree Atrioventricular Block Associated with Retinoid Exposure in Pregnancy]	2015	
911	Exclude	wrong publication type	33rd Congress of the Scandinavian Society of Anaesthesiology and Intensive Care Medicine	2015	
912	Exclude	wrong outcome	A national survey of obstetricians' attitudes toward and practice of periviable intervention	2015	
913	Exclude	wrong outcome	Alternative versus standard packages of antenatal care for low-risk pregnancy	2015	

914	Exclude	wrong outcome	An integrated model with classification criteria to predict small-for-gestational-age fetuses at risk of adverse perinatal outcome	2015	
915	Exclude	wrong outcome	Associations of maternal weight status prior and during pregnancy with neonatal cardiometabolic markers at birth: the Healthy Start	2015	
916	Exclude	wrong population	Budesonide for induction of remission in Crohn's disease	2015	
917	Exclude	wrong outcome	Cervical Ripening Balloon in Induction of Labour at Term	2015	
918	Exclude	no details of PI or PVI	Changes in pleth variability index and detection of hypotension during spinal anaesthesia for caesarean section	2015	
919	Exclude	wrong outcome	Chlorhexidine gluconate versus povidone iodine at cesarean delivery: a randomized controlled trial	2015	
920	Exclude	wrong outcome	Clinical utility of third-trimester uterine artery Doppler in the prediction of brain hemodynamic deterioration and adverse perinatal outcome in small-for-gestational-age fetuses	2015	
921	Exclude	wrong outcome	Comparing obstetricians' and neonatologists' approaches to periviable counseling	2015	
922	Exclude	wrong outcome	Comparison of Subcuticular Suture Materials in Cesarean Skin	2015	
923	Exclude	wrong outcome	Comparison of Umbilical Cord Milking and Delayed Cord Clamping on Cerebral Blood Flow in Term Neonates	2015	
924	Exclude	wrong index test	Continuous measurement of cardiac output with the electrical velocimetry method in patients under spinal anesthesia for cesarean	2015	
925	Exclude	wrong outcome	Diagnosis of Placenta Accreta by Uterine Artery Doppler Velocimetry in Patients With Placenta Previa	2015	
926	Exclude	wrong outcome	Effect of Pulsed Electromagnetic Field Therapy on Pain After Cesarean Delivery	2015	
927	Exclude	wrong outcome	Effects of total fat intake on body weight	2015	
928	Exclude	wrong outcome	Factors associated with increased cesarean risk among African American women: evidence from California, 2010	2015	
929	Exclude	wrong outcome	Fetal-maternal interface impedance parallels local NADPH oxidase related superoxide production	2015	
930	Exclude	wrong outcome	FUNCTIONAL CHANGES OF THE PELVIC FLOOR MUSCLES FOLLOWING VAGINAL DELIVERY: THE EFFECT OF ETHNICITY ANDAVULSION INJURY	2015	
931	Exclude	wrong outcome	Glucose and Fatty Acid Metabolism in Placental Explants From Pregnancies Complicated With Gestational Diabetes Mellitus	2015	
932	Exclude	wrong outcome	Hemodynamic analysis in an idealized artery tree: differences in wall shear stress between Newtonian and non-Newtonian blood models	2015	
933	Exclude	wrong outcome	Identification of early environmental risk factors for irritable bowel syndrome and dyspepsia	2015	

934	Exclude	wrong study design	Inhibition of intimal hyperplasia via local delivery of vascular endothelial growth factor cDNA nanoparticles in a rabbit model of restenosis induced by abdominal aorta balloon injury	2015	
935	Exclude	wrong population	Interventions for hirsutism (excluding laser and photoepilation therapy alone)	2015	
936	Exclude	wrong outcome	Labor and Delivery Experiences of Mothers with Suspected Large	2015	
937	Exclude	wrong study design	Low Abdominal NIRS Values and Elevated Plasma Intestinal Fatty Acid-Binding Protein in a Premature Piglet Model of Necrotizing	2015	
938	Exclude	wrong outcome	Maternal vitamin D status, prolonged labor, cesarean delivery and instrumental delivery in an era with a low cesarean rate	2015	
939	Exclude	wrong outcome	Maternal, Infant Characteristics, Breastfeeding Techniques, and Initiation: Structural Equation Modeling Approaches	2015	
940	Exclude	wrong outcome	Measurement of middle cerebral artery diameter as a method for assessment of brain sparing in intra-uterine growth-restricted discordant twins	2015	
941	Exclude	wrong outcome	Morbidity and mortality associated with mode of delivery for breech periviable deliveries	2015	
942	Exclude	wrong outcome	Multiple BRAF Wild-Type Melanomas During Dabrafenib Treatment for Metastatic BRAF-Mutant Melanoma	2015	
943	Exclude	wrong population	Nutritional support in children and young people with cancer undergoing chemotherapy	2015	
944	Exclude	review article	Offspring of parents with chronic pain: a systematic review and meta-analysis of pain, health, psychological, and family outcomes	2015	
945	Exclude	wrong population	Parenteral fluid regimens for improving functional outcome in people with acute stroke	2015	
946	Exclude	wrong population	Perfusion Index is Increased in Acute Complex Regional Pain Syndrome Type 1	2015	
947	Exclude	wrong outcome	Perinatal outcome ofgrowth restrictedfetuses after iatrogenic pretermdeliverysecondarytoearlyonset severepreeclampsia with abnormal dopplers: The UHWI experience	2015	
948	Exclude	wrong population	Perinatal risk factors increase the risk of being affected by both type 1 diabetes and coeliac disease	2015	
949	Exclude	wrong population	Perioperative Systemic Lidocaine for Enhanced Bowel Recovery After Bariatric Surgery	2015	
950	Exclude	wrong population	Pilot Randomized Controlled Trial of Internet-Delivered Cognitive-Behavioral Treatment for Pediatric Headache	2015	
951	Exclude	duplicate	Pleth valiability index is a reflection of perfusion index and not fluid status for caesarean section under regional anaesthesia	2015	

952	Exclude	wrong outcome	Prediction of small-for-gestational-age neonates: screening by uterine artery Doppler and mean arterial pressure at 19-24 weeks	2015	
953	Exclude	wrong outcome	Prediction of small-for-gestational-age neonates: screening by uterine artery Doppler and mean arterial pressure at 30-34 weeks	2015	
954	Exclude	wrong outcome	Prediction of small-for-gestational-age neonates: screening by uterine artery Doppler and mean arterial pressure at 35-37 weeks	2015	
955	Exclude	wrong outcome	Prophylactic Incisional Care in Obese Women at Cesarean	2015	
956	Exclude	wrong population	Psychological interventions for parents of children and adolescents with chronic illness	2015	
957	Exclude	wrong outcome	Quantitative analysis of tightness of nuchal cord and its relationship with fetal intrauterine distress	2015	
958	Exclude	wrong outcome	Randomized Controlled Trial on the Effect of Channa striatus Extract on Measurement of the Uterus, Pulsatility Index, Resistive Index of Uterine Artery and Superficial Skin Wound Artery in Post Lower Segment Caesarean Section Women	2015	
959	Exclude	wrong population	Safety and Efficacy of Distending Media Infusion by Manual Syringe Method for Hysteroscopic Procedures: A Retrospective Analysis	2015	
960	Exclude	wrong population	Safety and efficacy of manual syringe infusion of distending media for hysteroscopic procedures: a case-control study	2015	
961	Exclude	wrong outcome	Single dose oral diclofenac for acute postoperative pain in adults	2015	
962	Exclude	wrong outcome	Single dose oral ibuprofen plus caffeine for acute postoperative pain in adults	2015	
963	Exclude	wrong outcome	Single dose oral ibuprofen plus codeine for acute postoperative pain in adults	2015	
964	Exclude	wrong outcome	Stress, sleep quality and unplanned Caesarean section in pregnant	2015	
965	Exclude	wrong outcome	Subcuticular Suture for Cesarean Skin Incision Closure	2015	
966	Exclude	wrong outcome	The association between pentraxin 3 in maternal circulation and pathological intrauterine fetal growth restriction	2015	
967	Exclude	wrong outcome	The Effect of Birth Plans on Obstetrical Outcomes	2015	
968	Exclude	registration	The effects of fluid loading on the accuracy of noninvasive hemoglobin monitoring in patients undergoing Cesarean section	2015	
969	Exclude	wrong outcome	The effects of high perioperative inspiratory oxygen fraction for adult surgical patients	2015	
970	Exclude	wrong outcome	The influence of resuscitation preferences on obstetrical management of periviable deliveries	2015	
971	Include		The predictive ability of non-invasive haemodynamic parameters for hypotension during caesarean section: a prospective observational	2015	https://doi.org/10.1111/anae.12992
972	Exclude	wrong outcome	The value of uterine artery Doppler and NT-proBNP levels in the second trimester to predict preeclampsia	2015	

973	Exclude	wrong outcome	Timing of elective delivery in gastroschisis: a decision and cost-effectiveness analysis	2015	
974	Exclude	wrong outcome	Topical NSAIDs for acute musculoskeletal pain in adults	2015	
975	Exclude	wrong outcome	Triage for HSIL+/CIN2+ women in type 16- or/and 18-positive primary HPV-based screening using cytology p16/Ki67 test: A pilot study	2015	
976	Exclude	wrong outcome	Umbilical and fetal middle cerebral artery Doppler at 30-34 weeks' gestation in the prediction of adverse perinatal outcome	2015	
977	Exclude	wrong outcome	Umbilical and fetal middle cerebral artery Doppler at 35-37 weeks' gestation in the prediction of adverse perinatal outcome	2015	
978	Exclude	wrong outcome	Uterine artery impedance during puerperium in normotensive and chronic hypertensive pregnant women	2015	
979	Exclude	wrong outcome	A study to find the better method to assess the neck of the womb before artificially starting the process of child birth by inserting a drug called 嵏塵inoprostone嵏・(prostaglandin) through the birth passage	2014	
980	Exclude	wrong outcome	Abstracts of the XXIV European Congress of Perinatal Medicine, June 4-7, 2014, Florence, Italy	2014	
981	Exclude	wrong outcome	Ambulatory blood pressure measurements in mid-pregnancy and development of hypertensive pregnancy disorders	2014	
982	Exclude	wrong outcome	Association of Doppler parameters with placental signs of underperfusion in late-onset small-for-gestational-age pregnancies	2014	
983	Exclude	wrong outcome	Audio spectrum analysis of umbilical artery Doppler ultrasound signals applied to a clinical material	2014	
984	Exclude	wrong outcome	Blood flow to the scarred gravid uterus at 22-24 weeks of gestation	2014	
985	Exclude	wrong population	Budesonide for maintenance of remission in Crohn's disease	2014	
986	Exclude	wrong outcome	CENTRAL NERVOUS SYSTEMDISEASES FOLLOWINGEXTREMELY PRETERM BIRTH:OPTIMAL DELIVERY	2014	
987	Exclude	wrong outcome	Challenges to measuring variation in readmission rates of neonatal intensive care patients	2014	
988	Exclude	wrong population	Changes in cerebral oxygenation during early postnatal adaptation in newborns delivered by vacuum extraction measured by near-infrared spectroscopy	2014	
989	Exclude	wrong outcome	Changes in the sublingual microcirculation during major abdominal surgery and post-operative morbidity	2014	
990	Exclude	wrong outcome	Chlorhexidine-Alcohol Versus Povidone-Iodine for Cesarean	2014	
991	Exclude	wrong outcome	Cohort profile update: 2004 Pelotas (Brazil) Birth Cohort Study. Body composition, mental health and genetic assessment at the 6 years	2014	
992	Exclude	wrong outcome	Comparison between placental gene expression of 11beta-hydroxysteroid dehydrogenases and infantile growth at 10 months of	2014	

993	Exclude	wrong outcome	Comparison of perinatal outcome in growth restricted fetuses retaining normal umbilical artery Doppler flow to those with diminished end-diastolic flow	2014	
994	Exclude	wrong outcome	Correlation between First and Second Trimester Uterine Artery Doppler Velocimetry and Placental Bed Histopathology	2014	
995	Exclude	wrong outcome	Effect of high-flow nasal cannula on thoraco-abdominal synchrony in adult critically ill patients	2014	
996	Exclude	wrong outcome	Effect of povidone iodine on thyroid functions and urine iodine levels in caesarean operations	2014	
997	Exclude	wrong outcome	Effects of Beta-blockade on Cardiopulmonary Exercise Testing	2014	
998	Exclude	wrong outcome	Examination of intrarenal resistance indices indicate the involvement of renal pathology as a significant diagnostic classifier of	2014	
999	Exclude	wrong outcome	Fetal middle cerebral and umbilical artery Doppler after 40 weeks gestational age	2014	
1000	Exclude	wrong outcome	Fetal subcutaneous tissue measurements in pregnancy as a predictor of neonatal total body composition	2014	
1001	Exclude	wrong study design	Functional assessment of hyperoxia-induced lung injury after preterm birth in the rabbit	2014	
1002	Exclude	duplicate	Heart rate, but not heart rate variability or pulse oximetry parameters of perfusion, predicts hypotension during spinal anesthesia for a cesarean section: A prospective observational study	2014	
1003	Exclude	wrong outcome	Hemodynamic changes in the femoral vein with increasing outflow resistance	2014	
1004	Exclude	wrong outcome	Impact of a third stage of labor oxytocin protocol on cesarean delivery outcomes	2014	
1005	Exclude	wrong outcome	Impact of obesity on maternal and neonatal outcomes in insulin-resistant pregnancy	2014	
1006	Exclude	wrong outcome	Induction of interferon-gamma and downstream pathways during establishment of fetal persistent infection with bovine viral diarrhea	2014	
1007	Exclude	wrong outcome	Maternal hemodynamics by thoracic impedance cardiography for normal pregnancy and the postpartum period	2014	
1008	Exclude	wrong population	Methods of intraperitoneal local anaesthetic instillation for laparoscopic cholecystectomy	2014	
1009	Exclude	wrong outcome	Mode of delivery and postpartum HIV-1 disease progression and mortality in a Kenyan cohort	2014	
1010	Exclude	wrong population	Neuraminidase inhibitors for preventing and treating influenza in adults and children	2014	
1011	Exclude	wrong outcome	Neuraxial blockade for the prevention of postoperative mortality and major morbidity: an overview of Cochrane systematic reviews	2014	

1012	Exclude	wrong outcome	New Approaches to Treatment of Severe Intrauterine Growth	2014	
1013	Exclude	wrong population	Nocturnal mechanical ventilation for chronic hypoventilation in patients with neuromuscular and chest wall disorders	2014	
1014	Exclude	wrong outcome	Not only hysterectomy but also cesarean section can predict incomplete flexible sigmoidoscopy among patients with prior abdominal or pelvic surgery	2014	
1015	Exclude	wrong outcome	Obstetric care and method of delivery in Mexico: results from the 2012 National Health and Nutrition Survey	2014	
1016	Exclude	wrong outcome	Optimizing Management of the 2nd Stage of Labor: multicenter Randomized Trial	2014	
1017	Exclude	wrong outcome	Perioperative and transfusion outcomes in women undergoing cesarean hysterectomy for abnormal placentation	2014	
1018	Exclude	wrong study design	Phenylephrine infusion on maternal placental perfusion changes during spinal anesthesia for elective cesarean delivery	2014	
1019	Exclude	wrong outcome	Physiological comparison of breathing patterns with neurally adjusted ventilatory assist (NAVA) and pressure-support ventilation to improve NAVA settings	2014	
1020	Exclude	wrong outcome	Placental growth factor for the prediction of fetal outcomes in pregnancies at risk and in IUGR	2014	
1021	Exclude	wrong outcome	Prevention of Surgical Site Infection After Cesarean Delivery	2014	
1022	Exclude	wrong outcome	Rare earth element and SrNd isotope geochemistry of phosphate nodules from the lower Cambrian Niutitang Formation, NW Hunan Province, South China	2014	
1023	Exclude	wrong study design	Regulation of amino acid transporters by adenoviral-mediated human insulin-like growth factor-1 in a mouse model of placental insufficiency in vivo and the human trophoblast line BeWo in vitro	2014	
1024	Include		Role of pleth variability index for predicting hypotension after spinal anesthesia for cesarean section	2014	https://doi.org/10.1016/j.ijoa.2014.05.011
1025	Exclude	wrong outcome	Second-trimester prediction of delivery of a small-for-gestational-age neonate: integrating sequential Doppler information, fetal biometry, and maternal characteristics	2014	
1026	Exclude	wrong population	Single dose oral etoricoxib for acute postoperative pain in adults	2014	
1027	Exclude	wrong population	The difference between standing and sitting in 3 different seat inclinations on abdominal muscle activity and chest and abdominal expansion in woodwind and brass musicians	2014	
1028	Exclude	wrong index test	The effect of neuraxial anesthesia on maternal cerebral	2014	
1029	Exclude	wrong outcome	The median effective seated time for hypotension induced by spinal anesthesia at Cesarean delivery with two doses of hyperbaric bupivacaine: a randomized up-down sequential allocation study	2014	

1030	Exclude	wrong outcome	The use of postpartum hemorrhage protocols in United States academic obstetric anesthesia units	2014	
1031	Exclude	wrong study design	Therapy with prostaglandins in the early postpartum period	2014	
1032	Exclude	wrong outcome	Tranexamic Acid in Adherent Placenta (TAP)	2014	
1033	Exclude	wrong outcome	Trans-placental passage and anti-inflammatory effects of solithromycin in the human placenta	2014	
1034	Exclude	wrong study design	Vagal anandamide signaling via cannabinoid receptor 1 contributes to luminal 5-HT modulation of visceral nociception in rats	2014	
1035	Exclude	wrong study design	Validation of the gastroschisis experimental model and the influence of the mother's diet enriched with glutamine in the fetal morphology	2014	
1036	Exclude	wrong population	Web-based Management of Pediatric Functional Abdominal Pain	2014	
1037	Exclude	wrong outcome	Yogic Breathing Changes Salivary Components	2014	
1038	Exclude	wrong outcome	[Effect of high altitude on birth weight and adverse perinatal outcomes in two Argentine populations]	2013	
1039	Exclude	wrong population	[Guidelines for perioperative haemodynamic optimization. Socie te franc aise d'anesthe sie et de re animation]	2013	
1040	Exclude	wrong outcome	Analysis of obstetrics and gynecology professional liability claims in Catalonia, Spain (1986-2010)	2013	
1041	Exclude	wrong outcome	Applicability of fetal renal artery Doppler values in determining pregnancy outcome and type of delivery in idiopathic oligohydramnios and polyhydramnios pregnancies	2013	
1042	Exclude	wrong population	Aspirin with or without an antiemetic for acute migraine headaches in adults	2013	
1043	Exclude	wrong outcome	Association between first trimester vaginal bleeding and uterine artery Doppler measured at second and third trimesters of pregnancy	2013	
1044	Exclude	wrong population	Desferrioxamine mesylate for managing transfusional iron overload in people with transfusion-dependent thalassaemia	2013	
1045	Exclude	wrong outcome	Diclofenac with or without an antiemetic for acute migraine headaches in adults	2013	
1046	Exclude	wrong outcome	Disproportionate body composition and neonatal outcome in offspring of mothers with and without gestational diabetes mellitus	2013	
1047	Exclude	wrong outcome	Ductus venosus versus cerebral transverse sinus Doppler velocimetry for predicting acidemia at birth in pregnancies complicated by placental insufficiency	2013	
1048	Exclude	wrong outcome	Effect of continuous high-volume hemofiltration on patients with acute respiratory distress syndrome	2013	
1049	Exclude	wrong study design	Evaluation of Masimo signal extraction technology pulse oximetry in anaesthetized pregnant sheep	2013	

1050	Exclude	wrong outcome	Fetal heart rate and cardiotocographic abnormalities with varying dose misoprostol vaginal inserts	2013	
1051	Exclude	wrong outcome	Gestational weight gain in insulin-resistant pregnancies	2013	
1052	Exclude	wrong outcome	Hematologic profile of neonates with growth restriction is associated with rate and degree of prenatal Doppler deterioration	2013	
1053	Exclude	wrong outcome	Hemodynamic effects of vasopressor on uterine and placenta	2013	
1054	Exclude	wrong population	Ibuprofen with or without an antiemetic for acute migraine headaches in adults	2013	
1055	Exclude	wrong outcome	Improvement in uterine artery doppler indices via hysteroscopic metroplasty	2013	
1056	Exclude	wrong outcome	Increased cord blood angiotensin II concentration is associated with decreased insulin sensitivity in the offspring of mothers with gestational diabetes mellitus	2013	
1057	Exclude	wrong population	Naproxen with or without an antiemetic for acute migraine headaches in adults	2013	
1058	Exclude	wrong outcome	Obstetrical epidural and spinal anesthesia in multiple sclerosis	2013	
1059	Exclude	wrong population	Opioids for acute pancreatitis pain	2013	
1060	Exclude	wrong outcome	Paracetamol (acetaminophen) with or without an antiemetic for acute migraine headaches in adults	2013	
1061	Exclude	wrong population	Pegylated liposomal doxorubicin for relapsed epithelial ovarian	2013	
1062	Include		Perfusion index derived from a pulse oximeter can predict the incidence of hypotension during spinal anaesthesia for Caesarean	2013	https://doi.org/10.1093/bja/aet058
1063	Exclude	wrong outcome	Postpartum uterine artery Doppler velocimetry among patients following a delivery complicated with preeclampsia	2013	
1064	Exclude	duplicate	Pre-anesthetic PVI (Pleth Variability Index) predicts hypotension after spinal anesthesia during cesarean section	2013	
1065	Exclude	wrong outcome	Predicting cesarean in the second stage of labor	2013	
1066	Exclude	review article	Prevalence of placenta praevia by world region: a systematic review and meta-analysis	2013	
1067	Exclude	wrong outcome	Pulmonary artery catheters for adult patients in intensive care	2013	
1068	Exclude	wrong outcome	Respiratory flutter syndrome in a neonate	2013	
1069	Exclude	wrong outcome	Role of colour Doppler indices in the diagnosis of intrauterine growth retardation in high-risk pregnancies	2013	
1070	Exclude	wrong outcome	Single dose oral celecoxib for acute postoperative pain in adults	2013	
1071	Exclude	wrong outcome	Single dose oral dexibuprofen [S(+)-ibuprofen] for acute postoperative pain in adults	2013	
1072	Exclude	wrong outcome	Single dose oral ibuprofen plus oxycodone for acute postoperative pain in adults	2013	

1073	Exclude	wrong outcome	Single dose oral ibuprofen plus paracetamol (acetaminophen) for acute postoperative pain	2013	
1074	Exclude	wrong study design	Successful management of aortic dissection in a patient with Marfan syndrome during pregnancy	2013	
1075	Exclude	wrong outcome	Successful unrelated cord blood transplantation for homozygous alpha-thalassemia	2013	
1076	Exclude	wrong outcome	The diagnostics potential of integral rheography during the dynamic examination of body fluid sectors at puerperal women with	2013	
1077	Exclude	wrong outcome	The risks and benefits of internal monitors in laboring patients	2013	
1078	Exclude	wrong population	Transdermal fentanyl for cancer pain	2013	
1079	Exclude	wrong outcome	Value of Doppler sonography near term: can umbilical and uterine artery indices in low-risk pregnancies predict perinatal outcome?	2013	
1080	Exclude	wrong outcome	[Umbilical cord collision in the first trimester in a monoamniotic twin pregnancy--does it really matter?]	2012	
1081	Exclude	wrong outcome	An anthropometric model to estimate neonatal fat mass using air displacement plethysmography	2012	
1082	Exclude	wrong outcome	Aortocaval compression in Pregnancy	2012	
1083	Exclude	wrong study design	Blood oxygenation during hyperpressure intraperitoneal fluid administration in a rabbit model of severe liver injury: Evaluation of a novel concept for control of pre-hospital liver bleeding	2012	
1084	Exclude	wrong study design	Bovine viral diarrhea virus cyclically impairs long bone trabecular modeling in experimental persistently infected fetuses	2012	
1085	Exclude	wrong outcome	Comparison of low doses of intrathecal bupivacaine in combined spinal epidural anaesthesia with epidural volume extension for	2012	
1086	Exclude	wrong index test	Detection of hypotension during Caesarean section with continuous non-invasive arterial pressure device or intermittent oscillometric arterial pressure measurement	2012	
1087	Exclude	wrong outcome	Development of fetal and placental innate immune responses during establishment of persistent infection with bovine viral diarrhea virus	2012	
1088	Exclude	wrong outcome	Disproportionate body composition and perinatal outcome in large-for-gestational-age infants to mothers with type 1 diabetes	2012	
1089	Exclude	duplicate	Efficacy of chlorhexidine gluconate versus povidone iodine for skin disinfection at cesarean section: a randomized controlled trial	2012	
1090	Exclude	wrong outcome	Electrical velocimetry demonstrates the increase in cardiac output and decrease in systemic vascular resistance accompanying cesarean delivery and oxytocin administration	2012	
1091	Exclude	wrong outcome	Estimated weight centile as a predictor of perinatal outcome in small-for-gestational-age pregnancies with normal fetal and maternal	2012	

1092	Exclude	wrong outcome	Gastropexy using the Carter-Thomason device in lieu of T-fasteners in a critically ill, severely obese patient: an innovative technique	2012	
1093	Exclude	wrong study design	Neonatal neurobehavior and diffusion MRI changes in brain reorganization due to intrauterine growth restriction in a rabbit model	2012	
1094	Exclude	wrong outcome	Novel method for targeting fluid and vasopressor management in caesarean section under spinal anaesthesia using continuous non-invasive monitoring	2012	
1095	Exclude	review article	Pain management for women in labour: an overview of systematic	2012	
1096	Exclude	wrong outcome	Perinatal outcomes in women with multiple sclerosis exposed to disease-modifying drugs	2012	
1097	Exclude	wrong outcome	Perioperative increase in global blood flow to explicit defined goals and outcomes following surgery	2012	
1098	Exclude	wrong outcome	Pharmacoproteomic study of three different chondroitin sulfate compounds on intracellular and extracellular human chondrocyte	2012	
1099	Exclude	wrong population	Psychological interventions for parents of children and adolescents with chronic illness	2012	
1100	Exclude	wrong outcome	Pulmonary artery flow catheters for directing management in pre-eclampsia	2012	
1101	Exclude	wrong outcome	Pulmonary embolism in pregnancy. Consensus and controversies	2012	
1102	Exclude	wrong outcome	Resveratrol-loaded solid lipid nanoparticles versus nanostructured lipid carriers: evaluation of antioxidant potential for dermal	2012	
1103	Exclude	wrong outcome	Single dose oral aspirin for acute postoperative pain in adults	2012	
1104	Exclude	duplicate	Umbilical cord collision in the first trimester in a monoamniotic twin pregnancy - does it really matter?	2012	
1105	Exclude	wrong outcome	Warfarine in Unexplained Oligohydramnios	2012	
1106	Exclude	wrong outcome	What's new in obstetric anesthesia: the 2011 Gerard W. Ostheimer	2012	
1107	Exclude	wrong outcome	[Relationship between total bile acid concentration and fetal pulmonary surfactant in intrahepatic cholestasis of pregnancy]	2011	
1108	Exclude	wrong outcome	[Shortened hospital stay for elective cesarean section after initiation of a fast-track program and midwifery home-care]	2011	
1109	Exclude	wrong outcome	A 30-year review of advanced abdominal pregnancy at the Mater Misericordiae Hospital, Afikpo, southeastern Nigeria (1976-2006)	2011	
1110	Exclude	wrong outcome	Atazanavir in pregnancy: a report of 155 cases	2011	
1111	Exclude	wrong outcome	Comparing postcesarean infectious complication rates using two different skin preparations	2011	
1112	Exclude	wrong study design	Cytokines and acute phase proteins associated with acute swine influenza infection in pigs	2011	
1113	Exclude	wrong population	Doppler findings in intrapartum fetal distress	2011	

1114	Exclude	wrong outcome	Duration of persistent abnormal ductus venosus flow and its impact on perinatal outcome in fetal growth restriction	2011	
1115	Exclude	wrong index test	Effects of epidural analgesia on uterine artery Doppler in labour	2011	
1116	Exclude	wrong outcome	Electrical velocimetry elucidates the hemodynamics of hypertension caused by indigo carmine	2011	
1117	Exclude	wrong outcome	Fetal brain Doppler to predict cesarean delivery for nonreassuring fetal status in term small-for-gestational-age fetuses	2011	
1118	Exclude	wrong outcome	Fluid Management Study to Evaluate Changes in Intravascular Volume After Applying Various Pressure Levels on the Caval Vein	2011	
1119	Exclude	wrong study design	High positive end-expiratory pressure during high-frequency jet ventilation improves oxygenation and ventilation in preterm lambs	2011	
1120	Exclude	wrong study design	Impact on fetal mortality and cardiovascular Doppler of selective ligature of uteroplacental vessels compared with undernutrition in a rabbit model of intrauterine growth restriction	2011	
1121	Exclude	wrong outcome	Increased uterine artery pulsatility index at 34 weeks and outcome of pregnancy	2011	
1122	Exclude	wrong outcome	Induction maintenance concept for HAART as initial treatment in HIV infected infants	2011	
1123	Exclude	wrong study design	nha bífida em neonato felino: relato de caso	2011	
1124	Exclude	wrong outcome	Obstetrical and neonatal outcomes after prenatal exposure to selective serotonin reuptake inhibitors: the relevance of dose	2011	
1125	Exclude	wrong study design	Periurethral injection of autologous adipose-derived stem cells with controlled-release nerve growth factor for the treatment of stress urinary incontinence in a rat model	2011	
1126	Exclude	wrong outcome	Placental cord drainage after vaginal delivery as part of the management of the third stage of labour	2011	
1127	Exclude	wrong outcome	Postpartum depression and correlated factors in women who received in vitro fertilization treatment	2011	
1128	Exclude	wrong outcome	Predictive value of middle cerebral artery to uterine artery pulsatility index ratio in preeclampsia	2011	
1129	Exclude	wrong outcome	Probiotics for the prevention of gestational diabetes in overweight and obese women	2011	
1130	Exclude	wrong outcome	Regulator of calcineurin 1 mediates pathological vascular wall	2011	
1131	Exclude	wrong outcome	Republished review: An update on contraindications for lung function testing	2011	
1132	Exclude	wrong outcome	Single dose oral fenoprofen for acute postoperative pain in adults	2011	
1133	Exclude	wrong outcome	Single dose oral mefenamic acid for acute postoperative pain in	2011	
1134	Exclude	wrong study design	SP-A ADDITION TO EXOGENOUS SURFACTANT FOR RESPIRATORY DISTRESS SYNDROME IN PRETERM RABBITS	2011	

1135	Exclude	wrong outcome	Treatments for iron-deficiency anaemia in pregnancy	2011	
1136	Exclude	wrong population	Vaginal birth after cesarean for cephalopelvic disproportion: effect of birth weight difference on success	2011	
1137	Exclude	wrong outcome	A Comparison of Metallic Staples Versus Absorbable Staples After a Cesarean Section (C-Section)	2010	
1138	Exclude	wrong outcome	Antiretroviral therapy (ART) for treating HIV infection in ART-eligible pregnant women	2010	
1139	Exclude	wrong population	Birth-related factors and doctor-diagnosed wheezing and allergic sensitization in early childhood	2010	
1140	Exclude	wrong outcome	Can adverse neonatal outcome be predicted in late preterm or term fetal growth restriction?	2010	
1141	Exclude	wrong outcome	Central venous O saturation and venous-to-arterial CO difference as complementary tools for goal-directed therapy during high-risk	2010	
1142	Exclude	duplicate	Central venous O(2) saturation and venous-to-arterial CO(2) difference as complementary tools for goal-directed therapy during	2010	
1143	Exclude	wrong population	Complications of endografting: prevention and management	2010	
1144	Exclude	wrong population	Continuous subcutaneous insulin infusion (CSII) versus multiple insulin injections for type 1 diabetes mellitus	2010	
1145	Exclude	wrong population	Effect of mechanical ventilation on microvascular perfusion in critical care patients	2010	
1146	Exclude	wrong outcome	Evaluating risk-adjusted cesarean delivery rate as a measure of obstetric quality	2010	
1147	Exclude	wrong study design	Flexible split-ring electrode for insect flight biasing using multisite neural stimulation	2010	
1148	Exclude	wrong outcome	História reprodutiva de mulheres laqueadas	2010	
1149	Exclude	wrong outcome	Interventions for treating peripartum cardiomyopathy to improve outcomes for women and babies	2010	
1150	Exclude	wrong outcome	Intrapartum and postpartum analgesia for women maintained on buprenorphine during pregnancy	2010	
1151	Exclude	wrong population	Intravenous fluids for abdominal aortic surgery	2010	
1152	Exclude	wrong outcome	Intravenous immunoglobulin and necrotizing enterocolitis in newborns with hemolytic disease	2010	
1153	Exclude	wrong outcome	Lipid and cationic polymer based transduction of botulinum holotoxin, or toxin protease alone, extends the target cell range and improves the efficiency of intoxication	2010	
1154	Exclude	wrong outcome	Maternal haemodynamics at elective caesarean section: a randomised comparison of oxytocin 5-unit bolus and placebo infusion with oxytocin 5-unit bolus and 30-unit infusion	2010	

1155	Exclude	wrong population	Risk factors and presentations of periventricular venous infarction vs arterial presumed perinatal ischemic stroke	2010	
1156	Exclude	wrong outcome	Single dose oral codeine, as a single agent, for acute postoperative pain in adults	2010	
1157	Exclude	wrong outcome	Single dose oral diflunisal for acute postoperative pain in adults	2010	
1158	Exclude	wrong population	Single dose oral gabapentin for established acute postoperative pain in adults	2010	
1159	Exclude	wrong outcome	Techniques to assess tissue oxygenation in the clinical setting	2010	
1160	Exclude	wrong outcome	Unilaterally increased uterine artery resistance, placental location and pregnancy outcome	2010	
1161	Exclude	registration	Usefulness of pleth variability index to predict fluid responsiveness in patients undergoing Cesarean section	2010	
1162	Exclude	wrong outcome	Utero-placental Doppler ultrasound for improving pregnancy outcome	2010	
1163	Exclude	wrong outcome	[Relationship of fetal total bile acid and the change of fetal pancreas endocrine secretion and its impact on fetal growth and development in intrahepatic cholestasis of pregnancy]	2009	
1164	Exclude	wrong study design	Aberrant expression of myeloperoxidase in astrocytes promotes phospholipid oxidation and memory deficits in a mouse model of Alzheimer disease	2009	
1165	Exclude	wrong outcome	Agreement between umbilical vein volume blood flow measurements obtained at the intra-abdominal portion and free loop of the umbilical	2009	
1166	Exclude	wrong study design	An experimental model of fetal growth restriction based on selective ligature of uteroplacental vessels in the pregnant rabbit	2009	
1167	Exclude	wrong outcome	Cardiac troponin T as a biochemical marker of cardiac dysfunction and ductus venosus Doppler velocimetry	2009	
1168	Exclude	wrong outcome	Comparison of placental transfer of local anesthetics in perfusates with different pH values in a human cotyledon model	2009	
1169	Exclude	wrong outcome	Design of agonistic altered peptides for the robust induction of CTL directed towards H-2Db in complex with the melanoma-associated epitope gp100	2009	
1170	Exclude	wrong study design	Development of cell-mediated immunity to porcine circovirus type 2 (PCV2) in caesarean-derived, colostrum-deprived piglets	2009	
1171	Exclude	wrong outcome	Differential expression of the type I interferon pathway during persistent and transient bovine viral diarrhea virus infection	2009	
1172	Exclude	wrong population	Fetal growth parameters and birth weight: their relationship to neonatal body composition	2009	
1173	Exclude	wrong outcome	Hemodynamic effects of ephedrine, phenylephrine, and the coadministration of phenylephrine with oxytocin during spinal anesthesia for elective cesarean delivery	2009	

1174	Exclude	wrong outcome	Human T-cell leukemia virus type I tax down-regulates the expression of phosphatidylinositol 3,4,5-trisphosphate inositol phosphatases via the NF-kappaB pathway	2009	
1175	Exclude	wrong outcome	Inspiration regulates the rate and temporal pattern of lung liquid clearance and lung aeration at birth	2009	
1176	Exclude	wrong outcome	Intravenous or intramuscular parecoxib for acute postoperative pain in adults	2009	
1177	Exclude	wrong outcome	Lung tissue blood perfusion changes induced by in utero tracheal occlusion in a rabbit model of congenital diaphragmatic hernia	2009	
1178	Exclude	wrong outcome	MicroRNA expression, chromosomal alterations, and immunoglobulin variable heavy chain hypermutations in Mantle cell lymphomas	2009	
1179	Exclude	letter	More on perfusion index and its use as a maternal monitor for fetal well being	2009	
1180	Exclude	wrong outcome	Novel role for glutathione S-transferase pi. Regulator of protein S-Glutathionylation following oxidative and nitrosative stress	2009	
1181	Exclude	wrong study design	Nucleophosmin is cleaved and inactivated by the cytotoxic granule protease granzyme M during natural killer cell-mediated killing	2009	
1182	Exclude	wrong study design	Positive end-expiratory pressure enhances development of a functional residual capacity in preterm rabbits ventilated from birth	2009	
1183	Exclude	wrong outcome	Pregnancys-associated venous thromboembolism Part I - Deep vein thrombus diagnosis and treatment	2009	
1184	Exclude	wrong outcome	Problematic integration in pregnancy and childbirth: contrasting approaches to uncertainty and desire in obstetric and midwifery care	2009	
1185	Exclude	wrong population	Prostate cancer detected by methylated gene markers in histopathologically cancer-negative tissues from men with subsequent positive biopsies	2009	
1186	Exclude	wrong study design	Rab1 guanine nucleotide exchange factor SidM is a major phosphatidylinositol 4-phosphate-binding effector protein of Legionella pneumophila	2009	
1187	Exclude	wrong population	Randomized controlled trial of an Internet-delivered family cognitive-behavioral therapy intervention for children and adolescents with chronic pain	2009	
1188	Exclude	wrong outcome	Single dose oral aceclofenac for postoperative pain in adults	2009	
1189	Exclude	wrong outcome	Single dose oral acemetacin for acute postoperative pain in adults	2009	
1190	Exclude	wrong outcome	Single dose oral etodolac for acute postoperative pain in adults	2009	
1191	Exclude	wrong outcome	Single dose oral fenbufen for acute postoperative pain in adults	2009	
1192	Exclude	wrong population	Single dose oral flurbiprofen for acute postoperative pain in adults	2009	
1193	Exclude	wrong outcome	Single dose oral ibuprofen for acute postoperative pain in adults	2009	
1194	Exclude	wrong outcome	Single dose oral lornoxicam for acute postoperative pain in adults	2009	

1195	Exclude	wrong outcome	Single dose oral meloxicam for acute postoperative pain in adults	2009	
1196	Exclude	wrong outcome	Single dose oral nabumetone for acute postoperative pain in adults	2009	
1197	Exclude	wrong outcome	Single dose oral naproxen and naproxen sodium for acute postoperative pain in adults	2009	
1198	Exclude	wrong outcome	Single dose oral nefopam for acute postoperative pain in adults	2009	
1199	Exclude	wrong outcome	Single dose oral oxycodone and oxycodone plus paracetamol (acetaminophen) for acute postoperative pain in adults	2009	
1200	Exclude	wrong outcome	Single dose oral paracetamol (acetaminophen) with codeine for postoperative pain in adults	2009	
1201	Exclude	wrong population	Single dose oral rofecoxib for acute postoperative pain in adults	2009	
1202	Exclude	wrong outcome	Single dose oral sulindac for acute postoperative pain in adults	2009	
1203	Exclude	wrong outcome	Single dose oral tenoxicam for acute postoperative pain in adults	2009	
1204	Exclude	wrong outcome	Single dose oral tiaprofenic acid for acute postoperative pain in adults	2009	
1205	Exclude	wrong outcome	The cell death-inducing activity of the peptide containing Noxa mitochondrial-targeting domain is associated with calcium release	2009	
1206	Exclude	wrong study design	Tyrosine polyethylene glycol (PEG)-micelle magnetic resonance contrast agent for the detection of lipid rich areas in atherosclerotic	2009	
1207	Exclude	wrong outcome	A C-terminal sequence in the guanine nucleotide exchange factor Sec7 mediates Golgi association and interaction with the Rsp5	2008	
1208	Exclude	wrong outcome	A functionally important hydrogen-bonding network at the betaDP/alphaDP interface of ATP synthase	2008	
1209	Exclude	wrong outcome	Antioxidants for preventing pre-eclampsia	2008	
1210	Exclude	wrong outcome	Association between polymorphisms in DNA base excision repair genes XRCC1, APE1, and ADPRT and differentiated thyroid	2008	
1211	Exclude	wrong outcome	Bendamustine is effective in p53-deficient B-cell neoplasms and requires oxidative stress and caspase-independent signaling	2008	
1212	Exclude	wrong outcome	Catalytically active monomer of glutathione S-transferase pi and key residues involved in the electrostatic interaction between subunits	2008	
1213	Exclude	wrong study design	Dehydroepiandrosterone stimulates phosphorylation of FoxO1 in vascular endothelial cells via phosphatidylinositol 3-kinase- and protein kinase A-dependent signaling pathways to regulate ET-1	2008	
1214	Exclude	wrong outcome	Determinants of molecular specificity in phosphoinositide regulation. Phosphatidylinositol (4,5)-bisphosphate (PI(4,5)P2) is the endogenous lipid regulating TRPV1	2008	
1215	Exclude	wrong outcome	Determination of metformin transfer across the human placenta using a dually perfused ex vivo placental cotyledon model	2008	
1216	Exclude	wrong outcome	Dipeptidyl peptidase-4 (DPP-4) inhibitors for type 2 diabetes mellitus	2008	
1217	Exclude	wrong population	Do caregiving models after cesarean birth influence the infants' breathing adaptation and crying? A pilot study	2008	

1218	Exclude	wrong outcome	Doppler analysis and placental nitric oxide synthase expression during fetal growth restriction	2008	
1219	Exclude	wrong study design	Effects intracerebral microinjection and intraperitoneal injection of [60]fullerene on brain functions differ in rats	2008	
1220	Exclude	wrong population	Elevated expression of glutathione S-transferase pi and p53 confers poor prognosis in head and neck cancer patients treated with chemoradiotherapy but not radiotherapy alone	2008	
1221	Exclude	wrong population	Focal adhesion kinase controls aggressive phenotype of androgen-independent prostate cancer	2008	
1222	Exclude	wrong outcome	Focal gains of VEGFA and molecular classification of hepatocellular carcinoma	2008	
1223	Exclude	wrong outcome	Gab2 is involved in differential phosphoinositide 3-kinase signaling by two splice forms of c-Kit	2008	
1224	Exclude	wrong outcome	Glycogen synthase kinase 3beta is a novel regulator of high glucose- and high insulin-induced extracellular matrix protein synthesis in renal proximal tubular epithelial cells	2008	
1225	Exclude	wrong outcome	Identification of dopamine D1-D3 receptor heteromers. Indications for a role of synergistic D1-D3 receptor interactions in the striatum	2008	
1226	Exclude	wrong outcome	Impaired protein aggregate handling and clearance underlie the pathogenesis of p97/VCP-associated disease	2008	
1227	Exclude	wrong outcome	Increased rate of prematurity associated with antenatal antiretroviral therapy in a German/Austrian cohort of HIV-1-infected women	2008	
1228	Exclude	wrong study design	Interleukin (IL) 1beta induction of IL-6 is mediated by a novel phosphatidylinositol 3-kinase-dependent AKT/IkappaB kinase alpha pathway targeting activator protein-1	2008	
1229	Include		Maternal pulse oximetry perfusion index as a predictor of early adverse respiratory neonatal outcome after elective cesarean delivery	2008	https://doi.org/10.1097/PCC.0b013e3181670021
1230	Exclude	wrong outcome	Maternal-neonatal erythrocyte membrane Na(+), K (+)-ATPase and Mg (2+)-ATPase activities in relation to the mode of delivery	2008	
1231	Exclude	wrong outcome	Multiple mechanisms are responsible for transactivation of the epidermal growth factor receptor in mammary epithelial cells	2008	
1232	Exclude	wrong outcome	Nonmitochondrial ATP/ADP transporters accept phosphate as third substrate	2008	
1233	Exclude	wrong outcome	Overcoming glutathione S-transferase P1-related cisplatin resistance in osteosarcoma	2008	
1234	Exclude	wrong outcome	Pivotal role of Bcl-2 family proteins in the regulation of chondrocyte apoptosis	2008	

1235	Exclude	wrong outcome	Placental overexpression of transforming growth factor-beta3 in the HELLP syndrome	2008	
1236	Exclude	wrong study design	Prenatal diagnosis of dextrotransposition of the great arteries	2008	
1237	Exclude	wrong outcome	Progression of Doppler abnormalities in intrauterine growth restriction	2008	
1238	Exclude	wrong outcome	Protein kinase A regulates 3-phosphatidylinositide dynamics during platelet-derived growth factor-induced membrane ruffling and	2008	
1239	Exclude	wrong study design	Protein-tyrosine phosphatase alpha regulates stem cell factor-dependent c-Kit activation and migration of mast cells	2008	
1240	Exclude	wrong outcome	Requirement of inducible nitric-oxide synthase in lipopolysaccharide-mediated Src induction and macrophage migration	2008	
1241	Exclude	wrong population	Selective activation of muscle and skin nociceptors does not trigger exaggerated sympathetic responses in spinal-injured subjects	2008	
1242	Exclude	wrong outcome	Single dose oral paracetamol (acetaminophen) for postoperative pain in adults	2008	
1243	Exclude	wrong outcome	The mitogen-activated protein kinase phosphatase vaccinia H1-related protein inhibits apoptosis in prostate cancer cells and is overexpressed in prostate cancer	2008	
1244	Exclude	wrong outcome	The stability of the ternary interferon-receptor complex rather than the affinity to the individual subunits dictates differential biological	2008	
1245	Exclude	wrong outcome	Transgenic, fluorescent Leishmania mexicana allow direct analysis of the proteome of intracellular amastigotes	2008	
1246	Exclude	wrong outcome	Umbilical cord prolapse in Kaduna, northern Nigeria: a study of	2008	
1247	Exclude	wrong outcome	Unraveling molecular complexity of phosphorylated human cardiac troponin I by top down electron capture dissociation/electron transfer dissociation mass spectrometry	2008	
1248	Exclude	wrong outcome	Variations in fibrinolytic parameters and inhibin-A in pregnancy: related hypertensive disorders	2008	
1249	Exclude	wrong study design	Birth asphyxia during general anesthesia for a morbidly obese parturient with gestational diabetes mellitus undergoing cesarean section--a case report	2007	
1250	Exclude	wrong outcome	Computerized fetal heart rate analysis, Doppler ultrasound and biophysical profile score in the prediction of acid-base status of growth-restricted fetuses	2007	
1251	Exclude	wrong outcome	Efficacy of low-dose boosted saquinavir once daily plus nucleoside reverse transcriptase inhibitors in pregnant HIV-1-infected women with a therapeutic drug monitoring strategy	2007	
1252	Exclude	wrong population	Exercise for treating fibromyalgia syndrome	2007	

1253	Exclude	wrong outcome	Prevention of HIV1 Mother to Child Transmission Without Nucleoside Analogue Reverse Transcriptase Inhibitors in the Pre-partum Phase. ANRS 135 Primeva	2007	
1254	Exclude	wrong outcome	[What does fetal renal echogenicity mean in intrauterine growth retardation?]	2006	
1255	Exclude	wrong outcome	A certain Caesarean (Caesar)	2006	
1256	Exclude	wrong index test	Assessment of pulse transit time to indicate cardiovascular changes during obstetric spinal anaesthesia	2006	
1257	Exclude	wrong outcome	Chinese herbal medicine for the treatment of pre-eclampsia	2006	
1258	Exclude	wrong outcome	Doppler and biophysical assessment in growth restricted fetuses: distribution of test results	2006	
1259	Exclude	wrong outcome	Effects of epinephrine, norepinephrine, and phenylephrine on microcirculatory blood flow in the gastrointestinal tract in sepsis	2006	
1260	Exclude	wrong outcome	Evaluation of third trimester uterine artery flow velocity indices in relationship to perinatal complications	2006	
1261	Exclude	wrong outcome	Fetal middle cerebral to uterine artery pulsatility index ratios in normal and pre-eclamptic pregnancies	2006	
1262	Exclude	wrong population	Hemodynamic patterns of blunt and penetrating injuries	2006	
1263	Exclude	wrong outcome	Lactacidemia in intrauterine growth restricted (IUGR) pregnancies: relationship to clinical severity, oxygenation and placental weight	2006	
1264	Exclude	wrong index test	Maternal haemodynamics in pre-eclampsia compared with normal pregnancy during caesarean delivery	2006	
1265	Exclude	wrong outcome	Placental transfer and pharmacokinetics of lopinavir and other protease inhibitors in combination with nevirapine at delivery	2006	
1266	Exclude	wrong outcome	Prediction of intrapartum Cesarean delivery for non-reassuring fetal status after a successful external cephalic version by a low pre-version pulsatility index of the fetal middle cerebral artery	2006	
1267	Exclude	wrong outcome	Rest during pregnancy for preventing pre-eclampsia and its complications in women with normal blood pressure	2006	
1268	Exclude	wrong outcome	[Pulsed Doppler measurements of flow velocities in the intracranial and abdominal arteries of newborns of diabetic mothers]	2005	
1269	Exclude	wrong population	[The prediction of the condition of newborns on the basis of antepartum CTG monitoring and antepartum doppler flow velocity in umbilical arteries in women delivered by caesarean section]	2005	
1270	Exclude	wrong outcome	A pilot-controlled study of a polymyxin B-immobilized hemoperfusion cartridge in patients with severe sepsis secondary to intra-abdominal infection	2005	
1271	Exclude	wrong population	Chinese medical herbs for chemotherapy side effects in colorectal cancer patients	2005	

1272	Exclude	wrong population	Effects of escharotomy as abdominal decompression on cardiopulmonary function and visceral perfusion in abdominal compartment syndrome with burn patients	2005	
1273	Exclude	wrong outcome	Epidural local anesthetics: a novel treatment for fetal growth	2005	
1274	Exclude	wrong outcome	Factors affecting umbilical venous perfusion during experimental cord knotting	2005	
1275	Exclude	wrong outcome	Increased uterine artery vascular impedance is related to adverse outcome of pregnancy but is present in only one-third of late third-trimester pre-eclamptic women	2005	
1276	Exclude	wrong outcome	Influence of expiratory flow-limitation during exercise on systemic oxygen delivery in humans	2005	
1277	Exclude	wrong population	Long-term non-pharmacologic weight loss interventions for adults with type 2 diabetes	2005	
1278	Exclude	wrong index test	Maternal hemodynamics during cesarean delivery assessed by whole-body impedance cardiography	2005	
1279	Exclude	wrong outcome	Mid-trimester placentation assessment in high-risk pregnancies using maternal serum screening and uterine artery Doppler	2005	
1280	Exclude	wrong population	Pharmacotherapy for weight loss in adults with type 2 diabetes	2005	
1281	Exclude	wrong outcome	Placental expression of nitric oxide synthase during HELLP syndrome: the correlation with maternal-fetal Doppler velocimetry	2005	
1282	Exclude	wrong outcome	Preterm birth contributes to increased vascular resistance and higher blood pressure in adolescent girls	2005	
1283	Exclude	wrong outcome	Prognostic value of umbilical-middle cerebral artery pulsatility index ratio in fetuses with growth restriction	2005	
1284	Exclude	wrong outcome	Protease inhibitor use in 233 pregnancies	2005	
1285	Exclude	wrong outcome	[Clinical analysis of pregnancy complicated by hypertension in the material of the Department of Obstetrics and Perinatology of the Pomeranian Academy of Medicine]	2004	
1286	Exclude	wrong outcome	Abnormal maternal cardiac function precedes the clinical manifestation of fetal growth restriction	2004	
1287	Exclude	wrong outcome	Assessment of fetal cerebral arterial and venous blood flow before and after vaginal delivery or Cesarean section	2004	
1288	Exclude	wrong population	Breast-feeding problems after epidural analgesia for labour: a retrospective cohort study of pain, obstetrical procedures and breast-	2004	
1289	Exclude	duplicate	Characteristics of porcine circovirus-2 replication in lymphoid organs of pigs inoculated in late gestation or postnatally and possible relation to clinical and pathological outcome of infection	2004	
1290	Exclude	wrong outcome	Clinical significance of uterine artery blood flow velocity waveforms during provoked uterine contractions in high-risk pregnancy	2004	

1291	Exclude	wrong outcome	Cost-effectiveness of antenatal HIV-testing: reviewing its pharmaceutical and methodological aspects	2004	
1292	Exclude	wrong outcome	Estimates of risk of venous thrombosis during pregnancy and puerperium are not influenced by diagnostic suspicion and referral	2004	
1293	Exclude	wrong study design	Involvement of the peripheral cholinergic muscarinic system in the compensatory ovarian hypertrophy in the rat	2004	
1294	Exclude	wrong outcome	Spectral Doppler index mapping of the umbilicoplacental circulation and pregnancy outcome	2004	
1295	Exclude	wrong outcome	Transfer of inflammatory cytokines across the placenta	2004	
1296	Exclude	wrong outcome	Utero-ovarian morphology and blood flow after tubal ligation by the Pomeroy technique	2004	
1297	Exclude	wrong outcome	[Evolution of indications for cesarean section between 1991 and 2000 in materials from the Pathology Clinic in the Department of Pregnancy and Labor, Pomeranian Medical University in Szczecin]	2003	
1298	Exclude	wrong outcome	[Intensive care management of 28 patients with severe eclampsia in a tropical African setting]	2003	
1299	Exclude	wrong outcome	[Longitudinal analysis of arterial Doppler parameters in growth retarded fetuses]	2003	
1300	Exclude	wrong study design	[Prenatal and postnatal cardiologic evaluation of normal twin from acardiac twin pregnancy--case report]	2003	
1301	Exclude	wrong outcome	Clinical significance of normalization of uterine artery pulsatility index with maternal heart rate for the evaluation of uterine circulation in pregnancy-induced hypertension	2003	
1302	Exclude	wrong study design	Combination treatment with an ET(A)-receptor blocker and an ACE inhibitor is not superior to the respective monotherapies in attenuating chronic transplant vasculopathy in different aorta allotransplantation rat models	2003	
1303	Exclude	wrong outcome	Deep venous thrombosis during pregnancy and after delivery: indications for and results of thrombectomy	2003	
1304	Exclude	wrong outcome	Doppler-Sonographiescreening der A. uterina in der gynakologischen Praxis	2003	
1305	Exclude	wrong outcome	Effect of antiretroviral therapy on pregnancy outcome in HIV-1 positive women	2003	
1306	Exclude	wrong outcome	Elevated maternal mid-trimester chorionic gonadotropin > or =4 MoM is associated with fetal cerebral blood flow redistribution	2003	
1307	Exclude	duplicate	Elevated maternal mid-trimester chorionic gonadotropin >=4 MoM is associated with fetal cerebral blood flow redistribution	2003	

1308	Exclude	wrong study design	Experimental reproduction of postweaning multisystemic wasting syndrome in cesarean-derived, colostrum-deprived piglets inoculated with porcine circovirus type 2 (PCV2): investigation of quantitative PCV2 distribution and antibody responses	2003	
1309	Exclude	wrong outcome	Is Doppler prediction of anemia effective in the growth-restricted	2003	
1310	Exclude	wrong outcome	New score indicating placental vascular resistance	2003	
1311	Exclude	wrong study design	Septicaemia and arthritis in pigs experimentally infected with Pasteurella multocida capsular serotype A	2003	
1312	Exclude	wrong population	Supranormal trauma resuscitation causes more cases of abdominal compartment syndrome	2003	
1313	Exclude	wrong outcome	[Mother-infant and indirect transmission of HSV infection: treatment and prevention]	2002	
1314	Exclude	wrong outcome	Increasing maternal blood pressure with ephedrine increases uterine artery blood flow velocity during uterine contraction	2002	
1315	Exclude	wrong outcome	Management of thromboembolic disease in pregnancy	2002	
1316	Exclude	wrong outcome	Maternal hydration for increasing amniotic fluid volume in oligohydramnios and normal amniotic fluid volume	2002	
1317	Exclude	wrong outcome	Prognostic value of uterine artery Doppler velocimetry in growth-restricted fetuses delivered near term	2002	
1318	Exclude	wrong population	Prone positioning, systemic hemodynamics, hepatic indocyanine green kinetics, and gastric intramucosal energy balance in patients with acute lung injury	2002	
1319	Exclude	wrong outcome	S100B protein cord blood levels and development of fetal behavioral states: a study in normal and small-for-dates fetuses	2002	
1320	Exclude	wrong population	Short acting beta agonists for recurrent wheeze in children under 2 years of age	2002	
1321	Exclude	wrong outcome	[Para/endocrine function of the vascular endothelium of healthy pregnant women and pregnant women with preeclampsia and their	2001	
1322	Exclude	wrong outcome	Accuracy of single ultrasound parameters in detection of fetal growth restriction	2001	
1323	Exclude	wrong outcome	Characterization of growth-restricted fetuses with breakdown of the brain-sparing effect diagnosed by spectral Doppler	2001	
1324	Exclude	wrong outcome	Dopexamine hydrochloride does not modify hemodynamic response or tissue oxygenation or gut permeability during abdominal aortic	2001	
1325	Exclude	wrong outcome	Doppler velocimetry and thrombophilic screening at middle trimester of gestation: preliminary data	2001	
1326	Exclude	wrong population	Effect of airway opening manoeuvres on thoraco-abdominal asynchrony in anaesthetized children	2001	
1327	Exclude	wrong outcome	Fetal thoracic aorta doppler in cases with intrauterine growth	2001	

1328	Exclude	wrong population	Iliac arterial injuries after endovascular repair of abdominal aortic aneurysms: correlation with iliac curvature and diameter	2001	
1329	Exclude	wrong outcome	Longitudinal observation of deterioration of Doppler parameters, computerized cardiotocogram and clinical course in a fetus with growth restriction	2001	
1330	Exclude	wrong population	Lung recruitment and breathing pattern during variable versus continuous flow nasal continuous positive airway pressure in premature infants: an evaluation of three devices	2001	
1331	Exclude	wrong study design	Maturation of baseline breathing and of hypercapnic and hypoxic ventilatory responses in newborn mice	2001	
1332	Exclude	wrong outcome	Modulation of potassium current characteristics in human myometrial smooth muscle by 17beta-estradiol and progesterone	2001	
1333	Exclude	wrong population	Physostigmine for Alzheimer's disease	2001	
1334	Exclude	wrong outcome	Placental and fetal pulsatility indices in gestational diabetes mellitus	2001	
1335	Exclude	wrong population	The effects of prone positioning on intraabdominal pressure and cardiovascular and renal function in patients with acute lung injury	2001	
1336	Exclude	wrong outcome	Umbilical artery pulsatility index in pregnancies complicated by insulin-dependent diabetes mellitus without hypertension	2001	
1337	Exclude	wrong population	Ventilatory responses to rebreathing in infants exposed to maternal smoking	2001	
1338	Exclude	wrong study design	Characterization of immune response of young pigs to porcine circovirus type 2 infection	2000	
1339	Exclude	wrong outcome	Combination antiretroviral therapy and duration of pregnancy	2000	
1340	Exclude	wrong outcome	Combination antiretroviral therapy and duration of pregnancy	2000	
1341	Exclude	wrong outcome	c-Sections of Maximal Subgroups of Finite Groups	2000	
1342	Exclude	wrong outcome	Ex vivo human placental transfer and the vasoactive properties of hydralazine	2000	
1343	Exclude	wrong outcome	Ex vivo human placental transfer of trovafloxacin	2000	
1344	Exclude	wrong outcome	Expression of protein kinase C isozymes in nonpregnant and pregnant human myometrium	2000	
1345	Exclude	wrong outcome	Fetal renal artery flow and renal echogenicity in the chronically hypoxic state	2000	
1346	Exclude	wrong outcome	On C-section and C-index of finite groups	2000	
1347	Exclude	wrong outcome	Permeation studies comparing cobra skin with human skin using nicotine transdermal patches	2000	
1348	Exclude	retracted	RETRACTED: Influence of dopexamine on hemodynamics, intramucosal pH, and regulators of the macrocirculation and microcirculation in patients undergoing abdominal aortic surgery	2000	

1349	Exclude	wrong population	Technetium-99m labelled macroaggregated albumin arterial catheter perfusion scintigraphy: prediction of gastrointestinal toxicity in hepatic arterial chemotherapy	2000	
1350	Exclude	wrong outcome	Transfer of methohexital across the perfused human placenta	2000	
1351	Exclude	wrong outcome	Umbilical vein blood flow in growth-restricted fetuses	2000	
1352	Exclude	wrong population	[Acute effect of dobutamine and amrinone on hemodynamics and splanchnic perfusion in septic shock patients]	1999	
1353	Exclude	wrong outcome	[Clinical observation of misoprostol on induction in late pregnancy]	1999	
1354	Exclude	wrong population	[Micronized progesterone in the treatment of imminent necrosis of a myoma during pregnancy. Ultrasound changes during treatment]	1999	
1355	Exclude	wrong outcome	19-year follow-up of fetal myelomeningocele brought to term	1999	
1356	Exclude	wrong outcome	Autologous transfusion in obstetrics	1999	
1357	Exclude	wrong outcome	Clinical pharmacokinetics of the newer neuromuscular blocking drugs	1999	
1358	Exclude	wrong population	Effects of different modes of delivery on lung volumes of newborn	1999	
1359	Exclude	wrong outcome	Erfassung der fetalen Retardierung mittels Ponderal Index und Gewichtsperzentilen	1999	
1360	Exclude	wrong outcome	Evaluation of cerebral perfusion pressure changes in laboring women: effects of epidural anesthesia	1999	
1361	Exclude	wrong outcome	German-Austrian Guidelines for HIV-therapy during pregnancy--status: May/June 1998--common statement of the Deutsche AIDS-Gesellschaft (DAIG) and the Osterreichische AIDS-Gesellschaft	1999	
1362	Exclude	wrong outcome	Impaired glucose transport and insulin receptor tyrosine phosphorylation in skeletal muscle from obese women with	1999	
1363	Exclude	wrong outcome	Incidence, clinical characteristics, and timing of objectively diagnosed venous thromboembolism during pregnancy	1999	
1364	Exclude	wrong outcome	Minilaparoscopy to reduce complications from cannula insertion in patients with previous pelvic or abdominal surgery	1999	
1365	Exclude	wrong outcome	Prognostic markers in twin pregnancies with an acardiac fetus	1999	
1366	Exclude	wrong population	Splanchnic circulation and regional sympathetic outflow during peroperative PEEP ventilation in humans	1999	
1367	Exclude	wrong outcome	ST-segment depression and myocardial contractility during cesarean section under spinal anesthesia	1999	
1368	Exclude	wrong study design	The dynamics of glomerular filtration after Caesarean section	1999	
1369	Exclude	wrong outcome	[The study on relationship between fetal blood flow velocity waveforms and cord blood gas analyses in normal full term	1998	
1370	Exclude	wrong population	Amnioinfusion in labor induction of term pregnancies with premature rupture of the membranes and low amniotic fluid	1998	
1371	Exclude	wrong outcome	Effect of nimesulide and indomethacin on contractility and the Ca2+ channel current in myometrial smooth muscle from pregnant women	1998	

1372	Exclude	wrong outcome	Incidence, clinical characteristics, and timing of objectively diagnosed venous thromboembolism during pregnancy	1998	
1373	Exclude	wrong study design	Lung function and bacterial proliferation in experimental neonatal pneumonia in ventilated rabbits exposed to monoclonal antibody to surfactant protein A	1998	
1374	Exclude	wrong population	Oxygen delivery is an important predictor of outcome in patients with ruptured abdominal aortic aneurysms	1998	
1375	Exclude	retracted	Retracted: Influence of dopexamine hydrochloride on haemodynamics and regulators of circulation in patients undergoing	1998	
1376	Exclude	wrong outcome	Superior mesenteric artery flow velocity waveforms in small for gestational age fetuses	1998	
1377	Exclude	wrong outcome	Uterine and umbilical artery velocimetry in pre-eclampsia	1998	
1378	Exclude	wrong population	Very low birthweight infants: outcome in a sub-Arctic population	1998	
1379	Exclude	wrong outcome	[Fetal doppler flow velocimetry measurements as indications for cesarean section]	1997	
1380	Exclude	wrong population	[Patients with early stages of endometrial cancer should be spared adjuvant radiotherapy. Danish Endometrial Cancer Group]	1997	
1381	Exclude	wrong outcome	[Puerperal infection. Analysis of 618 cases]	1997	
1382	Exclude	wrong outcome	[Small-volume resuscitation for hypovolemic shock. Concept, experimental and clinical results]	1997	
1383	Exclude	wrong study design	A porcine model for the evaluation of virulence of Bordetella	1997	
1384	Exclude	wrong outcome	Influence of epidural analgesia on the labor induced by premature rupture of membranes	1997	
1385	Exclude	wrong population	Intravenous nicardipine in the treatment of postoperative arterial hypertension	1997	
1386	Exclude	wrong population	Long-term follow-up of hemostatic molecular markers during remission induction therapy with all-trans retinoic acid for acute promyelocytic leukemia. Keio Hematology-Oncology Cooperative	1997	
1387	Exclude	wrong outcome	Patient-controlled epidural analgesia after caesarean section using meperidine	1997	
1388	Exclude	wrong outcome	Sufentanil transfer in the human placenta during in vitro perfusion	1997	
1389	Exclude	wrong outcome	The maternal-fetal transfer of lamivudine in the ex vivo human	1997	
1390	Exclude	wrong outcome	[Level of cortisol in blood serum of maternal and umbilical cord during various methods of delivery]	1996	
1391	Exclude	wrong outcome	[Value of pulsatile Doppler ultrasound in diagnosis of suspected placental insufficiency in comparison with other examination	1996	
1392	Exclude	wrong population	Adrenal artery velocity waveforms in the appropriate and small-for-gestational-age fetus	1996	

1393	Exclude	wrong outcome	Antibodies to endometrial transferrin and alpha 2-Heremans Schmidt (HS) glycoprotein in patients with endometriosis	1996	
1394	Exclude	duplicate	Aortic, pulmonary, and ductal peak velocities in symmetrical and asymmetrical fetal growth retardation: A different adaptation to placental insufficiency	1996	
1395	Exclude	wrong outcome	Doppler velocimetry of different sections of the fetal middle cerebral artery in relation to perinatal outcome	1996	
1396	Exclude	wrong study design	Foetal supraventricular tachycardia and cerebral complications	1996	
1397	Exclude	wrong study design	Impaired ventilatory responses to hypoxia and hypercapnia in mutant mice deficient in endothelin-1	1996	
1398	Exclude	wrong outcome	Is the fetal brain-sparing effect a risk factor for the development of intraventricular hemorrhage in the preterm infant?	1996	
1399	Exclude	wrong index test	Maternal and uteroplacental haemodynamic state in pre-eclamptic patients during spinal anaesthesia for Caesarean section	1996	
1400	Exclude	wrong outcome	Mode of delivery and perinatal cerebral blood flow	1996	
1401	Exclude	wrong outcome	Prediction of perinatal outcome by middle cerebral artery Doppler velocimetry	1996	
1402	Exclude	wrong outcome	Randomized trial of bolus phenylephrine or ephedrine for maintenance of arterial pressure during spinal anaesthesia for	1996	
1403	Exclude	wrong outcome	The effects of varying volumes of crystalloid administration before cesarean delivery on maternal hemodynamics and colloid osmotic pressure	1996	
1404	Exclude	wrong population	Cardiopulmonary Hazards of Perihepatic Packing for Major Liver	1995	
1405	Exclude	wrong outcome	Early uterine artery Doppler velocimetry and the outcome of pregnancy in women aged 35 years and older	1995	
1406	Exclude	wrong index test	Effect of crystalloid and colloid preloading on uteroplacental and maternal haemodynamic state during spinal anaesthesia for	1995	
1407	Exclude	wrong study design	Ex vivo human placental transfer of human immunodeficiency virus-1 p24 antigen	1995	
1408	Exclude	wrong outcome	Experimental reproduction of Haemophilus parasuis infection in swine: clinical, bacteriological, and morphologic findings	1995	
1409	Exclude	wrong population	Gastric mucosal pH and oxygen delivery and oxygen consumption indices in the assessment of adequacy of resuscitation after trauma: a prospective, randomized study	1995	
1410	Exclude	wrong outcome	Interleukin-1 stimulates prostaglandin E production by human trophoblast cells from first and third trimesters	1995	
1411	Exclude	wrong study design	Maternal death following epidural anaesthesia for caesarean section delivery in a patient with unsuspected sepsis	1995	

1412	Exclude	wrong outcome	The effects of epidural ropivacaine and bupivacaine for cesarean section on uteroplacental and fetal circulation	1995	
1413	Exclude	wrong outcome	The human myometrium expresses multiple isoforms of the corticotropin-releasing hormone receptor	1995	
1414	Exclude	wrong outcome	Uptake of halothane and isoflurane by mother and baby during caesarean section	1995	
1415	Exclude	wrong population	A double-blind, placebo-controlled trial of transdermal fentanyl after abdominal hysterectomy. Analgesic, respiratory, and pharmacokinetic effects	1994	
1416	Exclude	wrong outcome	Amino acids, glucose and lactate concentrations in umbilical cord blood in relation to umbilical artery flow patterns	1994	
1417	Exclude	wrong population	Cardiovascular changes during continuous hyperthermic peritoneal perfusion	1994	
1418	Exclude	wrong outcome	Color Doppler evaluation of uteroplacentofetal circulation in management of high risk pregnancies	1994	
1419	Exclude	wrong population	Epidural sufentanil does not attenuate the central haemodynamic effects of caesarean section performed under epidural anaesthesia	1994	
1420	Exclude	wrong outcome	Intravenous administration of the proton pump inhibitor omeprazole reduces the risk of acid aspiration at emergency cesarean section	1994	
1421	Exclude	duplicate	INTRAVENOUS ADMINISTRATION OF THE PROTON PUMP INHIBITOR OMEPRAZOLE REDUCES THE RISK OF ACID ASPIRATION AT EMERGENCY CESAREAN-SECTION	1994	
1422	Exclude	wrong outcome	The effects of regional anaesthesia for caesarean section on maternal and fetal blood flow velocities measured by Doppler	1994	
1423	Exclude	wrong outcome	Transfer and uptake of alfentanil in the human placenta during in vitro perfusion	1994	
1424	Exclude	wrong outcome	Uterine and umbilical blood flow velocity during epidural anaesthesia for caesarean section	1994	
1425	Exclude	wrong outcome	Uteroplacental haemodynamics during spinal anaesthesia for caesarean section with two types of uterine displacement	1994	
1426	Exclude	wrong outcome	A high uterine artery pulsatility index reflects a defective development of placental bed spiral arteries in pregnancies complicated by hypertension and fetal growth retardation	1993	
1427	Exclude	wrong study design	An infant with macrocephaly, abnormal neuronal migration and persistent olfactory ventricles	1993	
1428	Exclude	wrong outcome	Fetal cerebral Doppler in the recognition of fetal compromise	1993	
1429	Exclude	wrong outcome	Intravenous ranitidine reduces the risk of acid aspiration of gastric contents at emergency cesarean section	1993	

1430	Exclude	duplicate	INTRAVENOUS RANITIDINE REDUCES THE RISK OF ACID ASPIRATION OF GASTRIC CONTENTS AT EMERGENCY CESAREAN-SECTION	1993	
1431	Exclude	wrong outcome	[Nosocomial infection during puerperium]	1992	
1432	Exclude	wrong outcome	RELATIONSHIP BETWEEN UMBILICAL ARTERY PULSATILITY INDEX AND FETAL PLATELET COUNT	1992	
1433	Exclude	wrong outcome	Survival prospects of extremely preterm infants: a 10-year experience in a single perinatal center	1992	
1434	Exclude	wrong outcome	[Doppler ultrasound of the umbilical artery of the dystrophic child]	1991	
1435	Exclude	wrong outcome	[Perioperative prevention of thrombosis in cesarean section: results of a randomized prospective comparative study with 6% hydroxyethyl starch and 0.62 low dose heparin]	1991	
1436	Exclude	wrong outcome	Development of respiratory chemoreflexes in response to alternations of fractional inspired oxygen in the newborn infant	1991	
1437	Exclude	wrong study design	Dose effects of antenatal corticosteroids for induction of lung maturation in preterm rabbits	1991	
1438	Exclude	wrong index test	Measurement of transthoracic electrical impedance	1991	
1439	Exclude	duplicate	PERIOPERATIVE THROMBOSIS PROPHYLAXIS - RESULTS OF A RANDOMIZED, PROSPECTIVE, COMPARATIVE-STUDY WITH HYDROXYETHYL STARCH-6-PERCENT 0.62 AND LOW-DOSE	1991	
1440	Exclude	wrong outcome	Umbilical artery flow velocity waveforms and cord blood viscosity	1991	
1441	Exclude	wrong outcome	[Evaluation of the central hemodynamics of the fetus using pulsed Doppler ultrasound]	1990	
1442	Exclude	wrong population	[Passive compliance of the total respiratory system in newborn infants born by cesarean section]	1990	
1443	Exclude	wrong outcome	A comparison of epidural and intramuscular morphine in patients following cesarean section	1990	
1444	Exclude	wrong study design	Development of the arterial chemoreflex and turnover of carotid body catecholamines in the newborn rat	1990	
1445	Exclude	wrong outcome	Lactate metabolism in normal and growth-retarded human fetuses	1990	
1446	Exclude	wrong population	Neonatal patterns of breathing after cesarean section with or without epidural fentanyl	1990	
1447	Exclude	duplicate	PASSIVE COMPLIANCE OF THE TOTAL RESPIRATORY SYSTEM IN NEWBORNS AFTER CESAREAN-SECTION	1990	
1448	Exclude	wrong outcome	[Flowmetry of the fetal renal artery--a possibility for improving the assessment of fetal circulatory centralization]	1989	
1449	Exclude	wrong outcome	[Measuring fetal vascular resistance with the Duplex scanner--a new fetal stress test]	1989	
1450	Exclude	wrong outcome	Recording maternal vaginal apex pulse	1989	

1451	Exclude	wrong study design	Recovery of treatment doses of surfactants from the lungs and vascular compartments of mechanically ventilated premature rabbits	1989	
1452	Exclude	wrong study design	Rib cage vs. abdominal displacement in dogs during forced oscillation to 32 Hz	1989	
1453	Exclude	wrong outcome	Thrombectomy of acute iliofemoral venous thrombosis during	1989	
1454	Exclude	wrong outcome	Umbilical artery flow velocity waveforms in labour	1989	
1455	Exclude	wrong outcome	[Comparison of the clinical value of 2 frequently used indices of fetal blood flow analysis (pulsatility index PI and resistance index RI)]	1988	
1456	Exclude	wrong outcome	A new experimental approach for the study of cardiopulmonary physiology during early development	1988	
1457	Exclude	wrong outcome	[Pathophysiologic and clinical aspects of measuring blood flow in utero-placental vessels, the umbilical artery, the fetal aorta and the fetal common carotid artery]	1987	
1458	Exclude	wrong outcome	Longitudinal determination of cerebral blood flow velocity in neonates with the Doppler technique	1987	
1459	Exclude	wrong study design	Rib cage versus abdominal displacement in rabbits during forced oscillations to 30 Hz	1987	
1460	Exclude	wrong outcome	The effect of epidural anaesthesia for caesarean section on maternal uterine and fetal umbilical artery blood flow velocity waveforms	1987	
1461	Exclude	wrong outcome	[Studies of coagulation-fibrinolysis system and cold insoluble globulin in cases of caesarean section]	1985	
1462	Exclude	wrong population	Intrauterine growth retardation (IUGR) in pre-term infants	1985	
1463	Exclude	wrong study design	The effects of pregnancy and parturition on phosphorus metabolites in rat uterus studied by 31P nuclear magnetic resonance	1985	
1464	Exclude	wrong outcome	[Use of integral rheography of the body in obstetrics for the quantitative evaluation of cardiac output]	1984	
1465	Exclude	wrong outcome	Sympathoadrenal activity and peripheral blood flow after birth: comparison in infants delivered vaginally and by cesarean section	1984	
1466	Exclude	wrong population	[Characteristic features of systemic hemodynamics during cesarean section under general anesthesia with ketamine]	1983	
1467	Exclude	wrong outcome	Deep vein thrombosis during pregnancy. A prospective study	1983	
1468	Exclude	wrong population	Measurement of cardiac stroke volume during cesarean section: a comparison between impedance cardiography and the dye dilution technique	1983	
1469	Exclude	wrong population	Postnatal changes of cerebral blood flow velocity in normal term	1983	
1470	Exclude	wrong study design	Immunologic responses of the bovine fetus to parvovirus infection	1982	
1471	Exclude	wrong outcome	Premature labor. I. Prostaglandin precursors in human placental membranes	1981	
1472	Exclude	wrong population	Urinary loss of oxypurines in hypoxic premature neonates	1980	

1473	Exclude	wrong outcome	[Fetal movements and high-risk pregnancy]	1979	
1474	Exclude	wrong outcome	Acute deep vein thrombosis (DVT) after cesarean section	1979	
1475	Exclude	wrong index test	Measurements of cardiac stroke volume in various body positions in pregnancy and during Caesarean section: a comparison between thermodilution and impedance cardiography	1979	
1476	Exclude	wrong population	Serial measurements of thoracic impedance and cardiac output in healthy neonates after normal delivery and caesarean section	1979	
1477	Exclude	wrong population	Effects of delivery by caesarean section on lung mechanics and lung volume in the human neonate	1978	
1478	Exclude	wrong outcome	Crying vital capacity. Measurement of neonatal lung function	1976	
1479	Exclude	wrong outcome	The influence of lateral tilt on limb blood flow in advanced pregnancy	1976	
1480	Exclude	wrong population	Generalized pulmonary hyperinflation and Fallot's tetralogy in a neonate investigated by pulmonary physiological and radioisotopic	1975	
1481	Exclude	wrong study design	Immunologic response of the bovine fetus to inactivated infectious bovine rhinotracheitis-infectious pustular vulvovaginitis virus	1973	
1482	Exclude	wrong outcome	[Sphygmologic examination of cardiovascular dynamics during cesarean section--with special reference to autotransfusion-like effects during delivery]	1971	
1483	Exclude	wrong outcome	Aortocaval compression during cesarean section. A cause of newborn depression	1971	
1484	Exclude	wrong outcome	Prevention of hyalin-membrane disease in the term cesarean-section	1961	
