# Supplementary material for: Ability of pulse oximetry-derived indices to predict hypotension after spinal anesthesia for cesarean delivery: A systematic review and meta-analysis
Source: PLoS One. 2025 Jan 31;20(1):e0316715. doi: 10.1371/journal.pone.0316715 (PMC11785266; doi:10.1371/journal.pone.0316715)
Supplement: S2 Table — (DOCX) [file pone.0316715.s002.docx]

**S2 Table.** The characteristic of the studies included in this systematic review

| First Author | Year | Study design | N | Index test | Cut-off value | Ref | Definition of hypotension | Eligibility criteria | Anaesthesia method | Drugs used for spinal anaesthesia | Funding / COI |
| --- | --- | --- | --- | --- | --- | --- | --- | --- | --- | --- | --- |
| Arslan  [28] | 2019 | Prospective observational study | 87 | PI/PVI | -/- | NIBP | SBP < 80 mmHg or presence of symptoms consistent with hypotension (such as dizziness, nausea, and vomiting) | Elective CS  Age 18–40 years Singleton pregnancy | Spinal | 10 mg of 0.5% hyperbaric bupivacaine | None |
| De Felice  [32] | 2008 | Prospective observational study | 44 | PI | 1.9 | - | Systolic pressure reduction from baseline ≥ 30% or< 90 mmHg lasting ≥ 3 min | Elective CS at term gestation with no known risk factors | Spinal or CSEA or GA | 10 mg of 0.5% hyperbaric bupivacaine with 0.2 mg of morphine sulphate | None |
| Duggappa  [30] | 2017 | Prospective observational study | 120 | PI | 3.5 | NIBP | MAP < 65 mmHg | Elective CS Age 20–35 years | Spinal | 10 mg of 0.5% hyperbaric bupivacaine | None |
| Harde  [15] | 2023 | Prospective observational study | 90 | PI | 3.5 | NIBP | MAP < 65 mmHg | Elective CS  Age 20–35 years ASA-PS II  Spinal anaesthesia with a T6 level | Spinal | 10 mg of 0.5% hyperbaric bupivacaine with 20 micrograms of fentanyl | None |
| Inamanamelluri  [33] | 2022 | Prospective observational study | 50 | PI | 2.85 | NIBP | MAP < 20% of baseline or < 60 mmHg | Elective CS  ASA-PS II | Spinal | 10 mg of 0.5% hyperbaric bupivacaine | None |
| Jabarulla  [12] | 2022 | Prospective observational study | 63 | PI | 1.75 | NIBP | Decrease in SBP > 25% from the baseline | Elective CS  Age 20–35 years | Spinal | 10 mg of 0.5% hyperbaric bupivacaine with 20 micrograms of fentanyl | None |
| Kuwata  [11] | 2018 | Prospective observational study | 50 | PI/PVI | 3.1/18 | NIBP | SBP < 90 mmHg or < 80% of the baseline value | Elective CS | Spinal | 10 mg of 0.5% hyperbaric bupivacaine and fentanyl 10 microgram | None |
| Lal  [25] | 2022 | Prospective observational study | 60 | PI | 3.5 | NIBP | Decrease in SBP > 20% from baseline or < 80 mmHg | Elective CS ASA-PS I and II Age 18–35 years Singleton pregnancy | Spinal | 10 mg of 0.5% hyperbaric bupivacaine | None |
| Nandini  [24] | 2022 | Prospective observational study | 51 | PI | 3.5 | NIBP | Decrease in SBP > 25% | Elective CS ASA-PS II Singleton pregnancy Weight between 40 and 70 kg Height between 140 and 165 cm | Spinal | 10 mg of 0.5% hyperbaric bupivacaine | None |
| Ozyurt  [26] | 2022 | Prospective observational study | 44 | PVI | 18.5 | NIBP | 20% decrease in SBP as compared with their basal values or SBP < 80 mm Hg | Elective CS Age 18–40 years | Spinal | 10 mg of 0.5% hyperbaric bupivacaine | None |
| Patel  [27] | 2020 | Prospective observational study | 52 | PI | 4 | - | - | Elective CS ASA-PS I and II | Spinal | 12.5 mg of 0.5% hyperbaric bupivacaine | - |
| Pyakurel  [35] | 2020 | Prospective observational study | 68 | PI | 3.5 | - | Decrease in MAP by ≥ 20% from baseline value | Elective CS   Term parturients | Spinal | 10 mg of 0.5% hyperbaric bupivacaine with 20 micrograms of fentanyl | None |
| Shah  [36] | 2021 | Prospective observational study | 60 | PI | 3.5 | NIBP | MAP < 65 mmHg | Elective LSCS ASA grade I and II Age 20–35 years Gestational age between 36-41 weeks Healthy parturients | Spinal | 10 mg of 0.5% hyperbaric bupivacaine | - |
| Steffi  [34] | 2020 | Prospective observational study | 60 | PI | 3.5 | NIBP | Decrease in SBP > 20% of baseline | Elective CS ASA-PS II Age 20–35 years Singleton pregnancy Height 145–165 cm | Spinal | 10 mg of 0.5% hyperbaric bupivacaine | - |
| Sun  [31] | 2014 | Prospective observational study | 82 | PVI | 23.5 | NIBP | SBP < 80% of baseline | Elective CS Age 18–40 years Singleton pregnancy | CSEA | 10 mg of 0.5% isobaric bupivacaine was diluted with CSF to 2.5 mL | None |
| Thapa  [37] | 2022 | Prospective observational study | 246 | PI | 3.5 | NIBP | MAP of > 20％ from the baseline | Elective CS Age 18–35 years Singleton pregnancy Height 150–160 cm Baseline systolic blood pressure within 90–140 mmHg and baseline diastolic blood pressure within 60–90 mmHg | Spinal | 12 mg of 0.5％ hyperbaric bupivacaine | None |
| Toyama  [8] | 2013 | Prospective observational study | 35 | PI | 3.5 | NIBP | Decrease in SAP 25% from baseline | Elective CS | CSEA | 10 mg of 0.5% hyperbaric bupivacaine and fentanyl 20 microgram | None |
| Xu  [29] | 2017 | Prospective observational study | 94 | PI | 2.2 | NIBP | SBP < 80 mmHg or complaints of symptoms consistent with hypotension (e.g., faintness, dizziness, breathlessness, nausea, or vomiting) even without SBP < 80 mm Hg | Elective CS ASA-PS I-II Age 19–39 years Singleton pregnancy Full-term parturients | CSEA | 15 mg of 0.75% ropivacaine was diluted with cerebrospinal fluid to 3.0 mL, after which approximately 2.4 mL was injected | Sponsored by the Science and Technology Support Program from the Science and Technology Commission of Shanghai Municipality, the interdisciplinary Program of Shanghai Jiao Tong University, and the National Natural Science Foundation of China / None |
| Yokose  [7] | 2015 | Prospective observational study | 81 | PI/PVI | (3.5)/19 | NIBP | SBP < 80 mmHg | Elective CS | CSEA | 10 mg of 0.5% hyperbaric bupivacaine and 10 microgram fentanyl | None |
|  |  |  |  |  |  |  |  |  |  |  |  |
|  |  |  |  |  |  |  |  |  |  |  |  |
| N: total number of patients, Ref: reference standard, COI: Conflicts of interest, PI: perfusion index, PVI: pleth variability index, NIBP: non-invasive blood pressure, SBP: systolic blood pressure, MAP: mean arterial pressure, CS caesarean section, ASA-PS: American Society of Anesthesiology Physical Status, BMI; body mass index, CSEA: combined spinal-epidural anaesthesia, SAP: systolic arterial pressure, GA: general anaesthesia | | | | | | | | | | | |
| (Data obtained by contacting the authors) | | |  |  |  |  |  |  |  |  |  |
