# Supplementary material for: Ability of pulse oximetry-derived indices to predict hypotension after spinal anesthesia for cesarean delivery: A systematic review and meta-analysis
Source: PLoS One. 2025 Jan 31;20(1):e0316715. doi: 10.1371/journal.pone.0316715 (PMC11785266; doi:10.1371/journal.pone.0316715)
Supplement: S3 Table — CI; confidence interval, AUC; area under curve, sROC; summary receiver operating characteristic curve. (DOCX) [file pone.0316715.s003.docx]

**S3 Table.** 　Summary estimates of sensitivity, specificity, AUC of the sROC curve, positive likelihood ratio, and negative likelihood ratio of sensitivity analysis for perfusion index and pleth variability index

|  | Number of patients (study) | Sensitivity  (95% CI) | Specificity  (95% CI) | AUC of sROC  (95% CI) | Positive likelihood ratio  (95% CI) | Negative likelihood ratio  (95% CI) | I^2^ |
| --- | --- | --- | --- | --- | --- | --- | --- |
| perfusion index | 677(9) | 0.73  (0.64 to 0.80) | 0.58  (0.35 to 0.78) | 0.73  (0.61 to 0.80) | 1.86  (1.11 to 3.33) | 0.49  (0.31 to 0.80) | 40.1% |
| pleth variability index | 294 (4) | 0.59  (0.42 to 0.74) | 0.77  (0.64 to 0.86) | 0.75  (0.60 to 0.82) | 2.56  (1.75 to 3.71) | 0.54  (0.37 to 0.72) | 0.0% |

CI; confidence interval, AUC; area under curve, sROC; summary receiver operating characteristic curve
