# Supplementary material for: Ability of pulse oximetry-derived indices to predict hypotension after spinal anesthesia for cesarean delivery: A systematic review and meta-analysis
Source: PLoS One. 2025 Jan 31;20(1):e0316715. doi: 10.1371/journal.pone.0316715 (PMC11785266; doi:10.1371/journal.pone.0316715)
Supplement: S4 Table — CI; confidence interval, AUC; area under curve, sROC; summary receiver operating characteristic curve. (DOCX) [file pone.0316715.s004.docx]

**S4 Table.** 　Summary estimates of sensitivity, specificity, AUC of the sROC curve, positive likelihood ratio, and negative likelihood ratio of posthoc sensitivity analysis for perfusion index and pleth variability index

|  | Number of patients (study) | Sensitivity  (95%CI) | Specificity  (95%CI) | AUC of sROC  (95%CI) | Positive likelihood ratio(95%CI) | Negative likelihood ratio(95%CI) | I^2^ |
| --- | --- | --- | --- | --- | --- | --- | --- |
| perfusion index | 1217 (16) | 0.74  (0.70 to 0.79) | 0.64  (0.47 to 0.78) | 0.75  (0.67 to 0.80) | 2.15  (1.38 to 3.44) | 0.42  (0.30 to 0.59) | 23.2% |
| pleth variability index | 262 (4) | 0.67  (0.49 to 0.80) | 0.72  (0.60 to 0.81) | 0.75  (0.64 to 0.83) | 2.39  (1.67 to 3.37) | 0.47  (0.29 to 0.69) | 0.0% |

CI; confidence interval, AUC; area under curve, sROC; summary receiver operating characteristic curve
