# Supplementary figures and images for: Ability of pulse oximetry-derived indices to predict hypotension after spinal anesthesia for cesarean delivery: A systematic review and meta-analysis
Source: PLoS One. 2025 Jan 31;20(1):e0316715. doi: 10.1371/journal.pone.0316715 (PMC11785266; doi:10.1371/journal.pone.0316715)

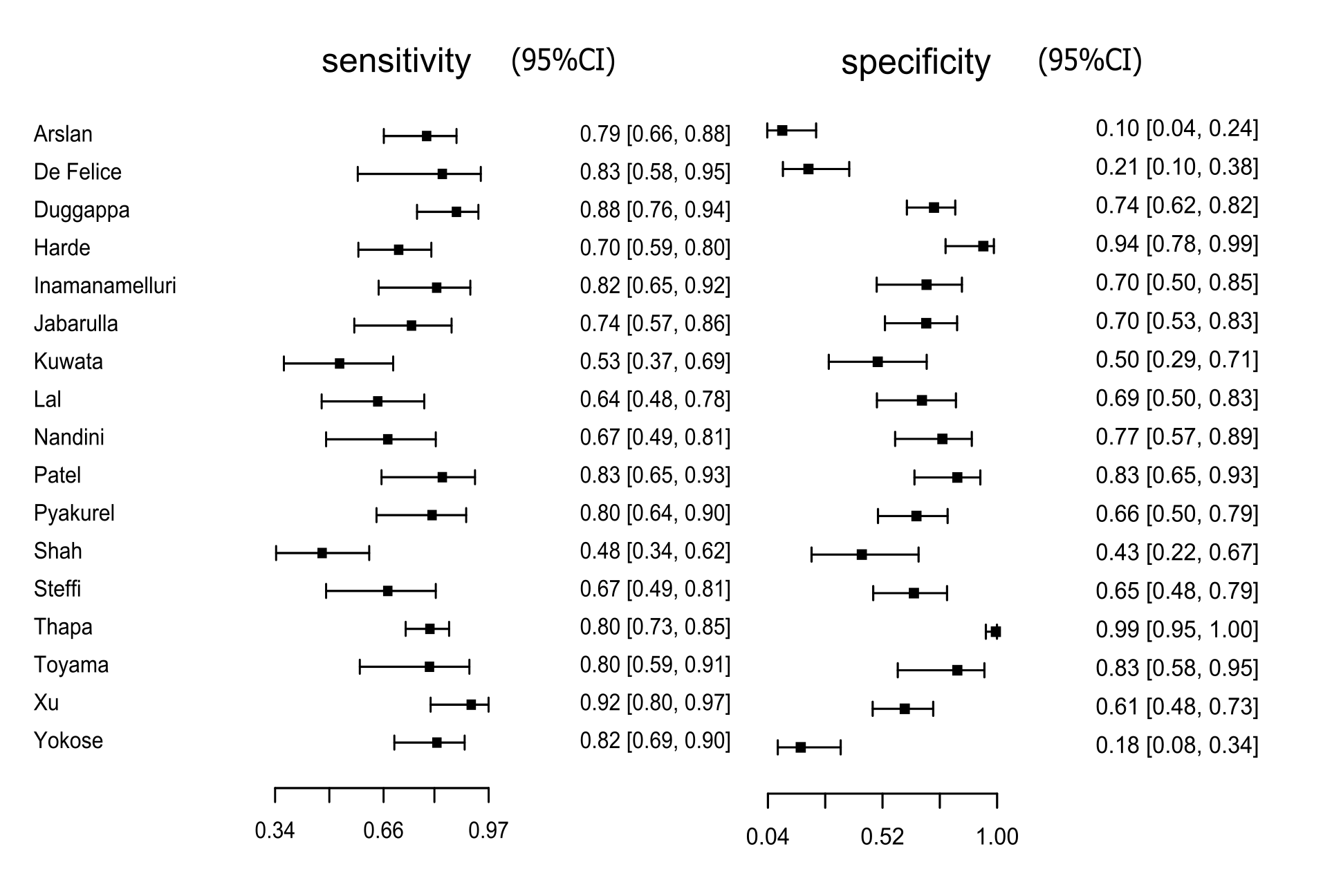

Supplement: S1 Fig — (TIF) [file pone.0316715.s005.tif]

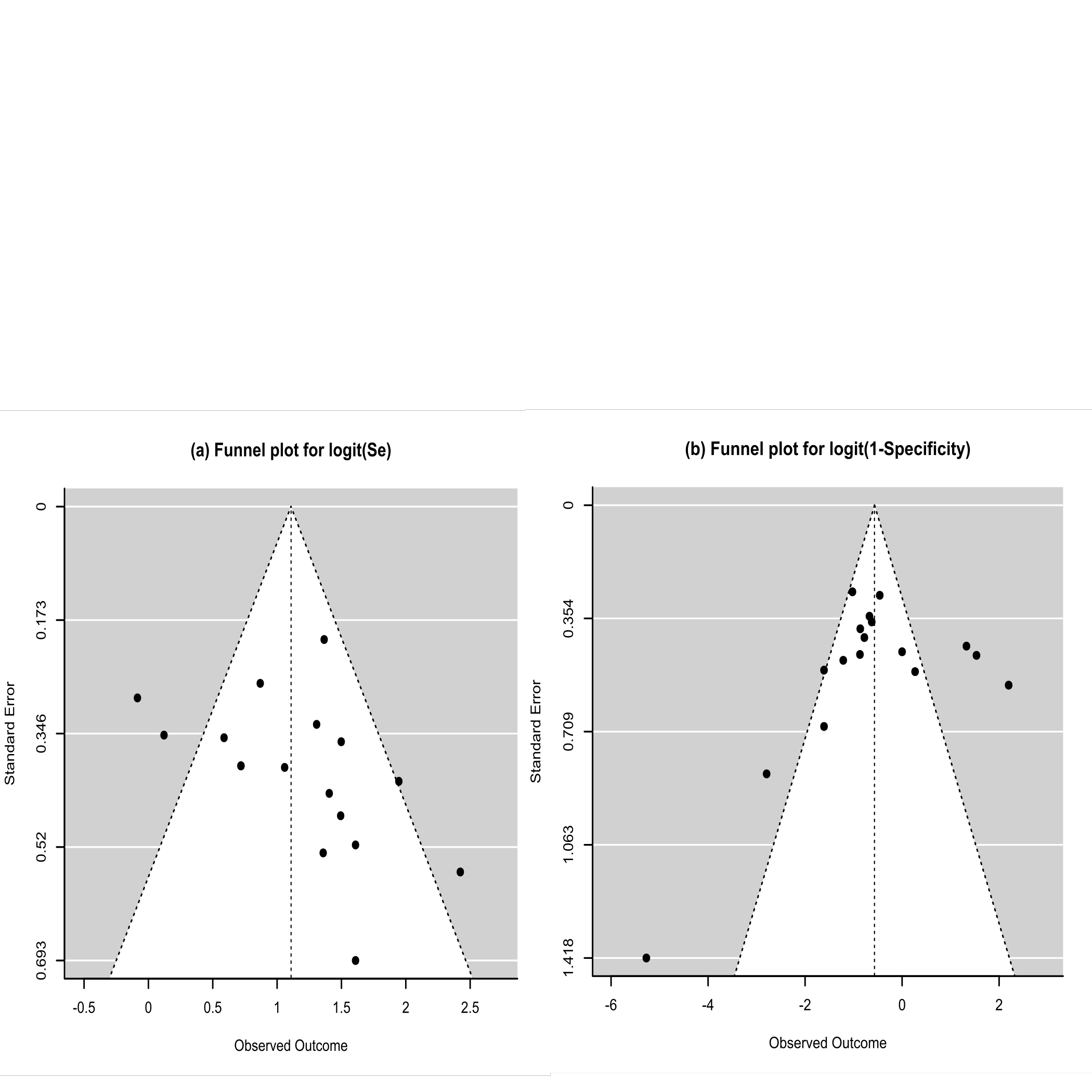

Supplement: S2 Fig — (a) Funnel plot of the sensitivity of perfusion index. Se; sensitivity. (b) Funnel plot of the specificity of perfusion index. (TIF) [file pone.0316715.s006.tif]

sensitivity (95% CI)

specificity (95% CI)

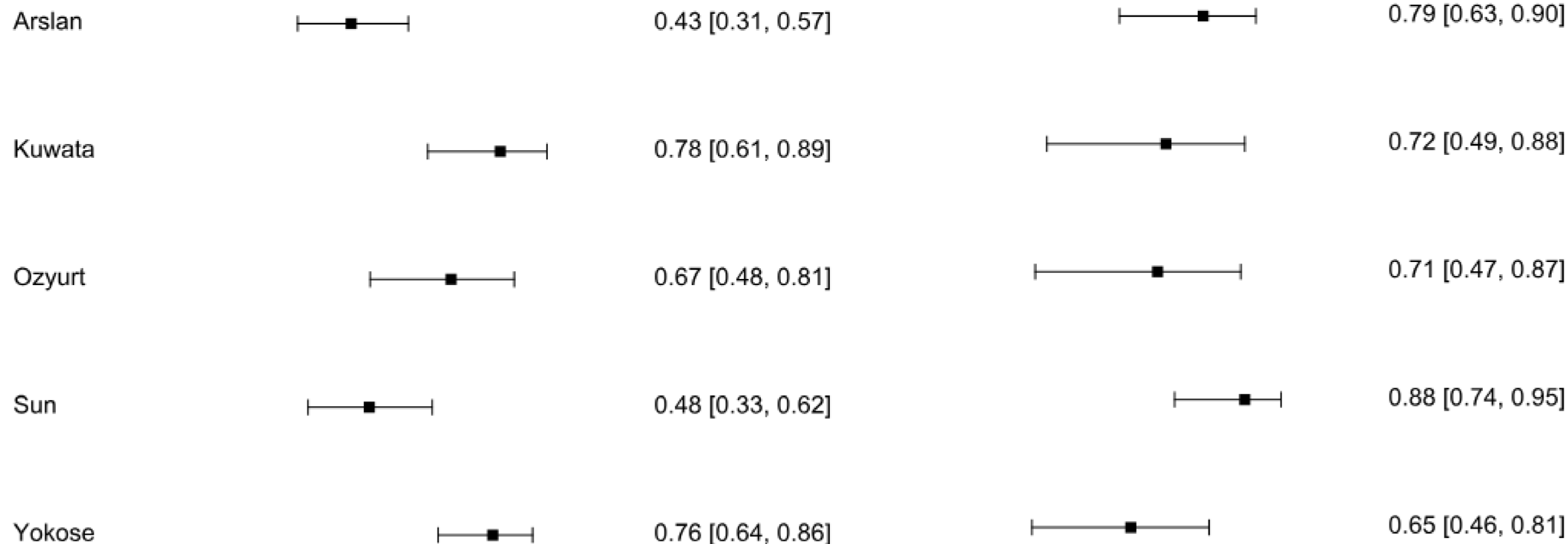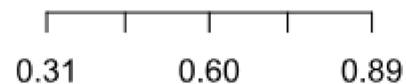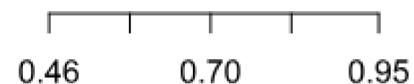

Supplement: S3 Fig — (PDF) [file pone.0316715.s007.pdf]

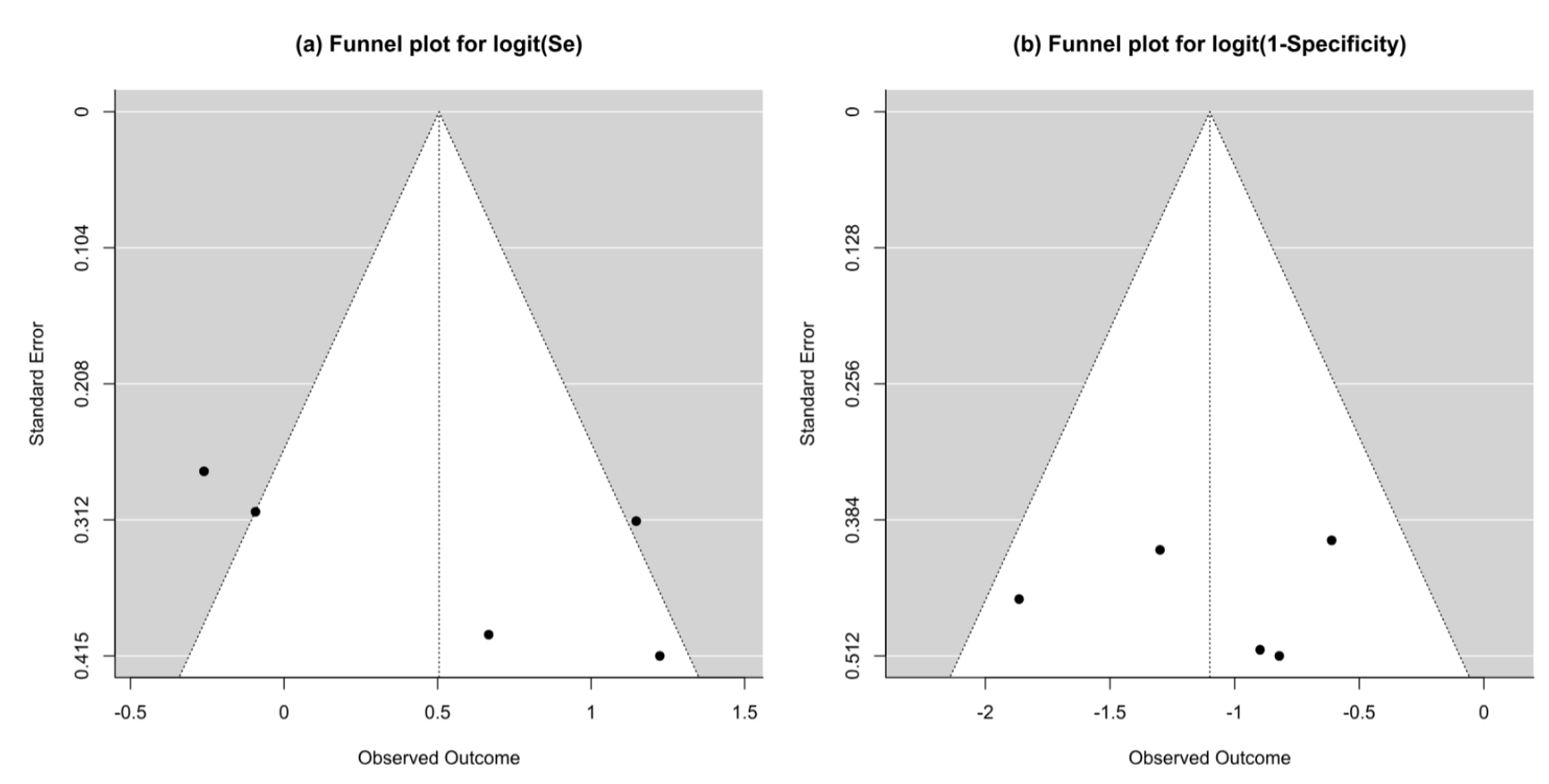

Supplement: S4 Fig — (a) Funnel plot of the sensitivity of pleth variability index. Se; sensitivity. (b) Funnel plot of the specificity of pleth variability index. (TIF) [file pone.0316715.s008.tif]

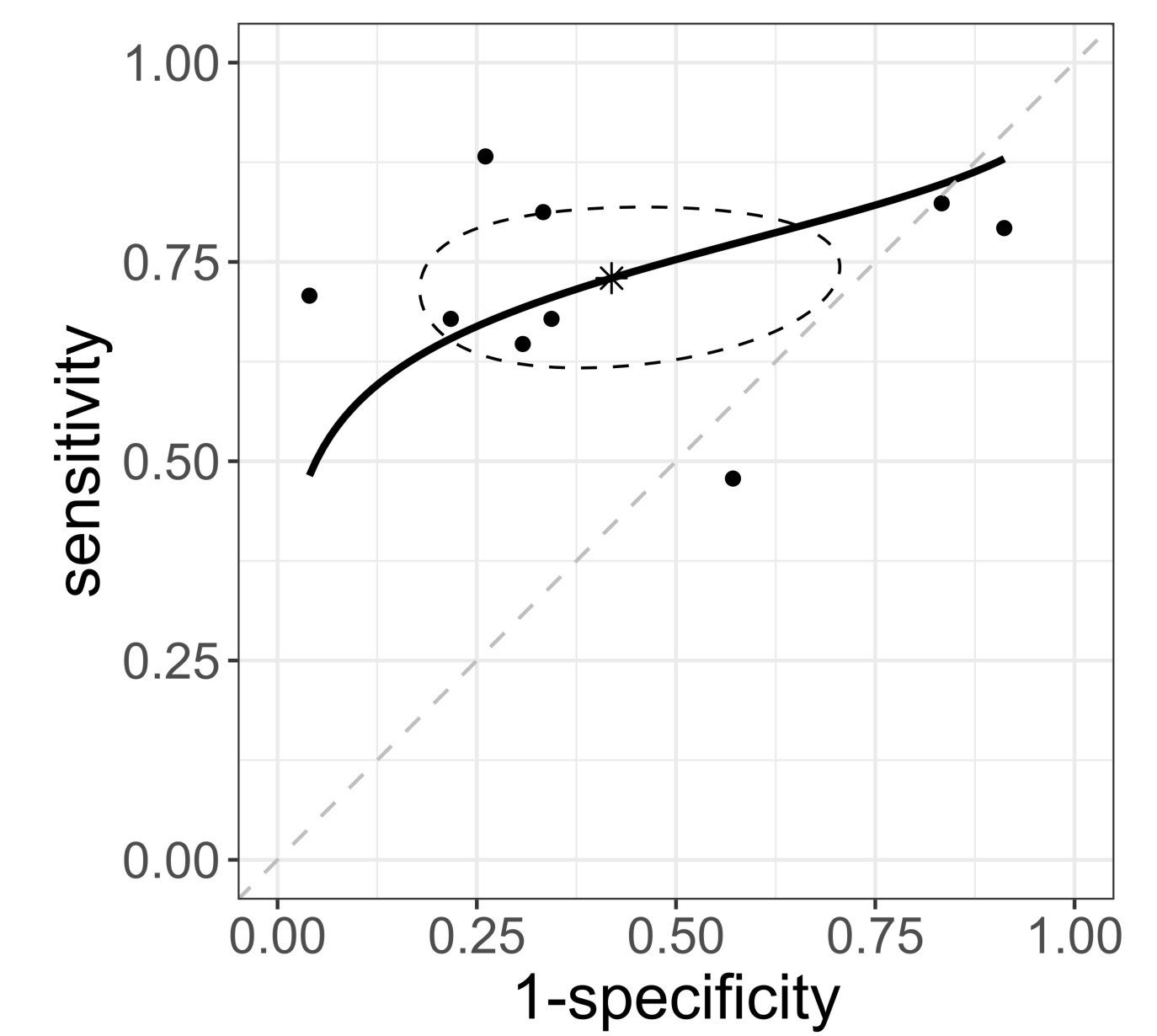

Supplement: S5 Fig — The solid curves represent sROC curves. The dots represent point estimates of sensitivity and 1-specificity for each study included, and the ellipses represent the 95% confidence region. (TIF) [file pone.0316715.s009.tif]

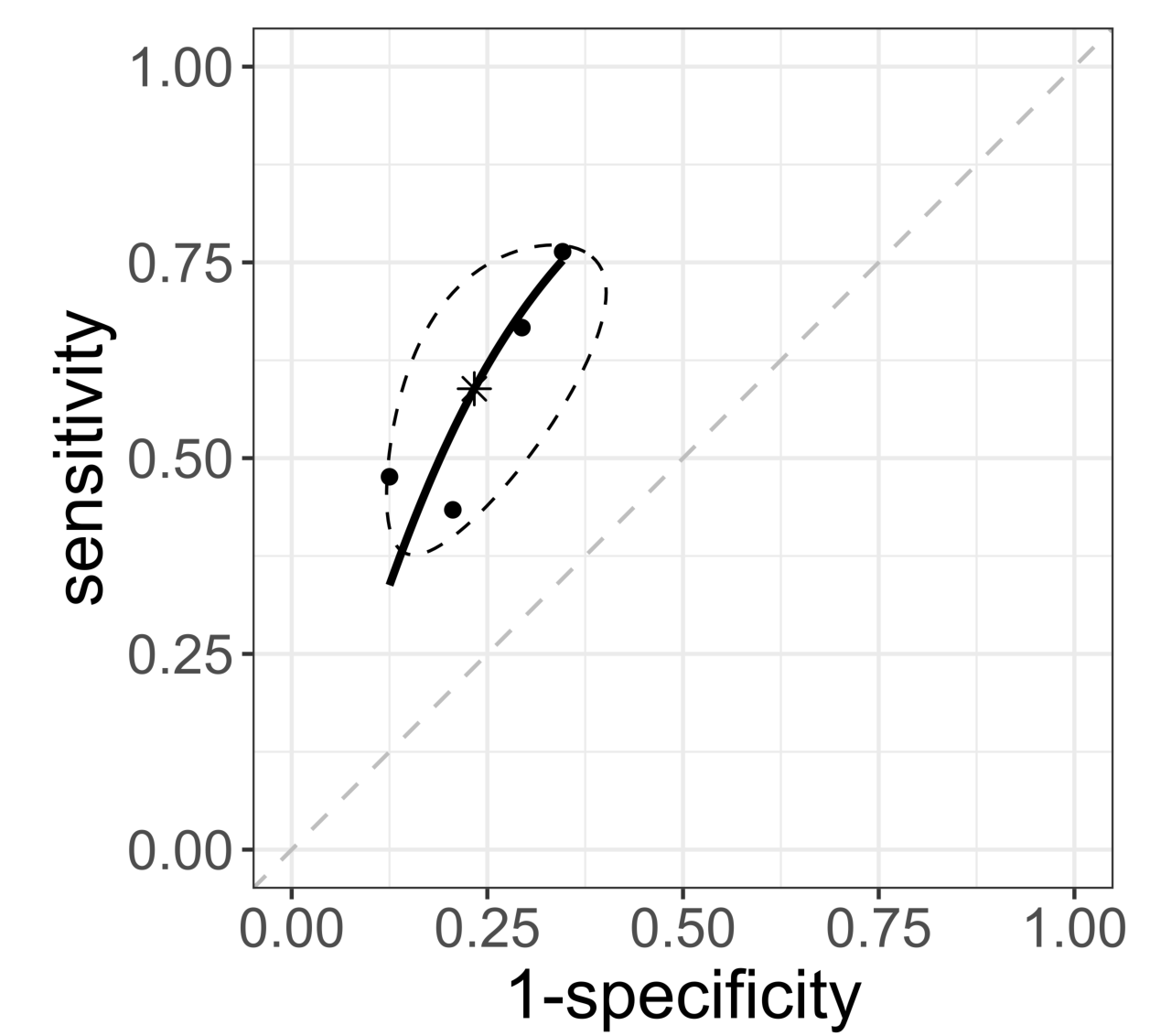

Supplement: S6 Fig — The solid curves represent sROC curves. The dots represent point estimates of sensitivity and 1-specificity for each study included, and the ellipses represent the 95% confidence region. (TIF) [file pone.0316715.s010.tif]

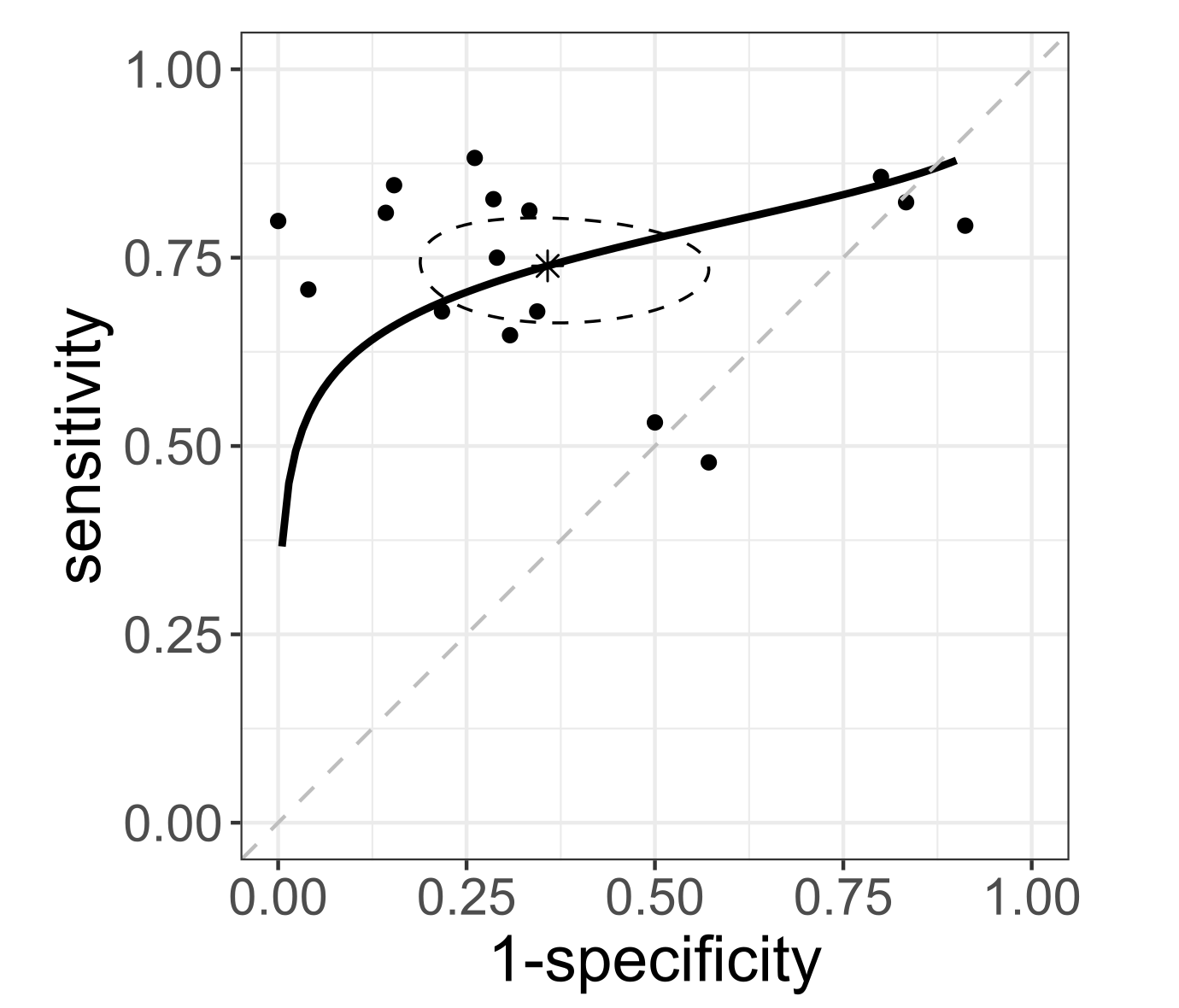

Supplement: S7 Fig — The solid curves represent sROC curves. The dots represent point estimates of sensitivity and 1-specificity for each study included, and the ellipses represent the 95% confidence region. (TIF) [file pone.0316715.s011.tif]

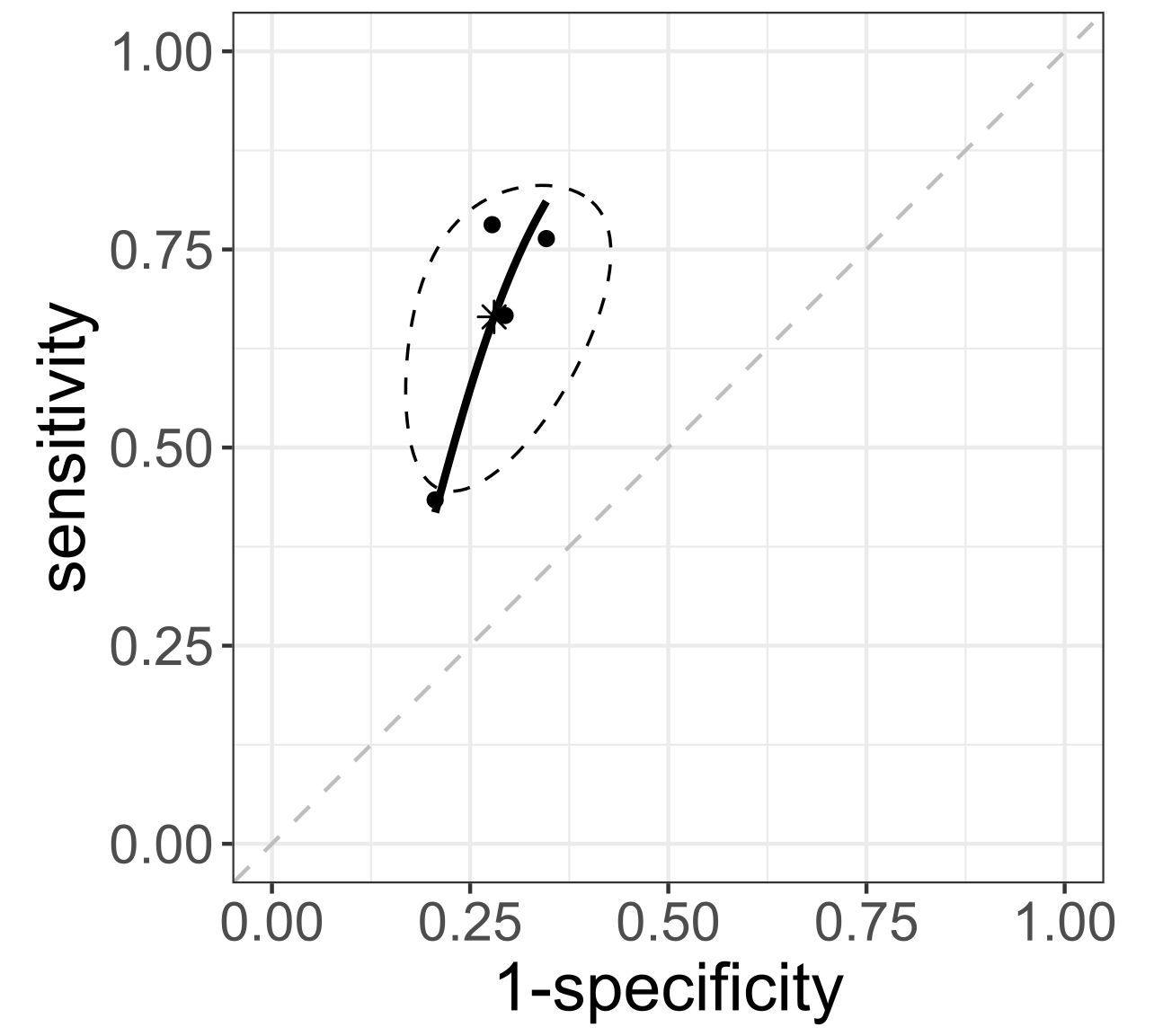

Supplement: S8 Fig — The solid curves represent sROC curves. The dots represent point estimates of sensitivity and 1-specificity for each study included, and the ellipses represent the 95% confidence region. (TIF) [file pone.0316715.s012.tif]
